# Comprehensive analyses of imprinted differentially methylated regions reveal epigenetic and genetic characteristics in hepatoblastoma

**DOI:** 10.1186/1471-2407-13-608

**Published:** 2013-12-27

**Authors:** Janette Mareska Rumbajan, Toshiyuki Maeda, Ryota Souzaki, Kazumasa Mitsui, Ken Higashimoto, Kazuhiko Nakabayashi, Hitomi Yatsuki, Kenichi Nishioka, Ryoko Harada, Shigehisa Aoki, Kenichi Kohashi, Yoshinao Oda, Kenichiro Hata, Tsutomu Saji, Tomoaki Taguchi, Tatsuro Tajiri, Hidenobu Soejima, Keiichiro Joh

**Affiliations:** 1 Department of Biomolecular Sciences, Division of Molecular Genetics & Epigenetics, Faculty of Medicine, Saga University, Nabeshima 5-1-1, Saga 849-8501, Japan; 2 Faculty of Medicine, Sam Ratulangi University, Manado, Indonesia; 3 Department of Pediatric Surgery, Faculty of Medicine, Kyushu University, Fukuoka, Japan; 4 Department of Pediatrics, Toho University, Omori Medical Center, Tokyo, Japan; 5 Department of Maternal-Fetal Biology, National Research Institute for Child Health and Development, Tokyo, Japan; 6 Department of Pathology and Microbiology, Division of Pathology, Faculty of Medicine, Saga University, Saga, Japan; 7 Department of Anatomic Pathology, Pathological Sciences, Graduate School of Medical Sciences, Kyushu University, Fukuoka, Japan; 8 Department of Pediatric Surgery, Graduate School of Medical Science, Kyoto Prefectural University of Medicine, Kyoto, Japan

**Keywords:** Hepatoblastoma, Genomic imprinting, Differentially methylated region, DNA methylation

## Abstract

**Background:**

Aberrant methylation at imprinted differentially methylated regions (DMRs) in human 11p15.5 has been reported in many tumors including hepatoblastoma. However, the methylation status of imprinted DMRs in imprinted loci scattered through the human genome has not been analyzed yet in any tumors.

**Methods:**

The methylation statuses of 33 imprinted DMRs were analyzed in 12 hepatoblastomas and adjacent normal liver tissue by MALDI-TOF MS and pyrosequencing. Uniparental disomy (UPD) and copy number abnormalities were investigated with DNA polymorphisms.

**Results:**

Among 33 DMRs analyzed, 18 showed aberrant methylation in at least 1 tumor. There was large deviation in the incidence of aberrant methylation among the DMRs. *Kv*DMR1 and *IGF2*-DMR0 were the most frequently hypomethylated DMRs. *INPP5Fv2*-DMR and *RB1*-DMR were hypermethylated with high frequencies. Hypomethylation was observed at certain DMRs not only in tumors but also in a small number of adjacent histologically normal liver tissue, whereas hypermethylation was observed only in tumor samples. The methylation levels of long interspersed nuclear element-1 (LINE-1) did not show large differences between tumor tissue and normal liver controls. Chromosomal abnormalities were also found in some tumors. 11p15.5 and 20q13.3 loci showed the frequent occurrence of both genetic and epigenetic alterations.

**Conclusions:**

Our analyses revealed tumor-specific aberrant hypermethylation at some imprinted DMRs in 12 hepatoblastomas with additional suggestion for the possibility of hypomethylation prior to tumor development. Some loci showed both genetic and epigenetic alterations with high frequencies. These findings will aid in understanding the development of hepatoblastoma.

## Background

Hepatoblastoma is the most common primary liver tumor in children, accounting for just over 1% of pediatric cancers and 79% of liver cancers in children under the age of 15 [1]. Most of these tumors are purely derived from epithelium composed exclusively of immature hepatocytic elements, known as fetal and embryonal types. The fetal type consists of smaller than normal hepatocytes that are arranged in irregular laminae, recapitulating those of the fetal liver. The embryonal type is comprised of smaller cells as compared to the fetal type. It has a more immature appearance and pattern of growth. Some of the tumors, referred to as mixed type, are characterized by epithelial patterns and spindled mesenchymal cells. A much rarer variant of such mixed type tumor harbors teratoid features, which contains foci of mature cartilage, intestinal-type or keratinized epithelium, melanin pigment, or skeletal muscle in addition to the elements mentioned above. To date, several genetic and epigenetic features have been observed in hepatoblastoma (reviewed in [2]). The most recurrent cytogenetic abnormalities include the presence of extra copies of chromosomes 2, 8, 20, and the loss of chromosome 4. Mutations or upregulation of the genes involved in embryonic development have been reported. For example, *APC*, *CTNNB1*, *AXIN1*, and *AXIN2* (key factors involved in the Wnt signaling pathway) are frequently mutated, suggesting that aberration of this pathway occurs as an early event during tumorigenesis. Mutation of *PIK3CA*, amplification of *PIK3C2B*, and upregulation of hedgehog ligands and their target genes have also been reported. Epigenetic silencing by promoter hypermethylation occurs at several tumor suppressor genes, such as *SFRP1*, *APC*, *HHIP*, *SOCS1*, *CASP8*, and *RASSF1A*. In addition, several imprinted genes, including *IGF2*, *DLK1*, *PEG3*, *PEG10*, *MEG3*, and *NDN*, have been reported to be overexpressed in hepatoblastoma [2].

Imprinted genes are expressed in a parent-of-origin-specific manner. They are usually clustered in subchromosomal regions called imprinting domains. The human genome contains more than 30 imprinting domains (http://www.geneimprint.com). Imprinting domains have at least one DMR that are characterized by DNA methylation on one of the two parental alleles. There are maternally methylated DMRs and paternally methylated DMRs. In addition, two classes of imprinted DMRs, gametic and somatic, have been described. Gametic DMRs acquire methylation during gametogenesis and the methylation is maintained from zygote to somatic cells during all the developmental stages. Most gametic DMRs are known as imprinting control regions (ICRs) that regulate the imprinted expression of the genes in the domain. By contrast, methylations of somatic DMRs are established during early embryogenesis after fertilization under the control of nearby ICRs [3]. Somatic DMRs also regulate the expression of the imprinted genes.

Many imprinted genes regulate cell growth and differentiation, and, thus, disruption of imprinting, mainly due to aberrant DNA methylation at the responsible DMR, is implicated in pre- and/or post-natal growth disorders and in the pathogenesis of cancers [4]. For example, hypermethylation of *H19*-DMR, which is the ICR of the *IGF2*/*H19* imprinting domain at the 11p15.5 locus, is a cause of Beckwith-Wiedemann syndrome (BWS), the most common overgrowth syndrome characterized by occasional development of embryonal tumors, including hepatoblastoma [5]. The hypermethylation leading to biallelic expression of *IGF2* is seen in a range of tumors, also including hepatoblastoma [6,7]. The LOH of 11p15.5, especially the loss of the maternal allele, is found in approximately 20% of hepatoblastoma cases, and it is reported to be a risk factor for the relapse of this tumor [7,8]. Furthermore, several imprinted genes are overexpressed in hepatoblastoma as mentioned above. Thus, it is speculated that aberrant DNA methylation at imprinted DMRs is a key mechanism during malignant transformation of progenitor cells in a variety of tissues, including the liver [2,9]. However, the methylation status of imprinted DMRs scattered through the human genome has yet to be analyzed comprehensively in hepatoblastoma.

In this study, we performed comprehensive methylation analyses and polymorphism analyses of 33 imprinted DMRs in hepatoblastoma. We therefore describe some epigenetic and genetic characteristics of hepatoblastoma. These findings collectively aid in the understanding of the development of hepatoblastoma.

## Methods

### Samples

Twelve hepatoblastomas and their paired adjacent normal liver tissues were analyzed. Eleven sporadic hepatoblastoma samples (HB01 - HB11) were obtained from the Department of Pediatric Surgery, Faculty of Medicine, Kyushu University, Japan. One hepatoblastoma developed in a BWS patient (BWS109) was obtained from Toho University, Omori Medical Centre, Japan. Histochemical analyses of the tumor tissues indicated that the average of the tumor cell contents was approximately 70%. Ten of the patients were treated based on the Japanese Study Group for Pediatric Liver Tumor-2 (JPLT-2) protocol (HB08 and HB09 were not). Clinical information of the hepatoblastoma cases is shown in Table 1. Three livers (CL7, CL16, CBD1) were used as normal controls. CL7 (a 7-year-old who died from spinal muscular atrophy type I-C with chronic respiratory insufficiency) and CL16 (a 16-year-old who died after head trauma) were provided by the non-profit organization, Human & Animal Bridging Research Organization (Chiba, Japan). CBD1 (a 7-month-old who had congenital biliary dilatation) was obtained from the Department of Pediatric Surgery, Faculty of Medicine, Kyushu University. Written informed consents were obtained from the parents or the guardians of the participants, because the participants were children or dead. This study was approved by the Ethical Committee for Human Genome and Gene Analyses of the Faculty of Medicine, Saga University.

**Table 1:** Clinical information of hepatoblastoma cases

**Case**	**Sex/age**^**a**^	**Histology**	**PRETEXT**	**Preoperative chemotherapy**^**b**^	**POSTTEXT**	**Outcome**	**Other information**
HB01	F/1y3m	Combined fetal and embryonal type	III	CITA4	III	Alive	
HB02	F/3y2m	Fetal type^c^	III	CITA4	III	Aive	
HB03	F/7y11m	Hepatoblastoma (NOS)^d^	III	CITA5	III	Alive	Small for gestational age
HB04	M/1y4m	Mixed epithelial and mesenchymal with teratoid feature^c^	IV	CITA4 + ITEC2	IV	Alive	
HB05	M/1y2m	Mixed epithelial and mesenchymal with teratoid feature	III	CITA5	II	Alive	
HB06	M/10m	Mixed epithelial and mesenchymal with teratoid feature	III	CITA4	III	Alive	
HB07	M/8m	Combined fetal and embryonal type	II	CITA2	II	Alive	
HB08	F/28d	Combined fetal and embryonal type	II			Alive	
HB09	M/1y6m	Combined fetal and embryonal type	II			Treatment related death	Small for gestational age
HB10	F/6y6m	Fetal type	II	CITA2	II	Alive	
HB11	F/3m	Combined fetal and embryonal type	IV	CITA7	III	Treatment related death	
BWS109	F/1y0m	Hepatoblastoma (NOS)^d^	IV,M(+)	CITA7 + ITEC1	IV	Alive	Beckwith-Wiedemann syndrome, liver transplantation at 1 year old

^a^age at diagnosis, ^b^CITA: cisplatin-pirarubicin, ITEC: Ifosfamide, pirarubicin, etoposide, and carboplatin. The numerals indicate the cycle numbers of the chemotherapy. ^c^difficult to diagnose due to chemotherapy, ^d^not otherwise specified.

### DNA isolation and bisulphite conversion

Genomic DNA was extracted from each sample using the QIAamp DNA Mini Kit (Qiagen, Germany) according to the manufacturer’s instructions. One microgram of genomic DNA was subjected to bisulfite conversion using the EZ DNA Methylation Kit^TM^ (Zymo Research, CA), and then the converted DNA was eluted in 100 μl of water.

### MALDI-TOF MS analysis

The methylation status of imprinted DMRs was screened by MALDI-TOF MS analysis with a MassARRAY system (Sequenom, CA) [10], according to the manufacturer’s instructions. MALDI-TOF MS analysis produced signal pattern pairs indicative of non-methylated and methylated DNA. Epityper software analysis of the signals yielded the methylation index which ranged from 0 (no methylation) to 1 (full methylation) of each CpG unit, which contained one or more CpG sites measured as one unit in the MALDI-TOF MS analysis. Aberrant methylation of a CpG unit was defined as when the difference of methylation indexes between two samples exceeded 0.15, which was based on the fact that we have previously found that the differences of *H19*-DMR hypermethylation or *Kv*DMR1 hypomethylation in BWS patients were at least more than 0.15 (data not shown). Since analyzed DMRs included several CpG units, aberrant methylation of a DMR was defined as when more than 60% of total number of analyzed CpG units showed aberrant methylation (with the difference exceeding 0.15). We used CL7 and CBD1 as normal controls in MALDI-TOF MS analysis.

### Pyrosequencing

Pyrosequencing was conducted using QIAGEN PyroMark Q24 according to the manufacturer’s instruction (Qiagen, Germany). Some of the primers for DMR analysis were described by Woodfine et al. [11]. We designed other primers by using PyroMark Assay Design 2.0 (Qiagen, Germany). The primers for LINE-1 (GenBank accession no. X58075) analyses were described by Bollati et al. [12]. The criterion for MALDI-TOF MS analysis was also employed to define the aberrant methylation of each CpG site and an analyzed region. We used three livers, i.e. CL7, CL16, and CBD1, as normal controls in pyrosequencing. The control livers were analyzed in triplicate for LINE-1 and once for DMRs.

### DNA Polymorphism analysis

LOH, UPD, and copy number abnormalities were investigated with DNA polymorphisms. For quantitative analyses, tetranucleotide repeat markers near the imprinted DMRs were amplified and separated by electrophoresis on an Applied Biosystems 3130 genetic analyzer. Data were then quantitatively analyzed with GeneMapper software (Applied Biosystems, CA). The peak height ratios of two parental alleles were calculated. A single nucleotide polymorphism (SNP) of *KCNQ1DN* (*rs229897*) was also analyzed.

All primers used in this study are shown in Additional file 1: Table S1.

### Statistical analysis

The methylation statuses of the samples were compared in three pairs: adjacent normal liver tissue (A) and control livers (C), denoted as AxC; tumors (T) and control livers, denoted as TxC; tumors and adjacent normal liver tissue, denoted as TxA. The binomial distribution test was performed to compare aberrant hypomethylation and aberrant hypermethylation within each comparison pair (AxC, TxC, and TxA). The Chi squared test or Fisher’s exact test was used for comparison between maternally methylated and paternally methylated DMRs and between gametic and somatic DMRs in aberrant hypomethylation and aberrant hypermethylation cases for each comparison pair. For LINE-1 methylation, a paired *t*-test was used to compare tumor and adjacent normal liver tissue, and an independent *t*-test (Welch’s *t*-test) was used for comparing tumor or adjacent normal liver tissue with control liver. A p-value less than 0.05 was considered to be statistically significant. Bonferroni correction was performed when needed.

## Results

### Clinical information of hepatoblastoma cases

Clinical information of the 12 hepatoblastoma cases analyzed in this study are shown in Table 1. Eleven tumors were sporadic (HB01-HB11), and one was associated with BWS (BWS109). The ratio of males to females was 5:7. The mean age at diagnosis was 25.7 months, ranging from 28 days to 7 years and 11 months. In terms of histological features, 5 cases had combined fetal and embryonal types, 3 cases had mixed epithelial and mesenchymal features with teratoid features, 2 cases were fetal types, and 2 cases were hepatoblastomas (not otherwise specified). Using PRETEXT staging [13], 4 cases were stage II, 5 cases were stage III, and 3 cases were stage IV. Ten of twelve cases were undergoing chemotherapy based on the JPLT-2 protocol, but only two cases (HB05 and HB11) regressed to a lower stage after chemotherapy.

### Analyses of aberrant methylation and genetic alterations at imprinted DMRs

We selected 33 regions reported previously as imprinted DMRs in the human genome [11,14] (refer to http://www.geneimprint.com/). Our strategy in this study involved screening the methylation levels of DMRs in tumors, their paired adjacent normal tissues, and normal control livers by MALDI-TOF MS. The samples that showed aberrant methylation were analyzed again by pyrosequencing to confirm the result. These two methods are the most reliable methods of methylation analysis at present [10,15,16]. First, we analyzed the methylation level of these regions in two normal livers, i.e. CL7 and CBD1, by MALDI-TOF MS (Additional file 2: Figure S1). A total of 20 DMRs showed almost 50% methylation, however, 8 DMRs (*IGF2R*-DMR2, *IGF2*-DMR0, *IGF2*-DMR2, IG-DMR-CG4, IG-DMR-CG6, *TCEB3C*-DMR, *USP29*-DMR, and *NNAT*-DMR) showed mostly full methylation, and 5 DMRs (*TP73*-DMR, *SPTBN1*-DMR, *WT1-AS*-DMR, *DLK1*-DMR, and *GNASXL*-DMR) showed mostly no methylation. It is highly possible that these regions were not differentially methylated in the normal liver, probably due to tissue-specific and/or age-related features of differential methylation, because most of the regions were also analyzed by pyrosequencing and their methylation statuses were confirmed (Additional file 2: Figure S1).

**Figure 1:**
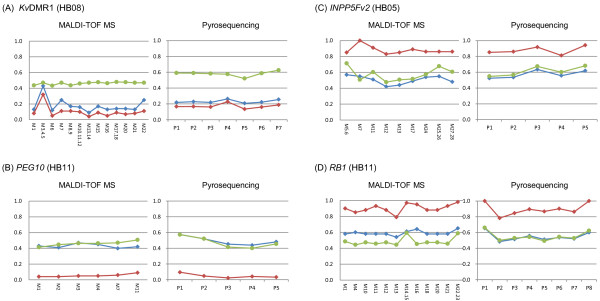
**Representative results of methylation analyses by MALDI-TOF MS and pyrosequencing. (A and B)** Representative samples of aberrant hypomethylation. *Kv*DMR1 of HB08 and PEG10 of HB11 are shown. *Kv*DMR1 was hypomethylated in both adjacent normal liver and tumor tissues, whereas *PEG10*-DMR was hypomethylated only in tumor tissue. **(C and D)** Representative samples of aberrant hypermethylation. *INPP5Fv2*-DMR in HB05 and *RB1*-DMR in HB11 are shown. Only tumors showed hypermethylation at these DMRs. Aberrant methylation of a DMR was defined as when more than 60% of total CpG units or CpG sites were aberrantly methylated. Aberrant methylation of a CpG unit or CpG site was defined as occurring when the difference of its methylation indexes in two samples exceeded 0.15. The vertical axis represents the methylation index; the horizontal axis represents CpG units (MALDI-TOF MS) or CpG sites (pyrosequencing) analyzed. Green line: average of normal control livers; blue line: adjacent normal liver; red line: tumor (hepatoblastoma).

Next, we screened the methylation status of the 33 DMRs in 12 hepatoblastomas and their paired adjacent normal liver tissue by MALDI-TOF MS. We found aberrant methylation in tumors and also in adjacent liver tissue by comparing the methylation between tumors and normal controls (TxC), tumors and adjacent liver tissue (TxA), and adjacent liver tissue and normal controls (AxC). The definition of aberrant methylation is described in the Methods section. After excluding samples harboring chromosomal abnormalities as described later, we confirmed the aberrant methylation using pyrosequencing, except in the case of *H19*-DMR (representative data is shown in Figure 1 and all data in Additional file 3: Figure S2). Additional normal control liver, CL16, was used in pyrosequencing analyses. The methylation status of *H19*-DMR was analyzed by hot-stop combined bisulfite restriction analysis (COBRA) [17] or bisulfite sequencing because of the difficulty in the primer-design for pyrosequencing (Additional file 4: Figure S3).

**Figure 2:**
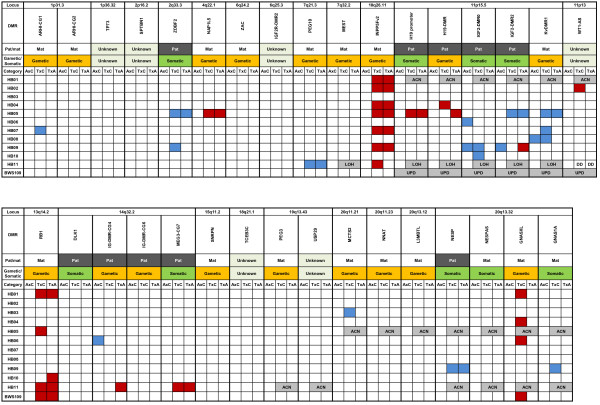
**Aberrant methylations and genetic alterations of 33 imprinted DMRs in 12 hepatoblastomas.** Aberrant hypomethylation and aberrant hypermethylation found in each comparison are indicated by blue and red boxes, respectively. Aberrant methylation was identified by comparing adjacent normal liver tissue with normal control livers: AxC; tumors compared with normal control livers: TxC; and tumors compared with adjacent normal livers: TxA. The classes of these DMRs in previous reports are shown in Pat/Mat and Gametic/Somatic rows. *ZDBF2*-DMR was tentatively assigned as a somatic DMR based on experiments from mice [35]. MALDI-TOF MS revealed that 13 DMRs, such as *TP73*-DMR, *SPTBN1*-DMR, *IGF2R*-DMR2, *IGF2*-DMR0, *IGF2*-DMR2, *WT1-AS*-DMR, *DLK1*-DMR, IG-DMR-CG4, IG-DMR-CG6, *TCEB3C*-DMR, *USP29*-DMR, *NNAT-*DMR, and *GNASXL*-DMR did not show differential methylation in control livers. Most of these methylation statuses were confirmed by pyrosequencing analysis. Mat: maternally methylated DMR; Pat: paternally methylated DMR; gametic: gametic DMR; somatic: somatic DMR. ACN: abnormal copy number; LOH: loss of heterozygosity; UPD: paternal uniparental disomy; DD: difficult to decide.

In order to exclude aberrantly methylated DMRs, as associated with chromosome abnormalities, such as UPD or copy number abnormality, DNA polymorphism analyses using microsatellites and SNPs were performed on all regions showing aberrant methylation in the MALDI-TOF MS analyses. We found seven genetic alterations in four tumors resulting in aberrant methylation: abnormal allelic copy number of 11p13-p15.5 in HB01, 20q11-q13 in HB05, and 19q13 and 20q13 in HB11; LOH of 7q32.2 and 11p15.5 in HB11; and paternal UPD of 11p13-p15.5 in BWS109 (Figure 2). We speculated the allelic imbalance statuses of these loci according to the results of MALDI-TOF MS analysis and DNA polymorphism analysis (Additional file 5: Figure S4). HB01 would harbor more paternal copies than the maternal copies in 11p13-p15.5. An abnormal allelic copy number of 20q11-q13 in HB05 would represent a higher copy number in the maternal allele than the paternal allele. HB11 would have more maternal copies of 19q13 and more paternal copies of 20q13. LOH of 7q32.2 and 11p15.5 would have occurred due to a paternal deletion and a maternal deletion, respectively. The paternal UPD was confirmed using the parents’ DNA (Additional file 5: Figure S4). The extent of the UPD was found to be at 11p11.2–pter by a SNP array analysis (data not shown).

The following results described are shown in Figure 2. All tumors carried aberrant methylation in at least one DMR. HB05 carried aberrant methylations at 8 DMRs, the highest number of aberrant methylations, whereas HB03 and HB08 carried aberrant methylations at only one DMR, *MCTS2*-DMR and *Kv*DMR1, respectively. A total of 18 of 33 DMRs showed aberrant methylation, whereas 15 DMRs did not show such features in any tumors. There was large deviation in the incidence of aberrant methylation among the DMRs. *Kv*DMR1 and *IGF2*-DMR0 were the most frequently hypomethylated DMRs in 3 of 9 tumors. The most frequently hypermethylated DMR was *INPP5Fv2*-DMR, which occurred in 7 of 12 samples. *RB1*-DMR was also hypermethylated with a high frequency, which occurred in 5 of 12 samples. In addition, *GNASXL*-DMR was hypermethylated in 4 of 10 samples. The following DMRs showed aberrant methylation in only one tumor: *ARH1*-CG1, *NAP1L5*-DMR, *PEG10*-DMR, *H19* promoter, *WT1-AS*-DMR, *MEG3-CG7*-DMR, *MCTS2*-DMR, *NESP*-DMR, and *GNAS1A*-DMR.

Two chromosomal loci, 11p15.5 and 20q13.32, showed high frequencies of genetic and epigenetic alterations at 10 of 12 and 7 of 12, respectively. In the 11p15.5 locus, seven tumors carried the aberrant methylation and three samples carried genetic alterations. In the 20q13.32 locus, five tumors carried aberrant methylation and two carried an abnormal allelic copy number.

### Comparisons of aberrantly methylated DMRs

**Figure 3:**
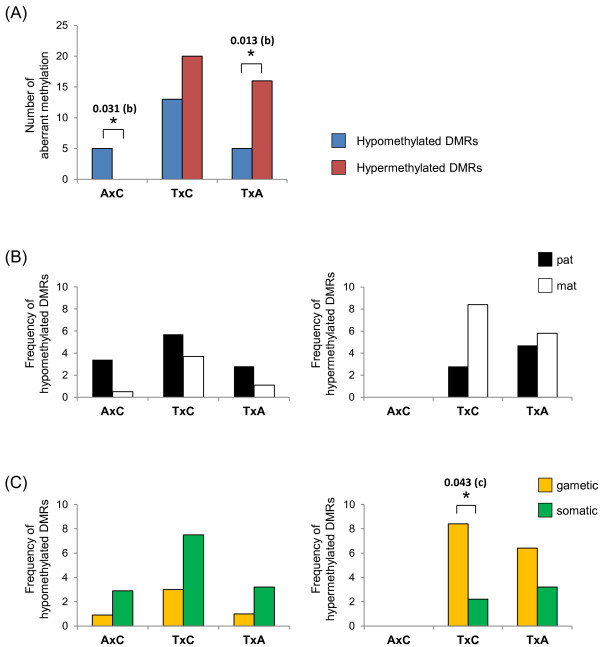
**Comparison of aberrant methylations of DMRs in hepatoblastoma. (A)** Comparison of the numbers of aberrantly hypomethylated (blue bars) and hypermethylated (red bars) DMRs. Hypomethylation, but not hypermethlation, was observed in adjacent normal tissues. Hypermethylation was observed more frequently than hypomethylation in tumors compared to adjacent normal tissues. **(B)** Comparison of the frequencies of aberrant methylations between the paternally methylated DMRs (black bars) and the maternally methylated DMRs (white bars). Left and right panels indicate hypomethylation and hypermethylation, respectively. There was no significant difference in both hypomethylation and hypermethylation. **(C)** Comparison of the frequencies of aberrant methylations between gametic DMRs (yellow bars) and somatic DMRs (green bars). Left and right panels indicate hypomethylation and hypermethylation, respectively. Hypermethylation was observed more frequently at gametic DMRs with statistically significant difference in tumors compared to control livers. The frequencies were compared in **(B)** and **(C)** because of the difference in the total numbers of DMRs in the two categories compared; that is, the paternal and the maternal DMRs or the gametic and the somatic DMRs. The asterisks indicated significant difference. The p values are shown above the comparisons. b: binomial test, c: chi squared test.

We compared the numbers of aberrantly hypomethylated and hypermethylated DMRs in three pairs of the sample groups (Figure 3A). We excluded the DMRs harboring UPD or copy number abnormalities for the statistical analyses. Comparing adjacent normal liver tissues (A) and control livers (C), herein denoted as AxC, only hypomethylation was observed in adjacent normal liver tissue (p = 0.031). In the TxC comparison, both hypermethylation and hypomethylation were observed in tumors (no significant difference). In the TxA comparison, hypermethylation was observed more frequently than hypomethylation with statistical significance (p = 0.013). In addition, the number of hypomethylated DMRs in tumors was higher than that of adjacent normal liver tissue (p = 0.040), although Bonferroni correction did not show statistical significance of the difference with a significance level of 0.05/3 (approximately 0.0167). These results suggested a possibility that hypomethylation occurred at certain DMRs in adjacent normal liver tissue prior to tumor development, whereas hypermethylation occurred only within the tumor tissue itself.

We further compared the frequencies of aberrant methylation between paternally methylated DMRs and maternally methylated DMRs. As for hypomethylation, there was no significant difference between the two kinds of DMRs in each comparison (Figure 3B, left panel). In contrast, hypermethylation, which was observed only in tumors, tended to occur more frequently at maternally methylated DMRs than paternally methylated DMRs in the TxC comparison (p = 0.060) (Figure 3B, right panel). We also compared the frequencies of aberrant methylation between gametic DMRs and somatic DMRs. No significant difference in hypomethylation was found between the two kinds of DMRs in each comparison (Figure 3C, left panel). In contrast to hypomethylation, hypermethylation occurred at gametic DMRs more frequently with statistical significance (p = 0.043) (Figure 3C, right panel). This difference was mainly due to the frequent occurrence of hypermethylation at three maternally methylated and gametic DMRs, such as *INPP5Fv2*-DMR, *RB1*-DMR, and *GNASXL*-DMR (Figure 2).

### Methylation status of LINE-1 in hepatoblastoma

**Figure 4:**
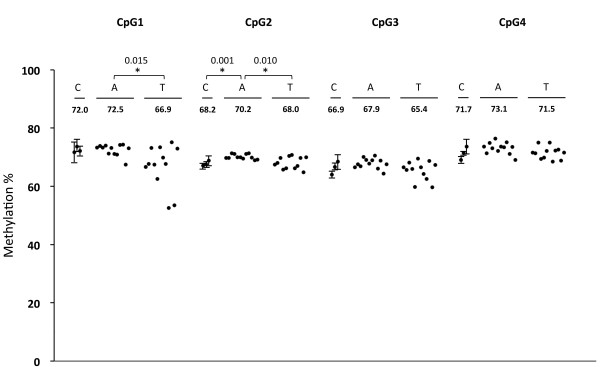
**Methylation status of LINE-1.** Methylation levels of four successive CpG sites in LINE-1 were analyzed in hepatoblastomas (T), paired adjacent normal liver tissues (A), and three normal control livers (C) by pyrosequencing. The normal controls were analyzed in triplicate and the average values were plotted with standard deviations. The average values of methylation (%) are shown for each sample groups. The asterisks indicated significant differences (p < 0.0167). Bonfferoni correction was applied for this statistical analysis.

We analyzed the methylation status of LINE-1 in all samples by pyrosequencing to assess the genome-wide methylation level. LINE-1 is a human repetitive element and constitutes approximately 30% of the human genome [18]. Its methylation status has been used as a surrogate marker for genome-wide DNA hypomethylation in many cancers [19,20]. We analyzed the methylation of four CpG sites in a LINE-1 sequence that were hypomethylated in cancers [21,22]. We compared the methylation levels of each CpG site among the three groups using Bonferroni correction with significance level of 0.0167 (Figure 4). Tumors showed slight hypomethylation only at CpG1 among four CpGs (p = 0.015 in TxA). However, other CpG sites did not show hypomethylation although bare hypermethylations (less than 2.5%) was found only in adjacent normal liver tissues at CpG2 (p = 0.001 in AxC and p = 0.010 in AxT). These results suggested that the genome-wide methylation levels were almost same among three sample groups.

## Discussion

In this study, we found many imprinted DMRs methylated aberrantly in hepatoblastomas and paired adjacent normal liver tissue. An important finding was that the aberrant hypomethylation occurred not only in tumor tissue but also in adjacent normal liver tissue. One possible explanation is that the occurrence of the aberrant hypomethylation at certain DMRs may be a very early and specific event prior to tumor development, although there is another possibility that the tumor may induce methylation changes in the adjacent tissues. Okamoto et al. have previously reported a similar phenomenon with respect to aberrant hypermethylation of *H19*-DMR that was frequently found in normal tissues adjacent to Wilms’ tumors, which carried the same aberrant methylation [23]. Based on the results, it was hypothesized that the preceding aberrant methylation may be a constitutional aberration in the onset of embryonal tumors. In contrast to the hypomethylation, the aberrant hypermethylation of the DMR occurred only in tumors. These results indicated that the hypermethylation of the DMRs, especially for *INPP5Fv2*-DMR, *RB1*-DMR, and *GNASXL*-DMR, was a specific event for tumor development; this suggested that the pre-cancerous cells did not carry hypermethylation at the DMRs, but acquired the aberrant methylation during tumor development.

We also analyzed the genome-wide methylation level, represented by LINE-1 methylation, and we did not find large difference among three sample groups as a whole. LINE-1 is usually hypomethylated in many adult tumors, and its methylation level correlates with clinicopathological features of the tumors [19]. The different situation concerning LINE-1 methylation between hepatoblastoma and adult tumors may reflect a different mechanism of tumorigenesis in embryonal tumors as compared to adult tumors.

Hypermethylation in tumors was frequently observed at three DMRs, *INPPF5v2*-DMR, *RB1*-DMR, and *GNASXL*-DMR. *INPPF5v2*-DMR controls the expression of *INPP5F* transcript variant 2, which encodes a protein of an unknown function [24,25]. *RB1*-DMR, located in intron 2 of the *RB1* gene, leads to maternal expression of transcript variants from exon 2B with very low expression in normal tissues [26]. The function of the variants in cell proliferation is not known. Thus, the effect of these hypermethylated DMRs on tumorigenesis would be little or unknown. *GNASXL*-DMR is associated with the paternal expression of *GNASXL*, which encodes a protein involved in signal transduction [27-29]. The DMR was shown to be mostly unmethylated in control livers (Additional file 2: Figure S1). Thus, hypermethylation of GNASXL-DMR would reduce expression of *GNASXL*. Unfortunately, the expression of genes linked to aberrantly methylated DMRs could not be analyzed due to poor RNA quality, which was probably due to effects of chemotherapy and a limited amount of samples. Therefore, we could not assess the involvement of hypermethylation in tumorigenesis of hepatoblastoma.

Another important finding was the frequent occurrence of both genetic and epigenetic alterations at the two chromosomal loci, 11p15.5 and 20q13.3. The 11p15.5 locus is a well-known imprinted locus responsible for BWS, a tumor-predisposing imprinting disorder. The locus was found to be altered genetically and/or epigenetically in 10 of 12 tumors. Hypermethylation at *H19*-DMR and hypomethylation at *IGF2*-DMR0 associated with biallelic expression of *IGF2* were reported in adult and embryological tumors, including hepatoblastoma [6,7]. Hypermethylation at *H19*-DMR and the *H19* promoter also reduced the expression of *H19* in Wilms’ tumor [30,31]. In addition to epigenetic alterations, genetic alterations, such as the amplification of paternal alleles leading to overexpression of *IGF2* and LOH of the maternal allele leading to reduced expression of *H19*, were observed in sporadic Wilms’ tumors [32,33]. In this study, in addition to the hypermethylations at *H19*-DMR and the *H19* promoter in two tumors, hypomethylation at *IGF2*-DMR0 occurred in another two adjacent normal liver tissues. Further, abnormal allelic copy number, paternal UPD, and maternal LOH of 11p15.5 were observed. The overexpression of *IGF2* and the reduced expression of *H19* would play an important role in tumorigenesis of hepatoblastoma.

The 20q13.3 locus was also altered genetically and/or epigenetically in 7 of 12 tumors. This locus is responsible for pseudohypoparathyroidism, a condition in which pathogenesis is attributed to the tissue specific imprinting of *Gsα*, for example, which occurs in the proximal renal tubule. On the other hand, an extra copy of chromosome 20 has been known to be the most recurrent cytogenetic alteration in hepatoblastoma [2,34]. We found copy number differences of the alleles in this region, suggesting the existence of non-imprinted oncogenic gene(s) in this region.

Many epigenetic and genetic alterations were found at the loci linked to the 33 imprinted DMRs in 12 hepatoblastomas. However, since sample numbers in this study were small, more hepatoblastoma samples should be analyzed to confirm the present data and to evaluate the precise role of these alterations in tumorigenesis in addition to assessing their usefulness as markers for clinical characteristics, such as stage classification, response to chemotherapy, and prognosis. Also needed are the expression analyses of the genes linked to aberrantly methylated DMRs to assess their role in tumor development, although it is very difficult to obtain hepatoblastoma samples without any chemotherapeutic history.

## Conclusions

We found epigenetic and genetic characteristics of hepatoblastoma by comprehensive epigenetic and genetic analyses of 33 DMRs linked to imprinting loci in 12 hepatoblastoma samples and their adjacent normal liver tissues. These included aberrant hypomethylation in adjacent normal liver tissue, tumor-specific hypermethylation, and the frequent occurrence of both genetic and epigenetic alterations at 11p15.5 and 20q13.3 loci. Further studies using more hepatoblastoma samples are needed to confirm the present results and evaluate their roles in the tumor development.

## Abbreviations

DMR: Differentially methylated region; LOH: Loss of heterozygosity; UPD: Uniparental disomy; LINE-1: Long interspersed nuclear element-1; ICR: Imprinting control regions; BWS: Beckwith-Wiedemann syndrome; MALDI-TOF MS: Matrix-assisted laser desorption/ionization time-of-flight mass spectrometry; SNP: Single nucleotide polymorphism; COBRA: Combined bisulfite restriction analysis.

## Competing interests

The authors declare that they have no competing interests.

## Authors’ contributions

JMR made significant contributions to the acquisition and analysis of data and also helped in manuscript preparation. TM, KH^1^, HY, and KN^1^ made contributions to technical supports and data analyses. RS, KM, RH, KK, YO, TS, TT^3^, and TT^8^ prepared the tissue samples. KN^5^ and KH^5^ performed technical support and statistical analyses. SA performed HE analyses of tumor samples. HS conceived the study, participated in its design and supervision and prepared the manuscript. KJ also participated in the design and supervision of the study and the preparation of the manuscript. All authors read and approved the final manuscript.

## Pre-publication history

The pre-publication history for this paper can be accessed here:

http://www.biomedcentral.com/1471-2407/13/608/prepub

## Supplementary Material

Additional file 1: Table S1 Primers used for this study. Click here for file

Additional file 2: Figure S1 Maps of DMRs analyzed in this study and their methylation status in normal control livers. Upper part; The arrow represents the position and the direction of the transcription start site (TSS). P: promoter; Cen: centromere; Tel: telomere; yellow box (CGI): CpG island; orange boxes (M): CpG sites analyzed by MALDI-TOF MS; green boxes (P): CpG sites analyzed by pyrosequencing. Numbers with diagonal lines indicate CpG units (MALDI-TOF MS) or CpG sites (pyrosequencing), which could not be analyzed. Figures are not drawn to scale. Lower part; Results of MALDI-TOF MS and pyrosequencing are shown. In methylation graphs, the vertical axis represents the methylation index (0: no methylation; 1: full methylation). The horizontal axis represents CpG units or CpG sites. CL7 was analyzed in duplicate by MALDI-TOF MS analysis. Blue and red lines: CL7; green line: CBD1; dark grey line: CL16. Click here for file

Additional file 3: Figure S2 Methylation data of the aberrantly methylated DMRs in hepatoblastomas. The results of MALDI-TOF MS (left panel) and pyrosequencing (right panel) are shown. The vertical axis represents the methylation index (0–1); the horizontal axis represents CpG units (MALDI-TOF MS) or CpG sites (pyrosequencing). Green line: average of control livers; blue line: adjacent normal liver; red line: tumor (hepatoblastoma). Click here for file

Additional file 4: Figure S3 Methylation status of *H19*-DMR as determined by bisulphite cloning sequencing and hot-stop COBRA. (A) Bisulphite sequencing of HB05, which was heterozygous for *rs2071094*. Filled circle: methylated CpG site; open circle: unmethylated CpG site. *rs2071094*: single nucleotide polymorphisms (A/T). CTCF6: CTCF binding site 6. *Taq*I: *Taq*I site used for hot-stop COBRA. (B) Hot-stop COBRA. End-labeled PCR products were obtained by PCR with ^32^P labeled reverse primer in the final amplification cycle. The PCR products were digested with *Taq*I overnight and then electrophoresed. Band intensities were quantitated using the FLA-7000 fluoro-image analyzer (Fujifilm, Japan). un: unmethylated control DNA; me: fully methylated control DNA. Click here for file

Additional file 5: Figure S4 Genetic alterations in hepatoblastoma. (A) Map of 11p15-p13 is shown uppermost. Black box: microsatellite marker; white box: DMR analyzed. Tel: telomere; Cen: centromere. Figure is not drawn to scale. Below the map, the representative data of the methylation analyses and microsatellite analyses are shown for three hepatoblastomas. For HB01 tumor, a high paternal copy number was estimated because of the hypermethylation at the paternally methylated *H19*-DMR and the hypomethylation at the maternally methylated *Kv*DMR1. LOH in HB11 tumor was indicated by the near loss of one of two alleles. The maternal allele could have been lost because of hypermethylation at *H19*-DMR and hypomethylation at *Kv*DMR1. The deviation of the allelic ratio in adjacent normal liver and tumor tissue indicates paternal UPD mosaicism in BWS109, whereas the allelic ratios in the parental blood were approximately 1. The level of mosaicism was higher in tumor than in adjacent normal liver tissue. In tumor samples, the value of the higher peak was divided by that of the lower peak. In adjacent normal liver and parental samples, the ratios were calculated following the pattern in their related tumor. (B) LOH of 7q32 in HB11 tumor was indicated. Because of the hypermethylation at the maternally methylated *MEST*-DMR, the paternal allele would have been lost. (C) Higher maternal copy number of 19q13 were suggested in HB11 tumor, based on the allelic ratio of *D19S589* and the hypermethylation at the maternally methylated *PEG3*-DMR. (D) Allelic copies of 20q11-q13 in HB05 and HB11 tumors were suggested to be abnormal by the allelic ratios of *D20S438* and *D20S158*. Based on the abnormal methylations at the paternally methylated *NESP*-DMR, HB05 tumor would carry more maternal copies than paternal copies of the locus, whereas in HB11 tumor, the paternal allelic copy number would be higher. (PDF 584 kb) Click here for file
