# Supplementary material for: Comprehensive analyses of imprinted differentially methylated regions reveal epigenetic and genetic characteristics in hepatoblastoma
Source: BMC Cancer. 2013 Dec 27;13:608. doi: 10.1186/1471-2407-13-608 (PMC3880457; doi:10.1186/1471-2407-13-608)
Supplement: Additional file 2: Figure S1 — Maps of DMRs analyzed in this study and their methylation status in normal control livers. Upper part; The arrow represents the position and the direction of the transcription start site (TSS). P: promoter; Cen: centromere; Tel: telomere; yellow box (CGI): CpG island; orange boxes (M): CpG sites analyzed by MALDI-TOF MS; green boxes (P): CpG sites analyzed by pyrosequencing. Numbers with diagonal lines indicate CpG units (MALDI-TOF MS) or CpG sites (pyrosequencing), which could not be analyzed. Figures are not drawn to scale. Lower part; Results of MALDI-TOF MS and pyrosequencing are shown. In methylation graphs, the vertical axis represents the methylation index (0: no methylation; 1: full methylation). The horizontal axis represents CpG units or CpG sites. CL7 was analyzed in duplicate by MALDI-TOF MS analysis. Blue and red lines: CL7; green line: CBD1; dark grey line: CL16. [file 1471-2407-13-608-S2.pdf]

Figure S1

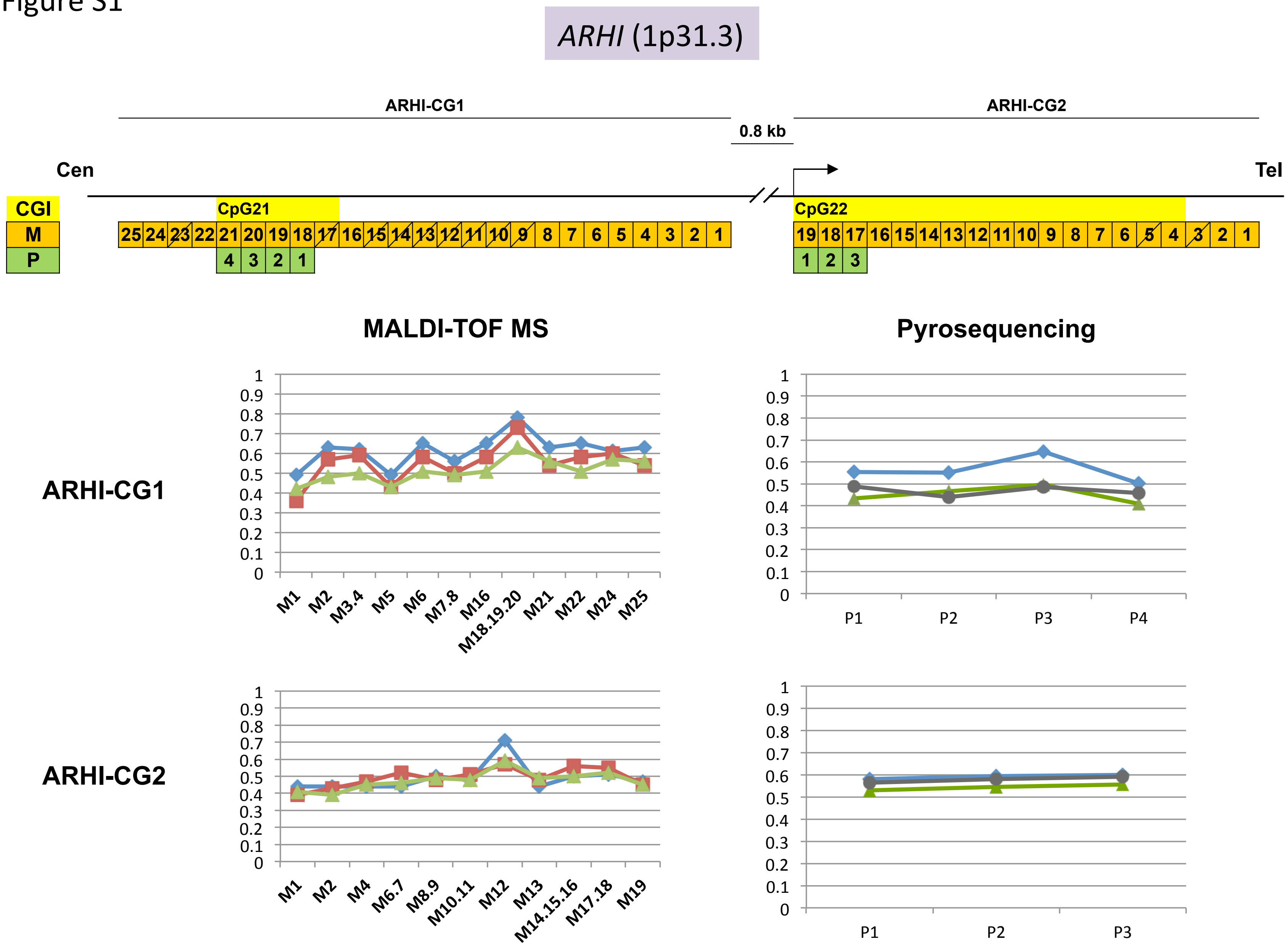

ARHI (1p31.3)
ARHI-CG1
ARHI-CG2
0.8 kb
Cen
Tel
CGI
M
P
CpG21
CpG22
MALDI-TOF MS
Pyrosequencing
ARHI-CG1
ARHI-CG2
M1 M2 M3.4 M5 M6 M7.8 M16 M18.19.20 M21 M22 M24 M25
P1 P2 P3 P4
M1 M2 M4 M6.7 M8.9 M10.11 M12 M13 M14.15.16 M17.18 M19
P1 P2 P3

TP73 (1p36.32)

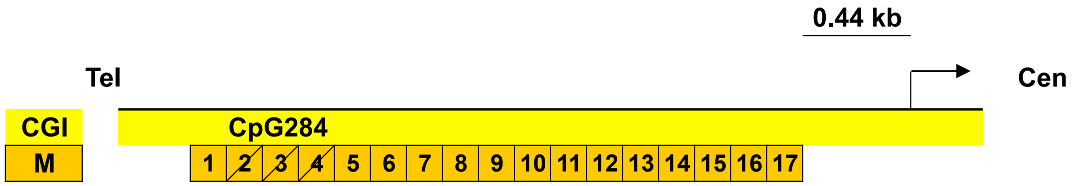

MALDI-TOF MS

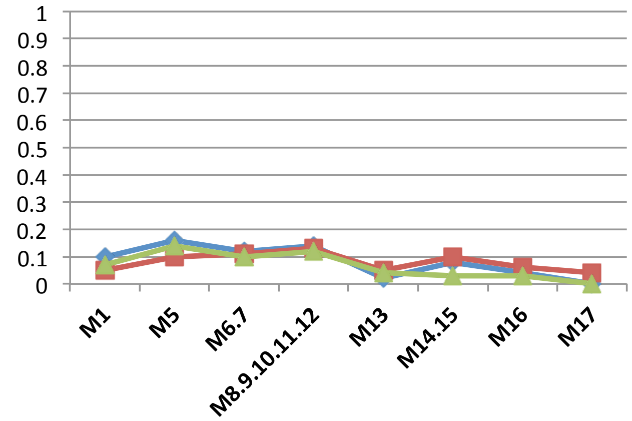

Pyrosequencing

NA

SPTBN1 (2p16.2)

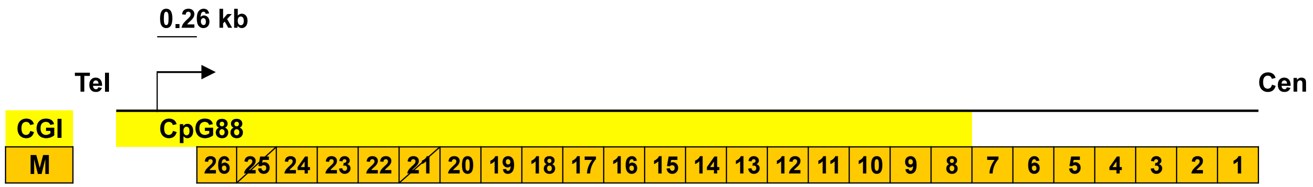

MALDI-TOF MS

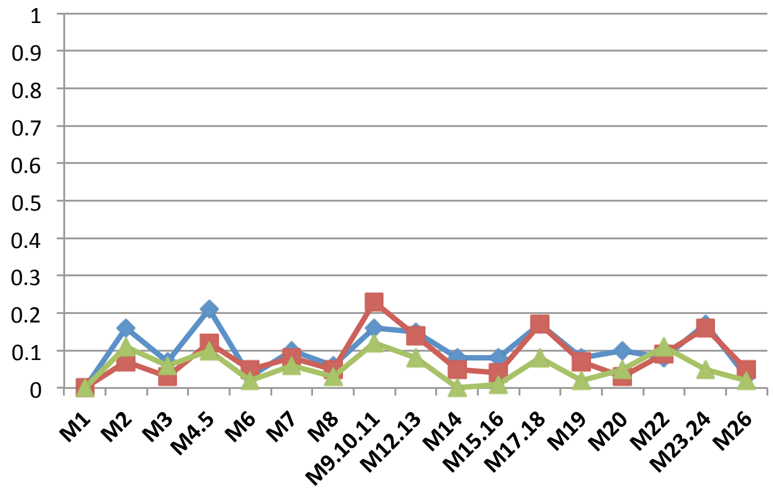

Pyrosequencing

NA

ZDBF2 (2q33.3)

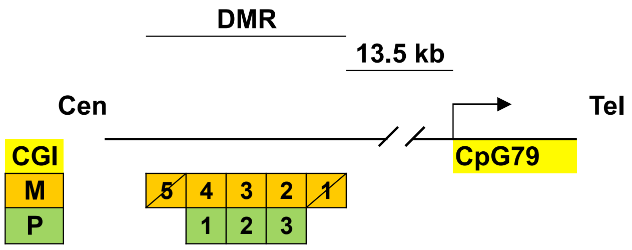
DMR
13.5 kb
Cen
Tel
CGI
M
P
CpG79
5
4
3
2
1
1
2
3

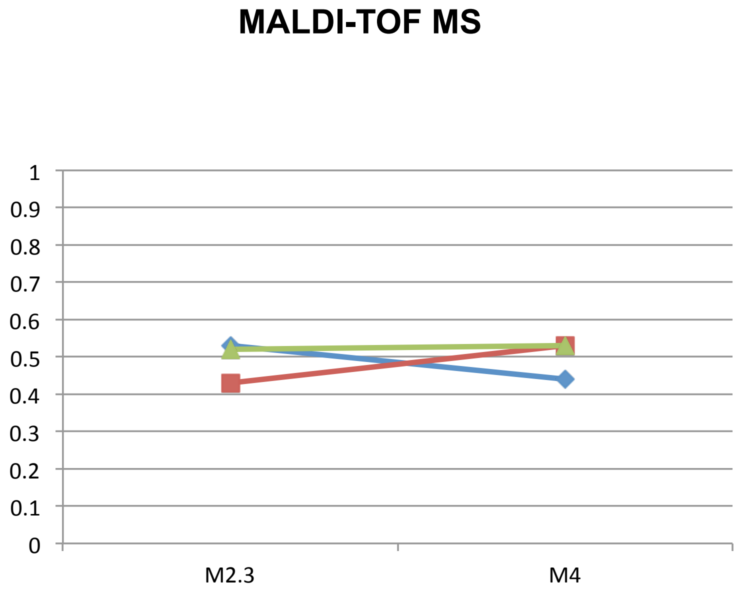
MALDI-TOF MS
1
0.9
0.8
0.7
0.6
0.5
0.4
0.3
0.2
0.1
0
M2.3
M4

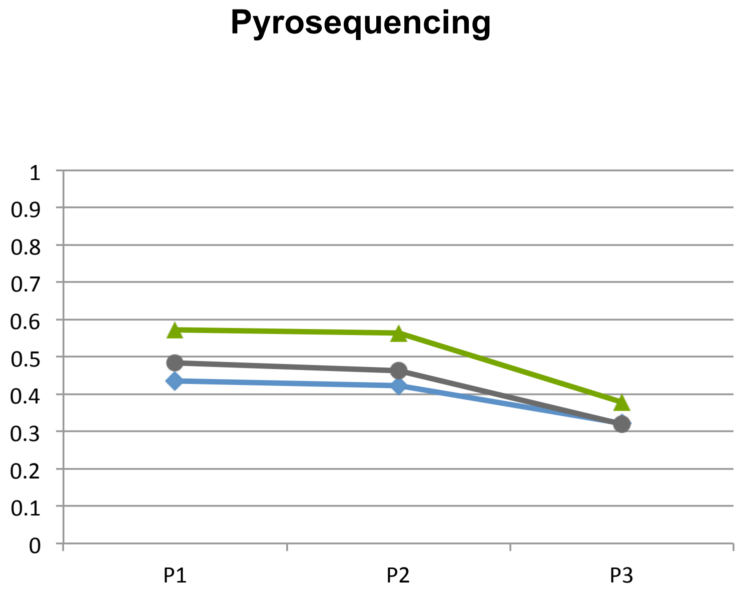
Pyrosequencing
1
0.9
0.8
0.7
0.6
0.5
0.4
0.3
0.2
0.1
0
P1
P2
P3

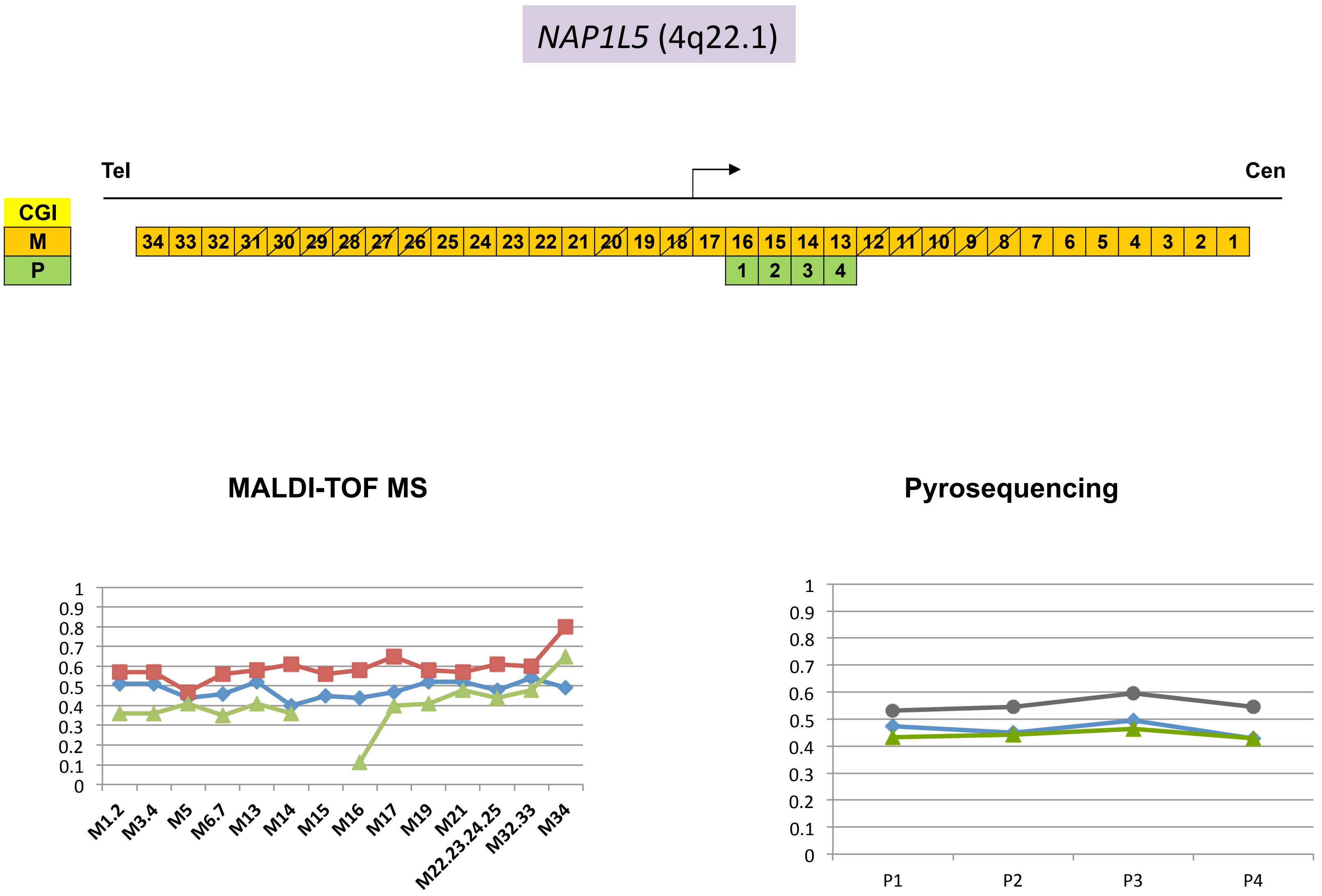

NAP1L5 (4q22.1)
Tel
Cen
CGI
M
P
34 33 32 31 30 29 28 27 26 25 24 23 22 21 20 19 18 17 16 15 14 13 12 11 10 9 8 7 6 5 4 3 2 1
1 2 3 4
MALDI-TOF MS
1 0.9 0.8 0.7 0.6 0.5 0.4 0.3 0.2 0.1 0
M1.2 M3.4 M5 M6.7 M13 M14 M15 M16 M17 M19 M21 M22.23.24.25 M32.33 M34
Pyrosequencing
1 0.9 0.8 0.7 0.6 0.5 0.4 0.3 0.2 0.1 0
P1 P2 P3 P4

ZAC (6q24.2)

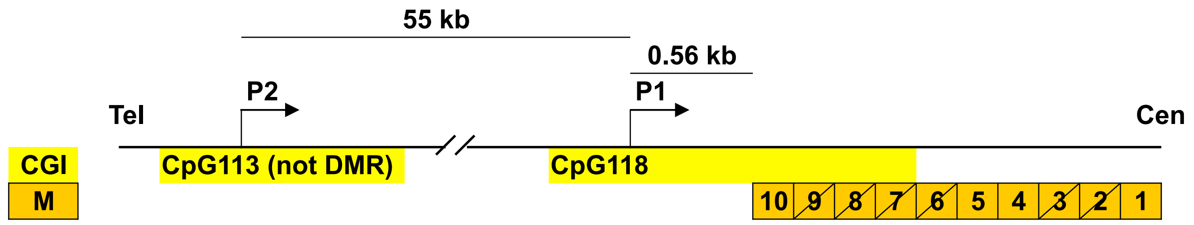

MALDI-TOF MS

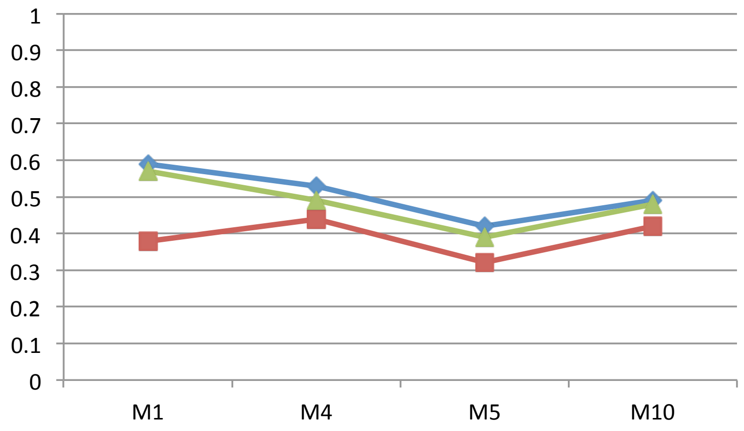

Pyrosequencing

NA

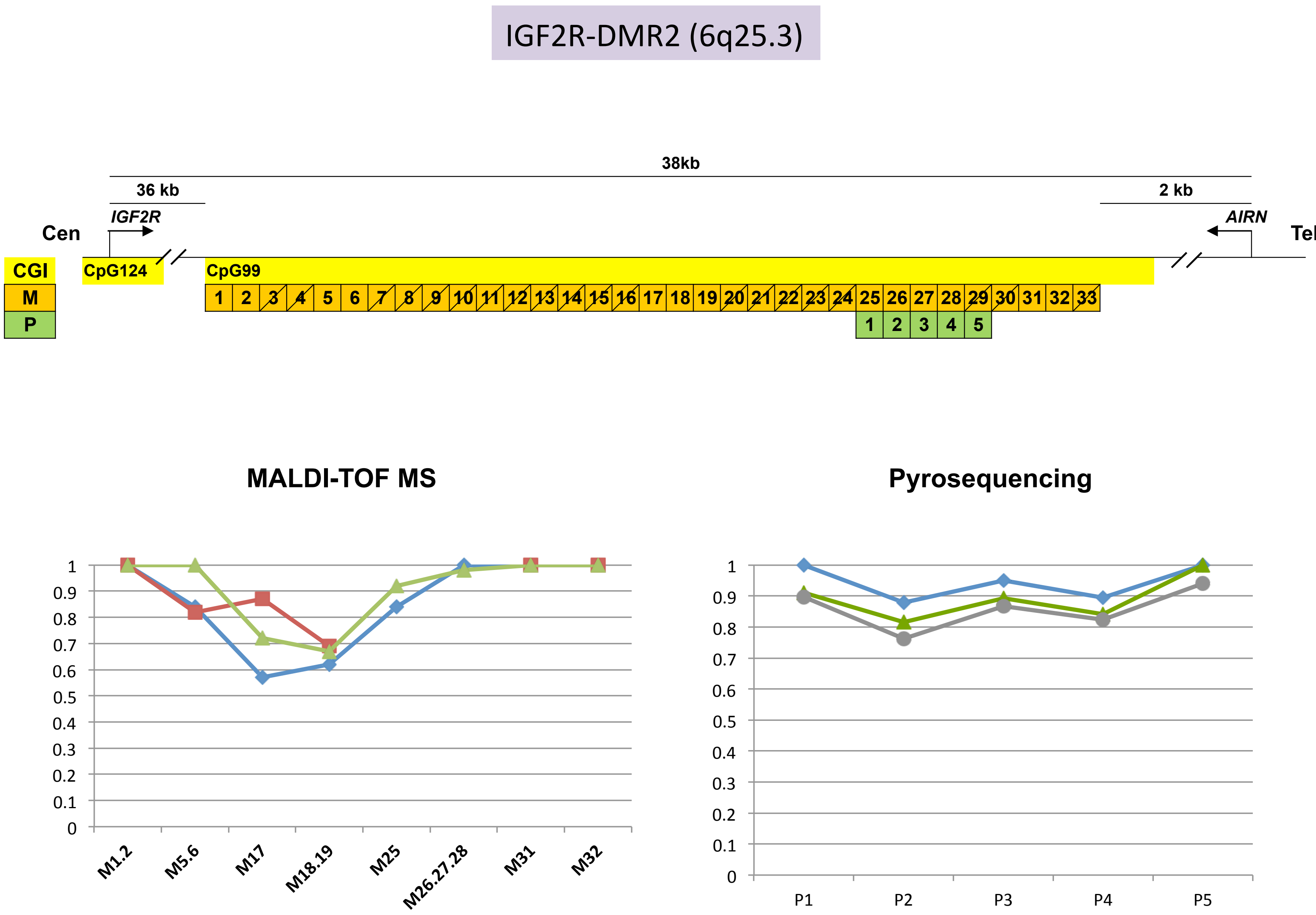
IGF2R-DMR2 (6q25.3)
38kb
36 kb
2 kb
IGF2R
AIRN
Cen
Tel
CGI
M
P
CpG124
CpG99
MALDI-TOF MS
Pyrosequencing
M1.2
M5.6
M17
M18.19
M25
M26.27.28
M31
M32
P1
P2
P3
P4
P5

PEG10 (7q21.3)

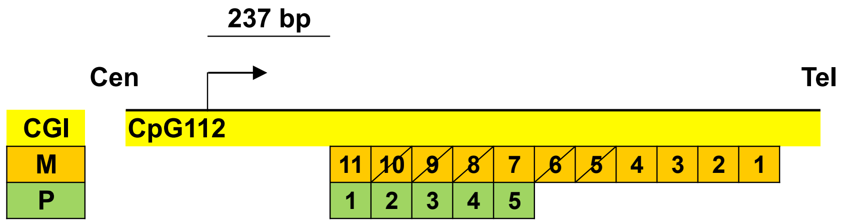

MALDI-TOF MS

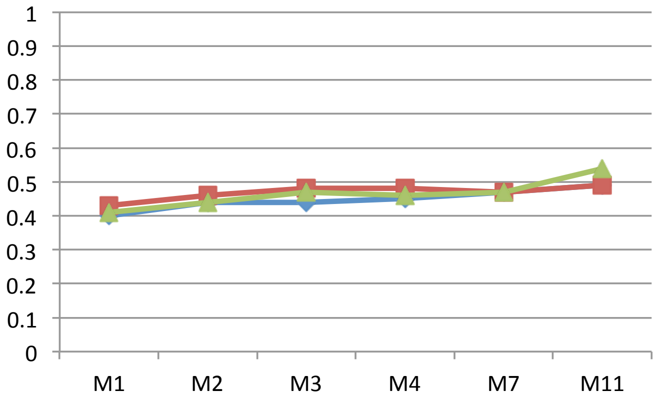

Pyrosequencing

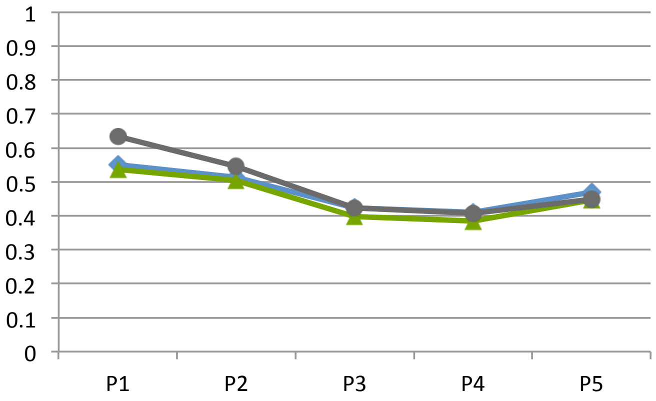

MEST (7q32.2)

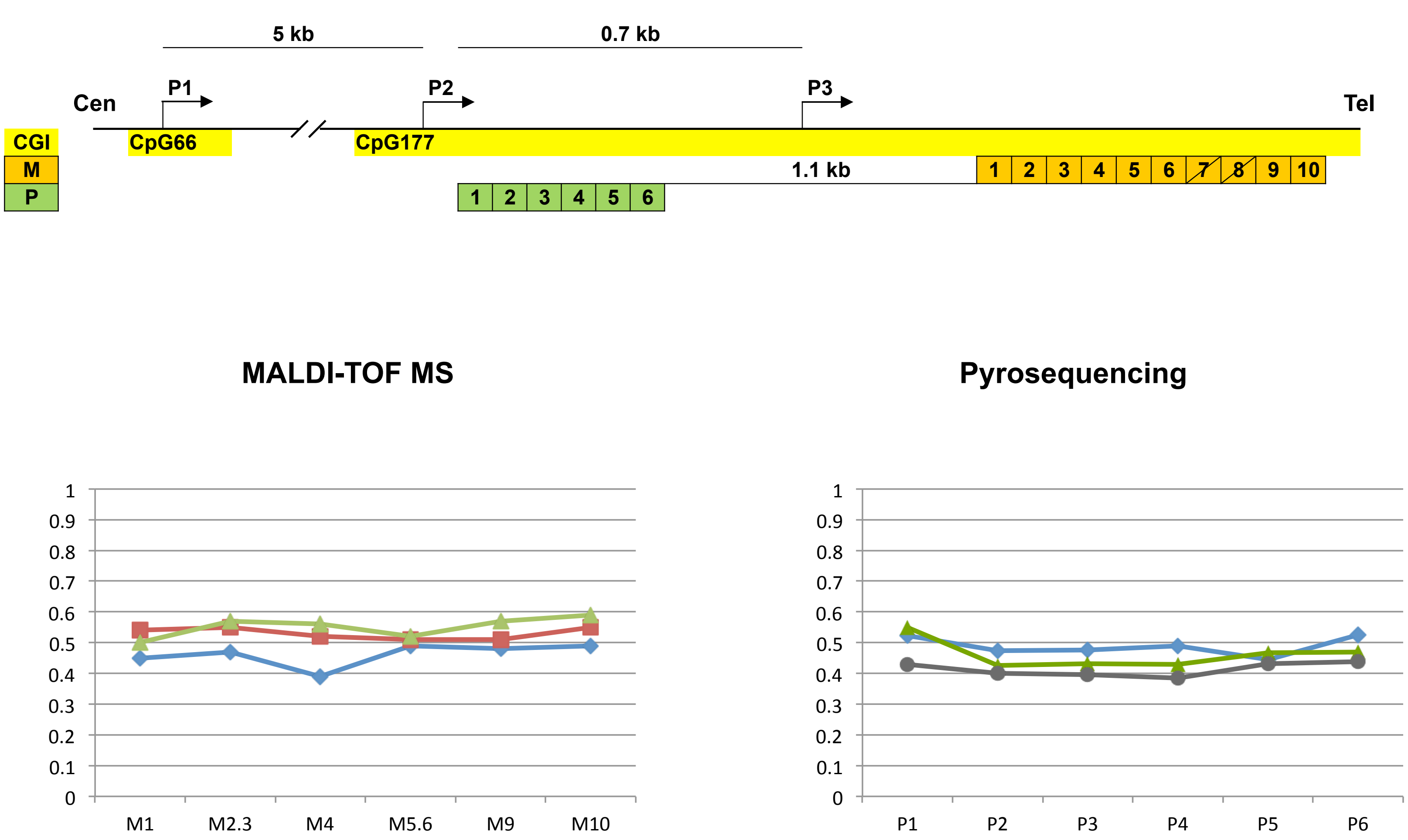
5 kb
0.7 kb
P1
P2
P3
Cen
Tel
CGI
M
P
CpG66
CpG177
1.1 kb
1 2 3 4 5 6 7 8 9 10
1 2 3 4 5 6
MALDI-TOF MS
Pyrosequencing
1 0.9 0.8 0.7 0.6 0.5 0.4 0.3 0.2 0.1 0
M1 M2.3 M4 M5.6 M9 M10
P1 P2 P3 P4 P5 P6

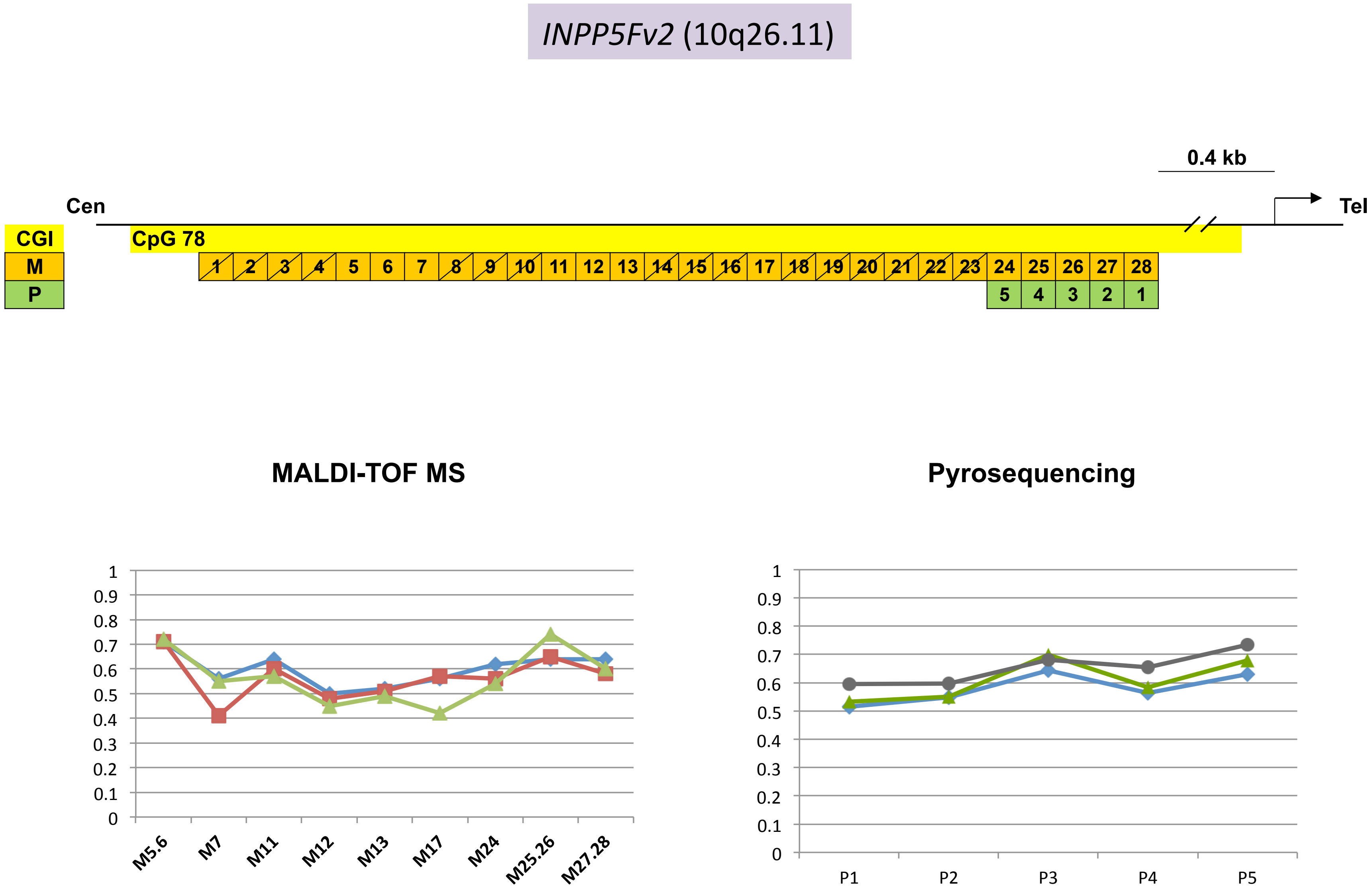
INPP5Fv2 (10q26.11)
0.4 kb
Cen
Tel
CGI
M
P
CpG 78
1 2 3 4 5 6 7 8 9 10 11 12 13 14 15 16 17 18 19 20 21 22 23 24 25 26 27 28
5 4 3 2 1
MALDI-TOF MS
M5.6 M7 M11 M12 M13 M17 M24 M25.26 M27.28
Pyrosequencing
P1 P2 P3 P4 P5

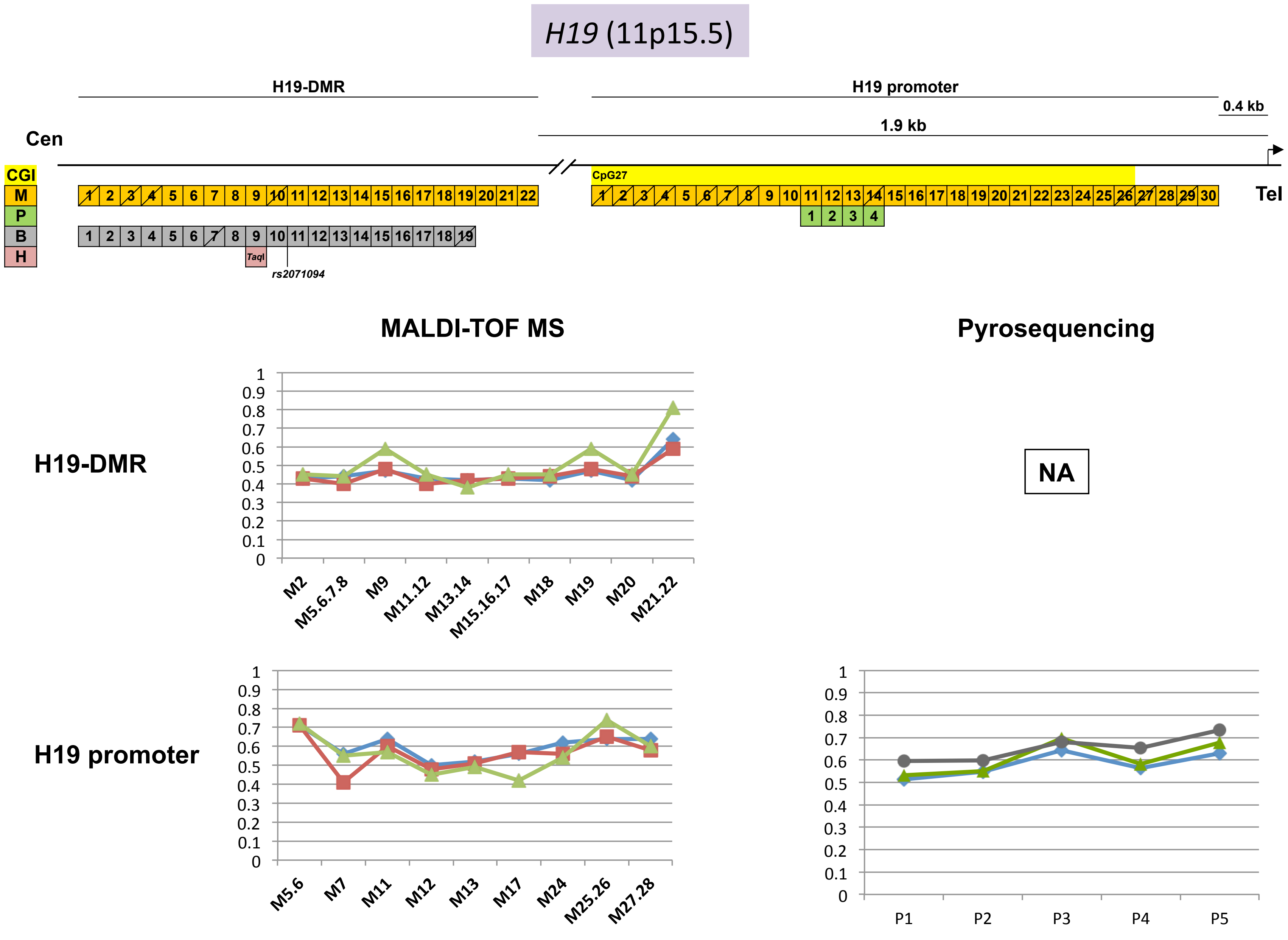
H19 (11p15.5)
H19-DMR
H19 promoter
0.4 kb
1.9 kb
Cen
Tel
CGI
M
P
B
H
CpG27
TaqI
rs2071094
MALDI-TOF MS
Pyrosequencing
H19-DMR
H19 promoter
NA
M2
M5.6.7.8
M9
M11.12
M13.14
M15.16.17
M18
M19
M20
M21.22
M5.6
M7
M11
M12
M13
M17
M24
M25.26
M27.28
P1
P2
P3
P4
P5

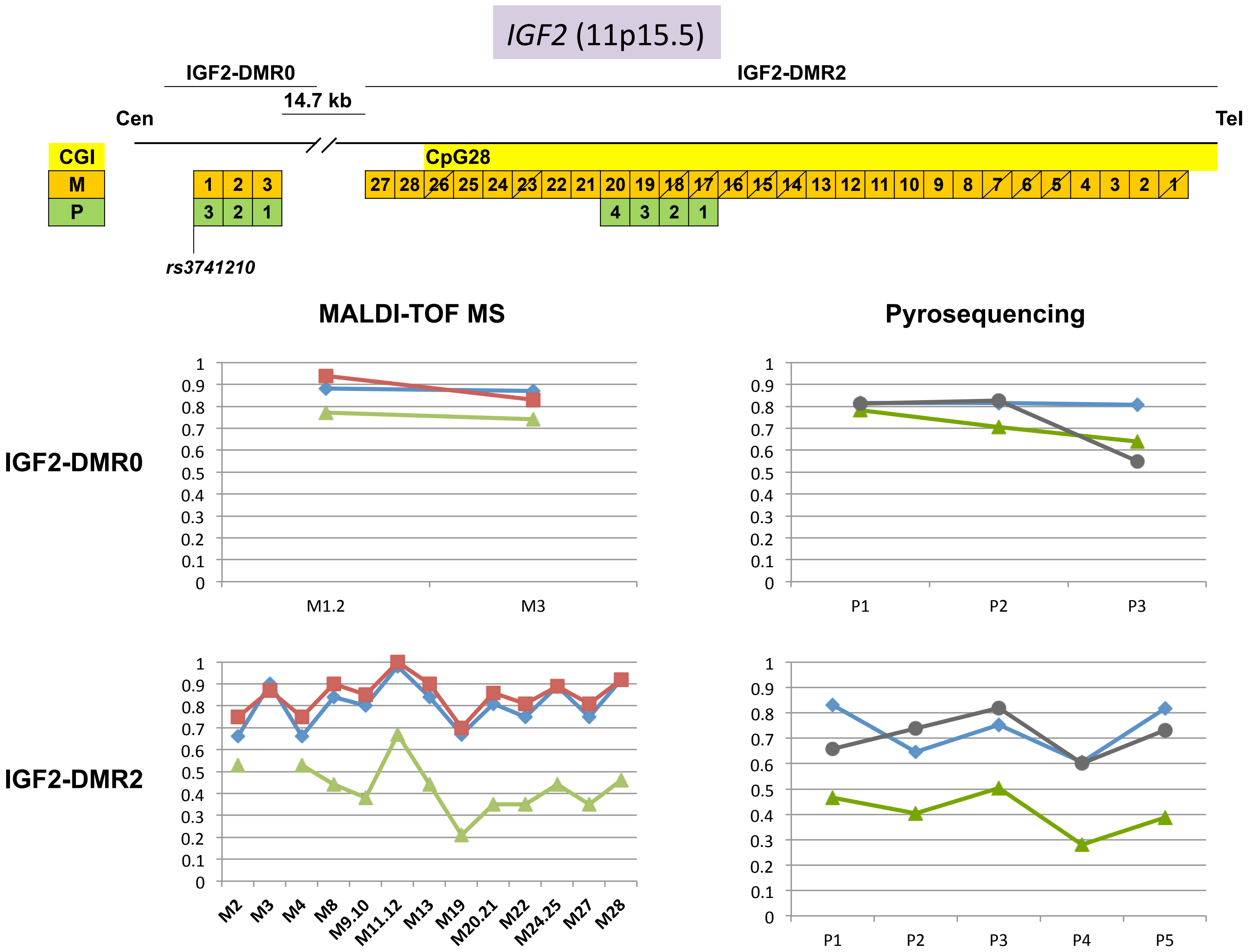

IGF2 (11p15.5)
IGF2-DMR0
IGF2-DMR2
14.7 kb
Cen
Tel
CGI
M
P
CpG28
1 2 3
3 2 1
27 28 26 25 24 23 22 21 20 19 18 17 16 15 14 13 12 11 10 9 8 7 6 5 4 3 2 1
4 3 2 1
rs3741210
MALDI-TOF MS
Pyrosequencing
IGF2-DMR0
IGF2-DMR2
M1.2
M3
P1
P2
P3
M2 M3 M4 M8 M9.10 M11.12 M13 M19 M20.21 M22 M24.25 M27 M28
P1 P2 P3 P4 P5

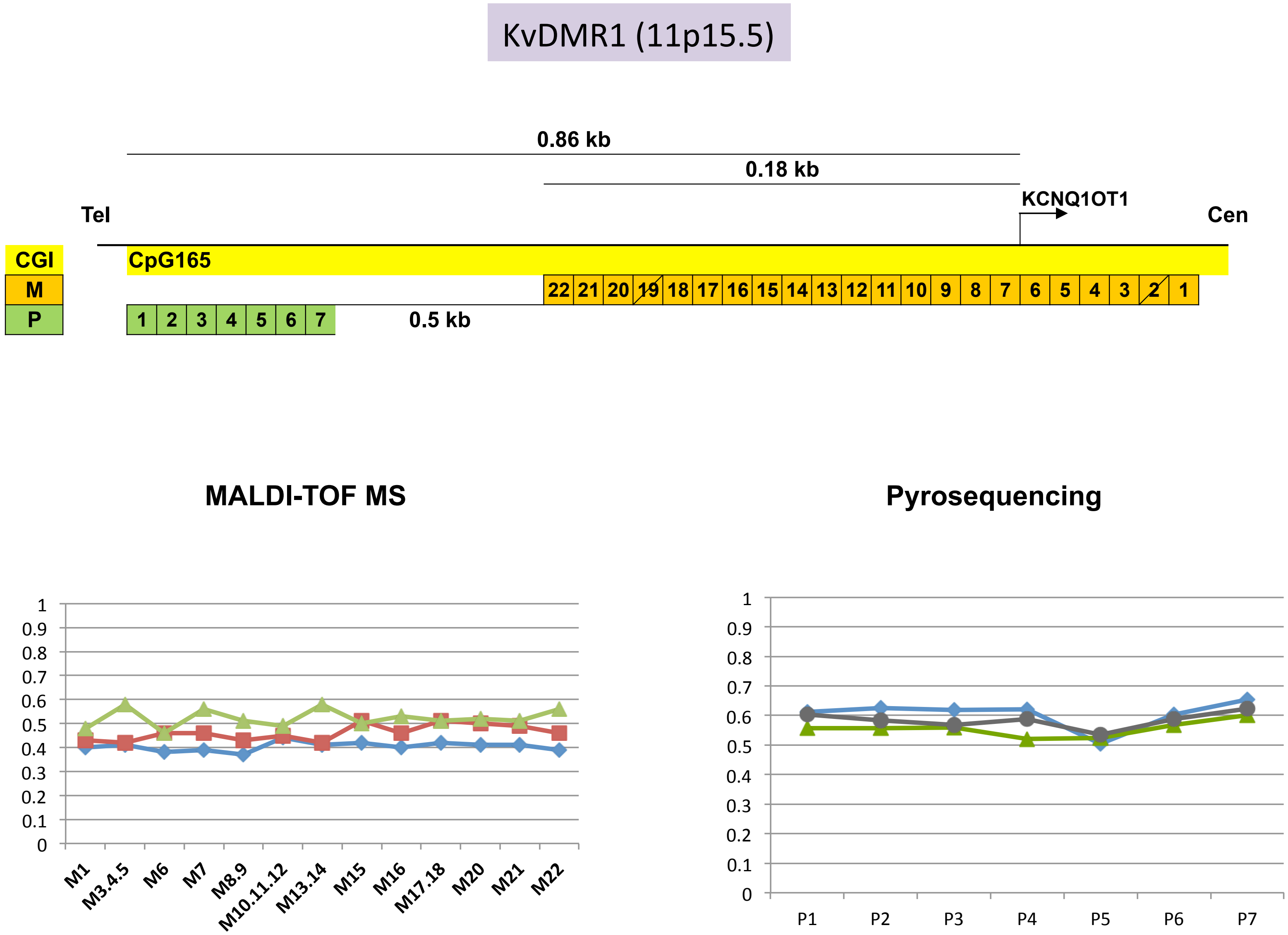

KvDMR1 (11p15.5)
0.86 kb
0.18 kb
KCNQ1OT1
Tel
Cen
CGI
CpG165
M
22 21 20 19 18 17 16 15 14 13 12 11 10 9 8 7 6 5 4 3 2 1
P
1 2 3 4 5 6 7
0.5 kb
MALDI-TOF MS
Pyrosequencing
1 0.9 0.8 0.7 0.6 0.5 0.4 0.3 0.2 0.1 0
M1 M3.4.5 M6 M7 M8.9 M10.11.12 M13.14 M15 M16 M17.18 M20 M21 M22
P1 P2 P3 P4 P5 P6 P7

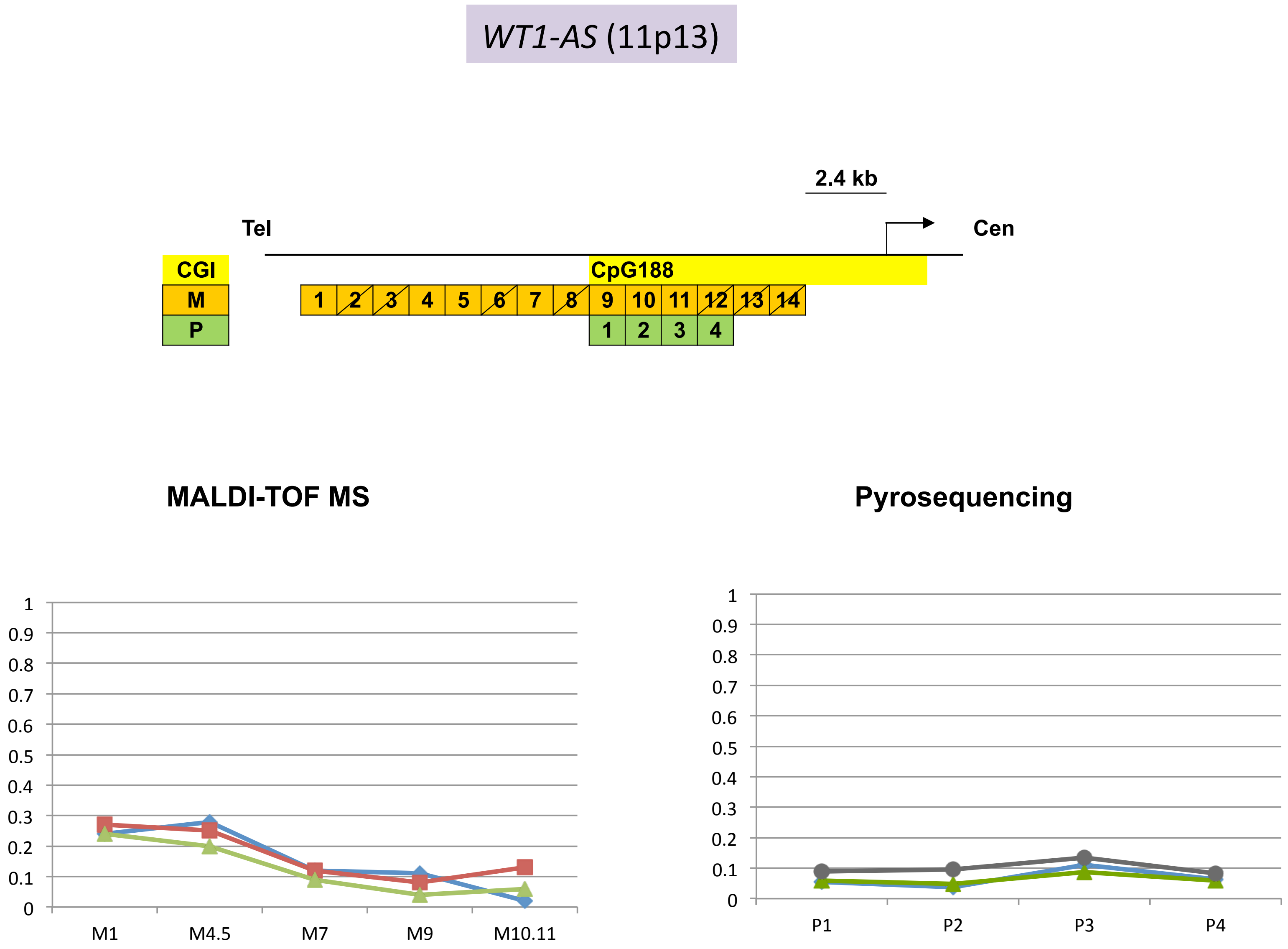
WT1-AS (11p13)
2.4 kb
Tel
Cen
CGI
M
P
CpG188
1 2 3 4 5 6 7 8 9 10 11 12 13 14
1 2 3 4
MALDI-TOF MS
Pyrosequencing
1
0.9
0.8
0.7
0.6
0.5
0.4
0.3
0.2
0.1
0
M1
M4.5
M7
M9
M10.11
P1
P2
P3
P4

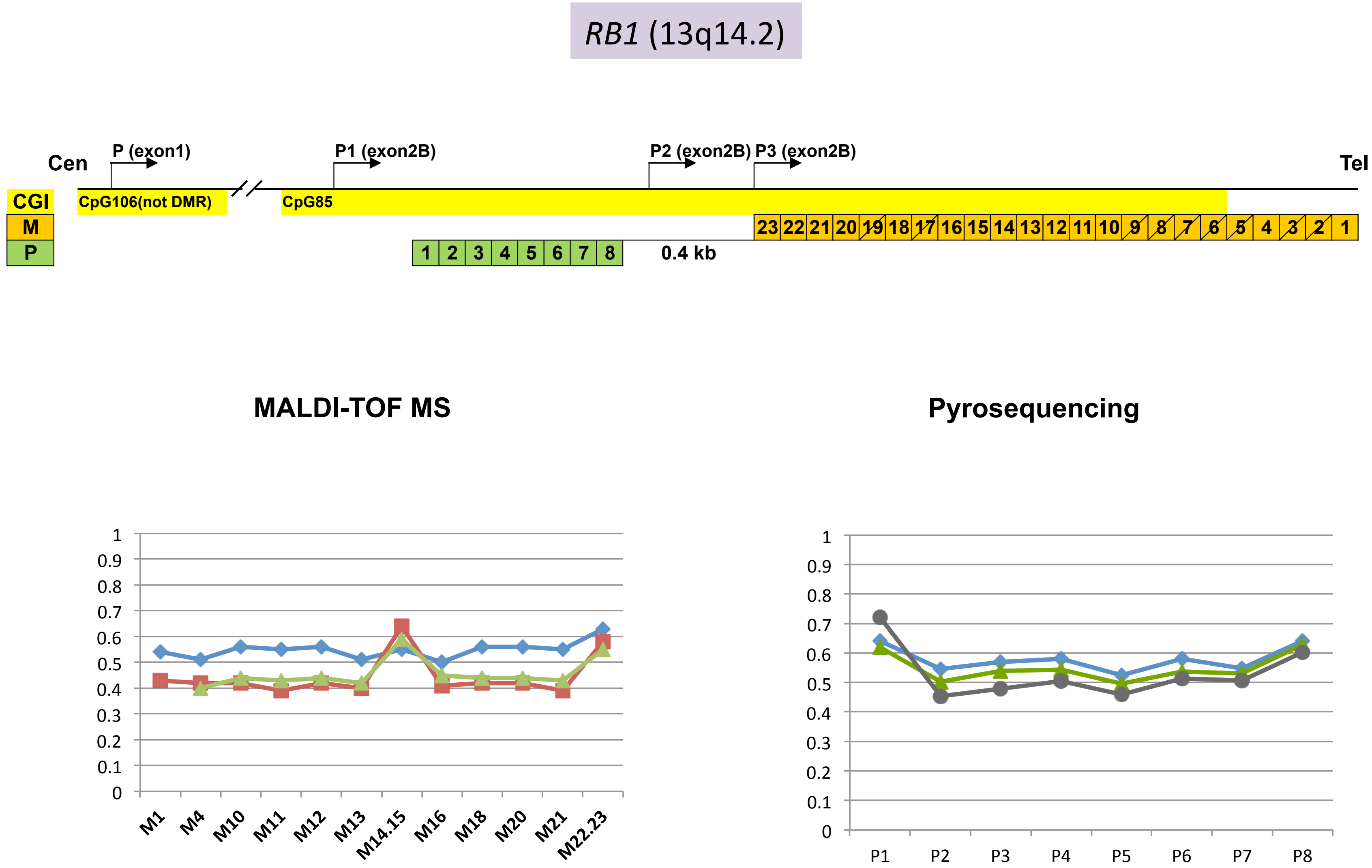
RB1 (13q14.2)
Cen
Tel
P (exon1)
P1 (exon2B)
P2 (exon2B)
P3 (exon2B)
CGI
M
P
CpG106(not DMR)
CpG85
23 22 21 20 19 18 17 16 15 14 13 12 11 10 9 8 7 6 5 4 3 2 1
1 2 3 4 5 6 7 8
0.4 kb
MALDI-TOF MS
Pyrosequencing
1
0.9
0.8
0.7
0.6
0.5
0.4
0.3
0.2
0.1
0
M1
M4
M10
M11
M12
M13
M14.15
M16
M18
M20
M21
M22.23
P1
P2
P3
P4
P5
P6
P7
P8

DLK1 (14q32.2)

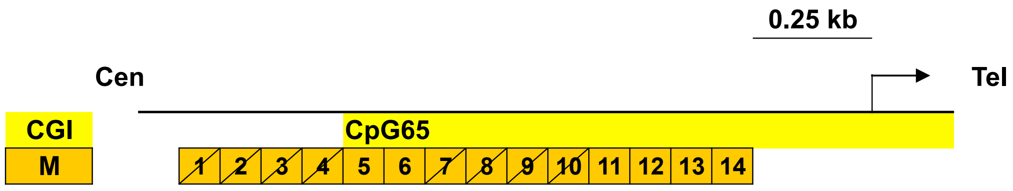

MALDI-TOF MS

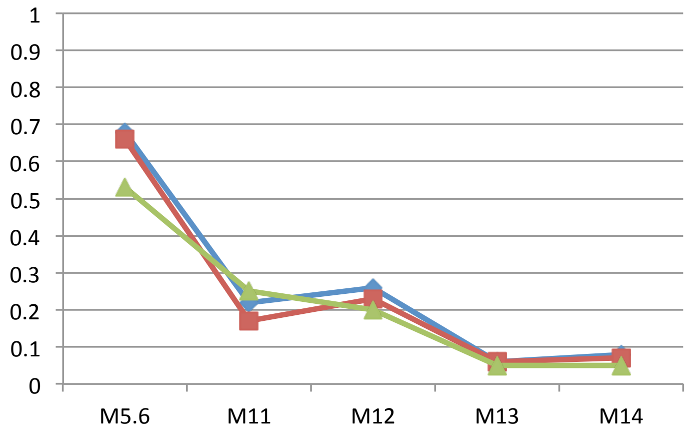

Pyrosequencing

NA

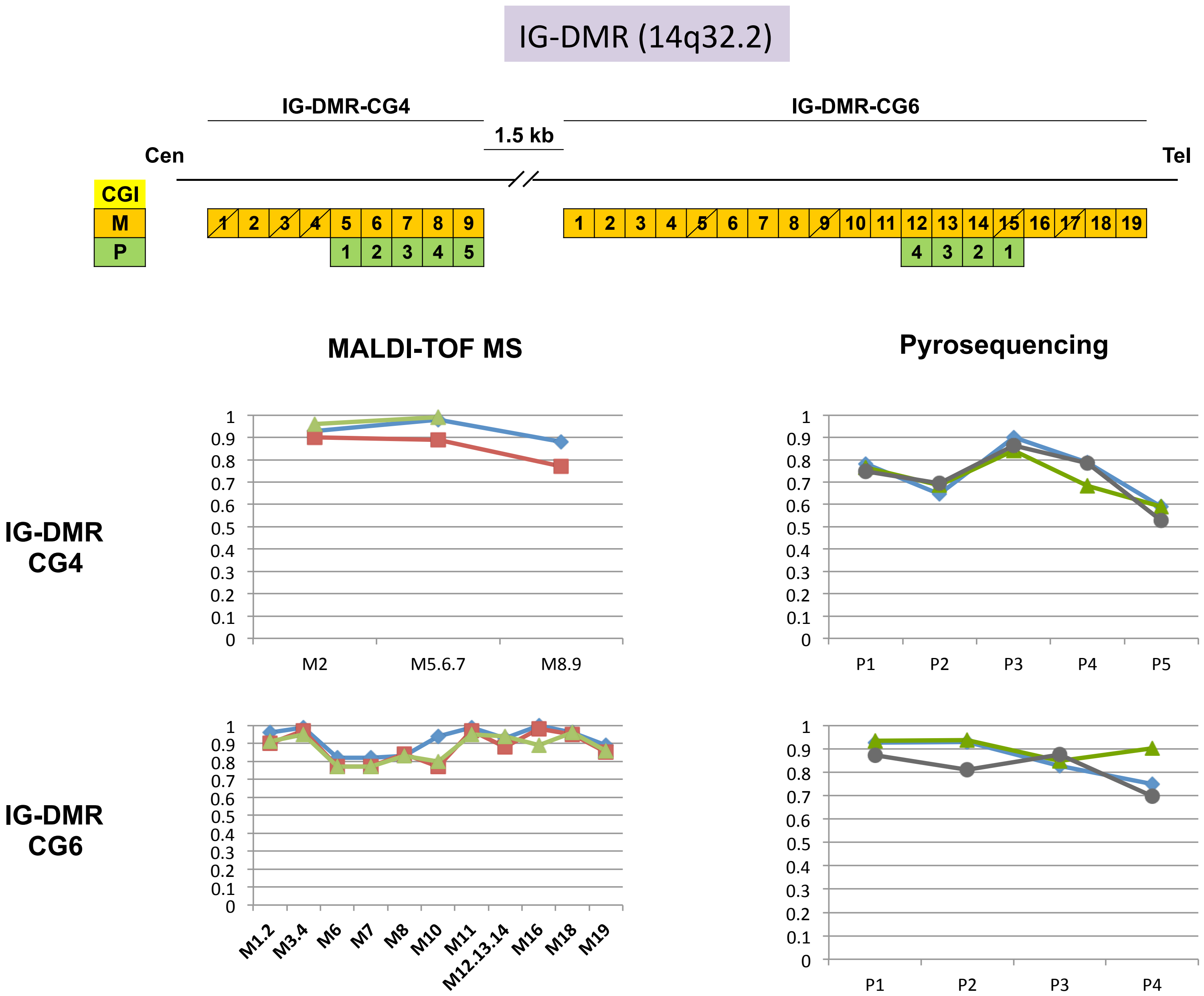

IG-DMR (14q32.2)
IG-DMR-CG4
IG-DMR-CG6
1.5 kb
Cen
Tel
CGI
M
P
MALDI-TOF MS
Pyrosequencing
IG-DMR CG4
IG-DMR CG6
M2
M5.6.7
M8.9
P1
P2
P3
P4
P5
M1.2
M3.4
M6
M7
M8
M10
M11
M12.13.14
M16
M18
M19

MEG3-CG7 (14q32.2)

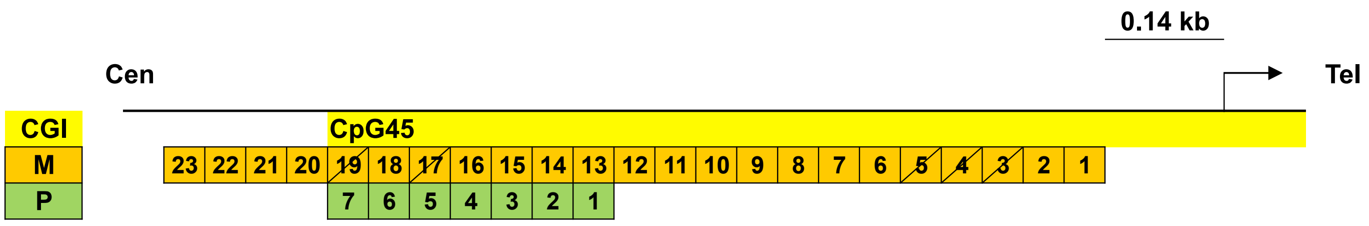

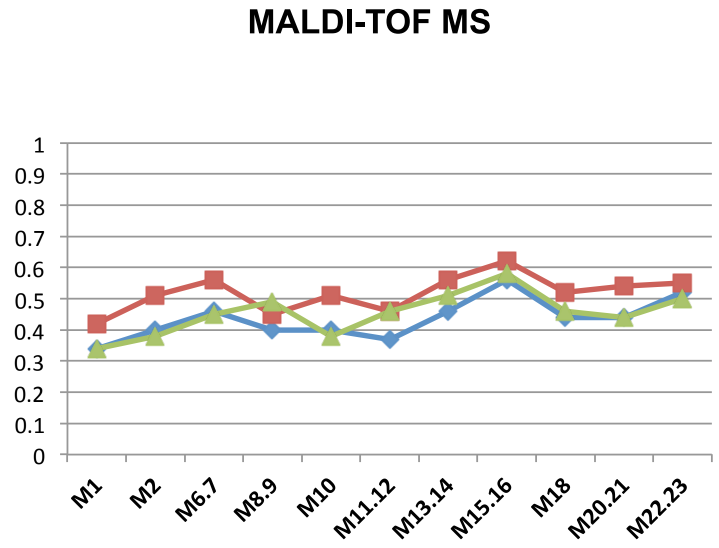

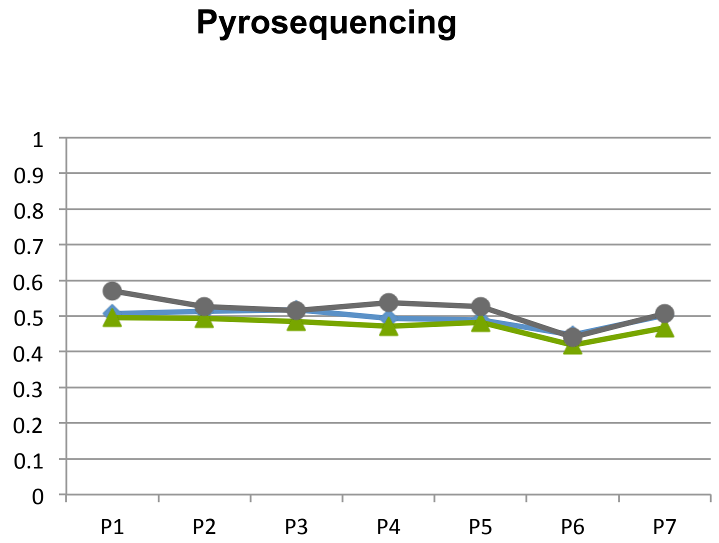

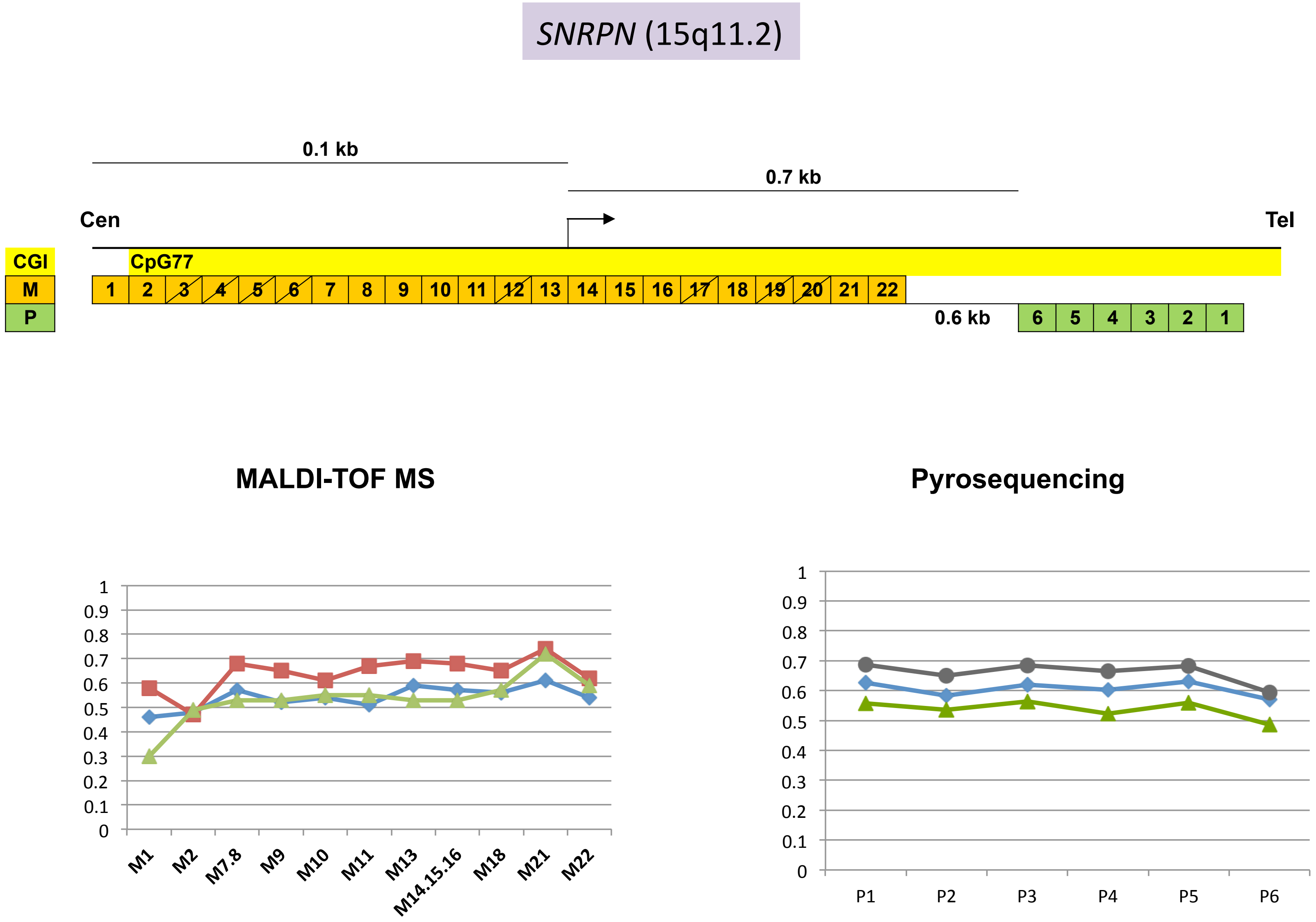
SNRPN (15q11.2)
0.1 kb
0.7 kb
Cen
Tel
CGI
CpG77
M
1 2 3 4 5 6 7 8 9 10 11 12 13 14 15 16 17 18 19 20 21 22
P
0.6 kb
6 5 4 3 2 1
MALDI-TOF MS
Pyrosequencing
M1 M2 M7.8 M9 M10 M11 M13 M14.15.16 M18 M21 M22
P1 P2 P3 P4 P5 P6
0 0.1 0.2 0.3 0.4 0.5 0.6 0.7 0.8 0.9 1

TCEB3C (18q21.1)

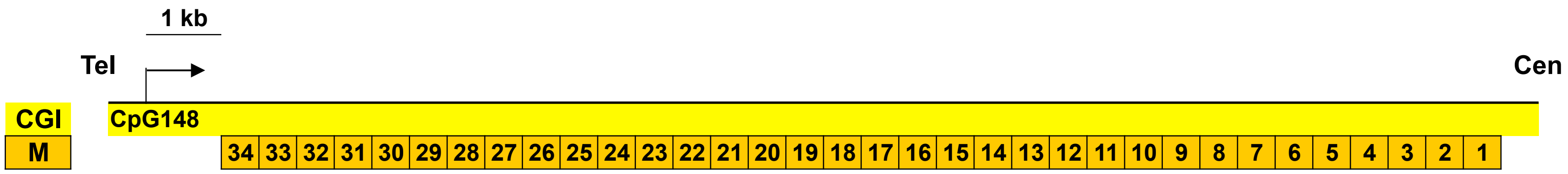

MALDI-TOF MS

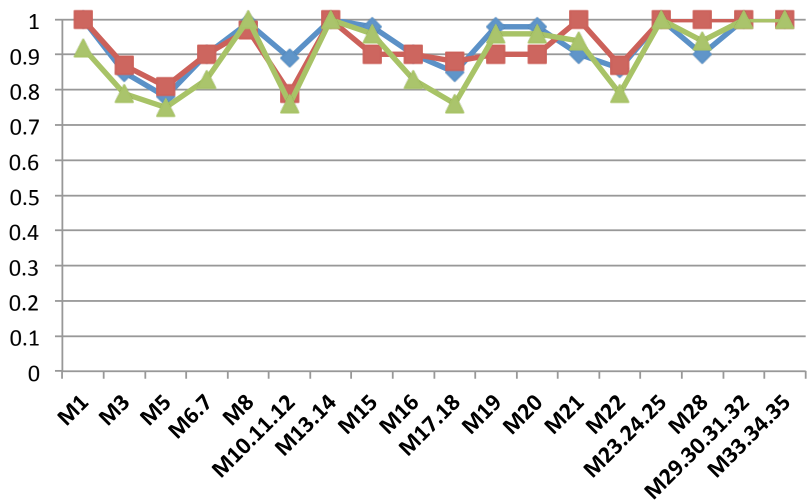

Pyrosequencing

NA

PEG3 (19q13.43)

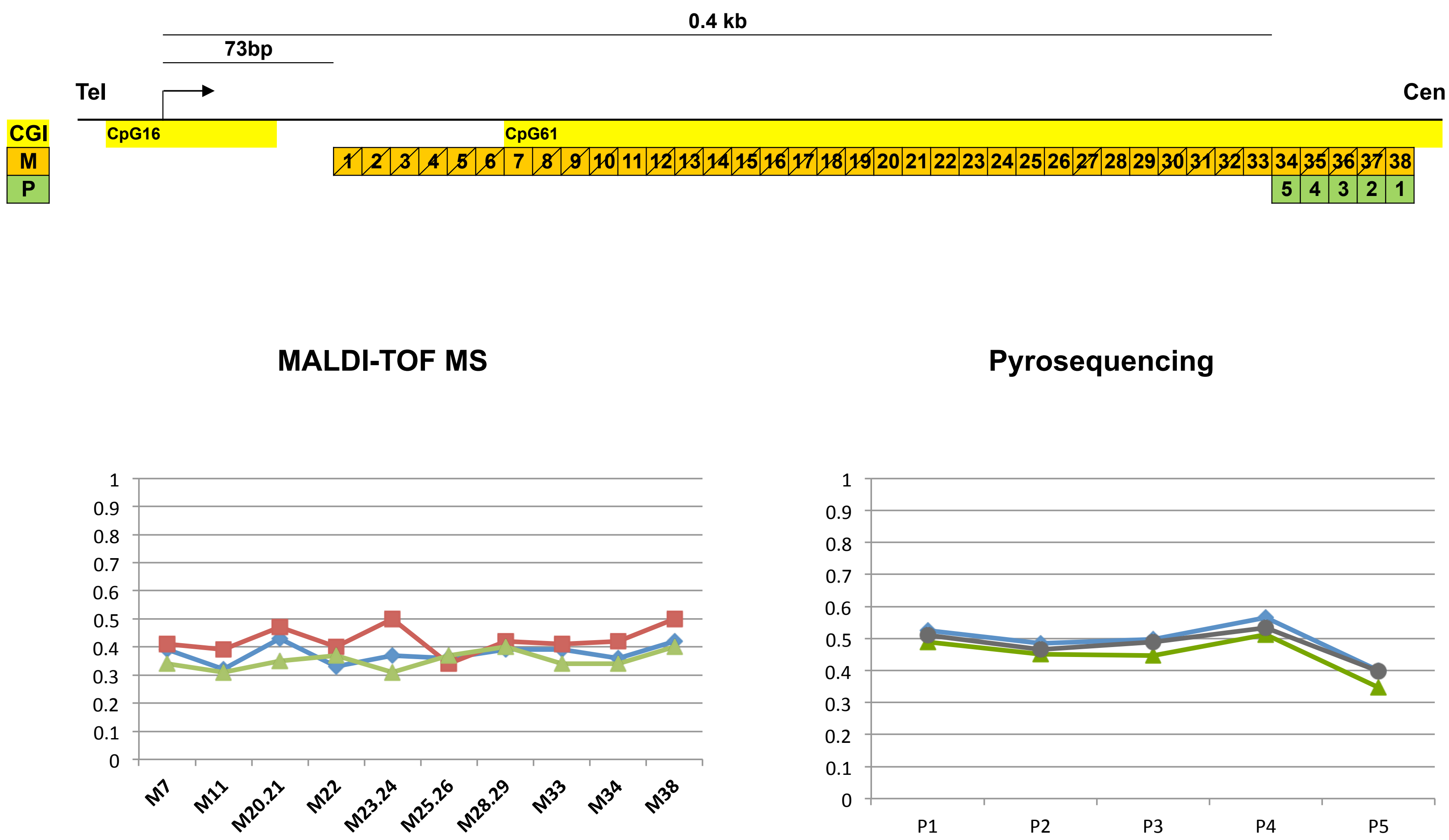

USP29 (19q13.43)

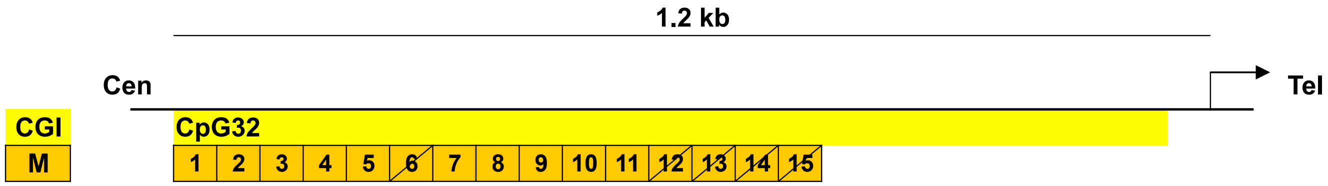

MALDI-TOF MS

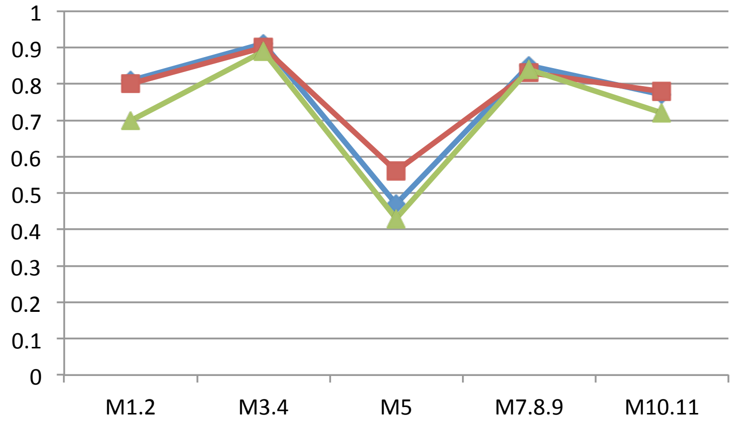

Pyrosequencing

NA

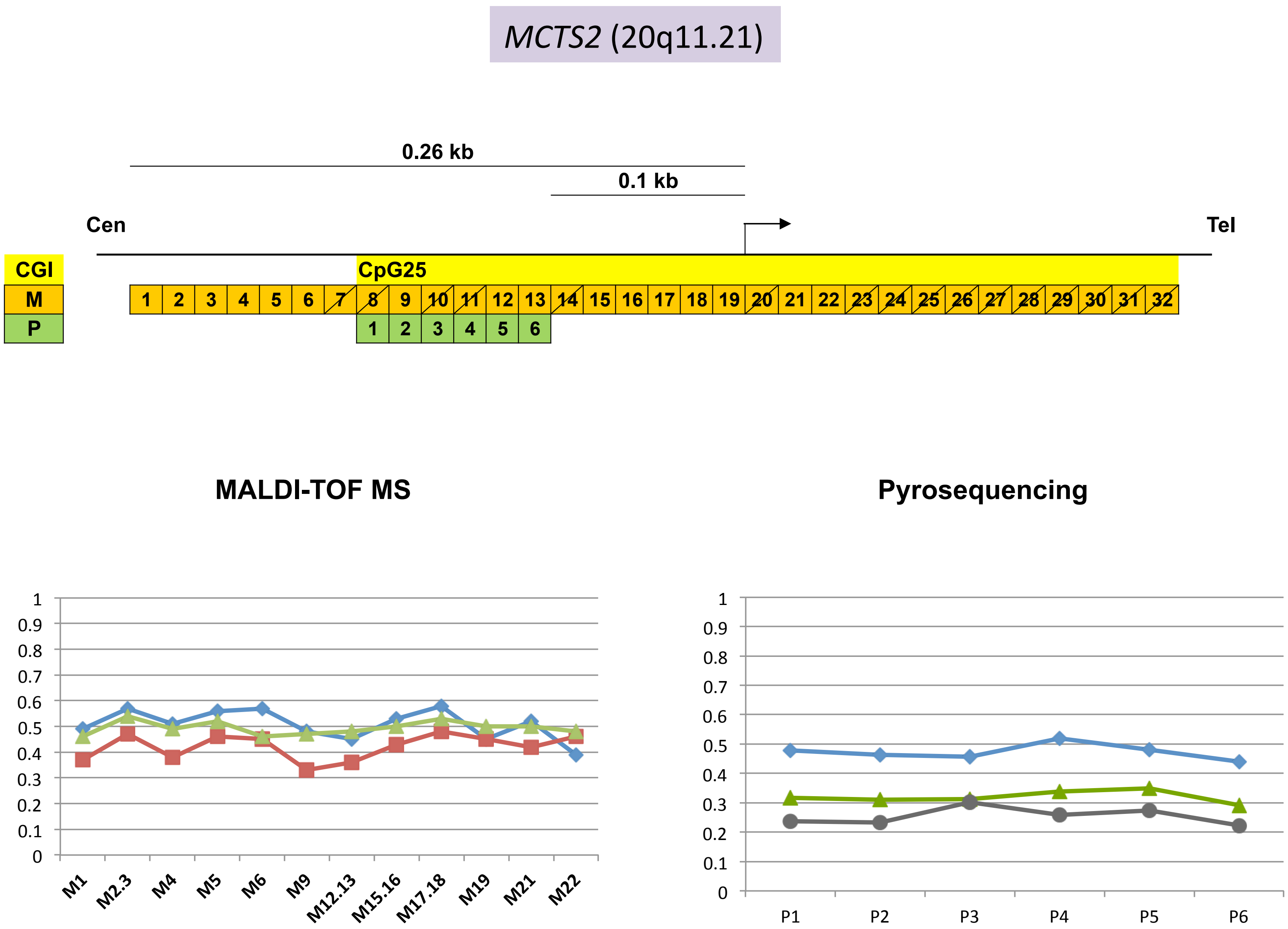

MCTS2 (20q11.21)
0.26 kb
0.1 kb
Cen
Tel
CGI
CpG25
M
1 2 3 4 5 6 7 8 9 10 11 12 13 14 15 16 17 18 19 20 21 22 23 24 25 26 27 28 29 30 31 32
P
1 2 3 4 5 6
MALDI-TOF MS
1 0.9 0.8 0.7 0.6 0.5 0.4 0.3 0.2 0.1 0
M1 M2.3 M4 M5 M6 M9 M12.13 M15.16 M17.18 M19 M21 M22
Pyrosequencing
1 0.9 0.8 0.7 0.6 0.5 0.4 0.3 0.2 0.1 0
P1 P2 P3 P4 P5 P6

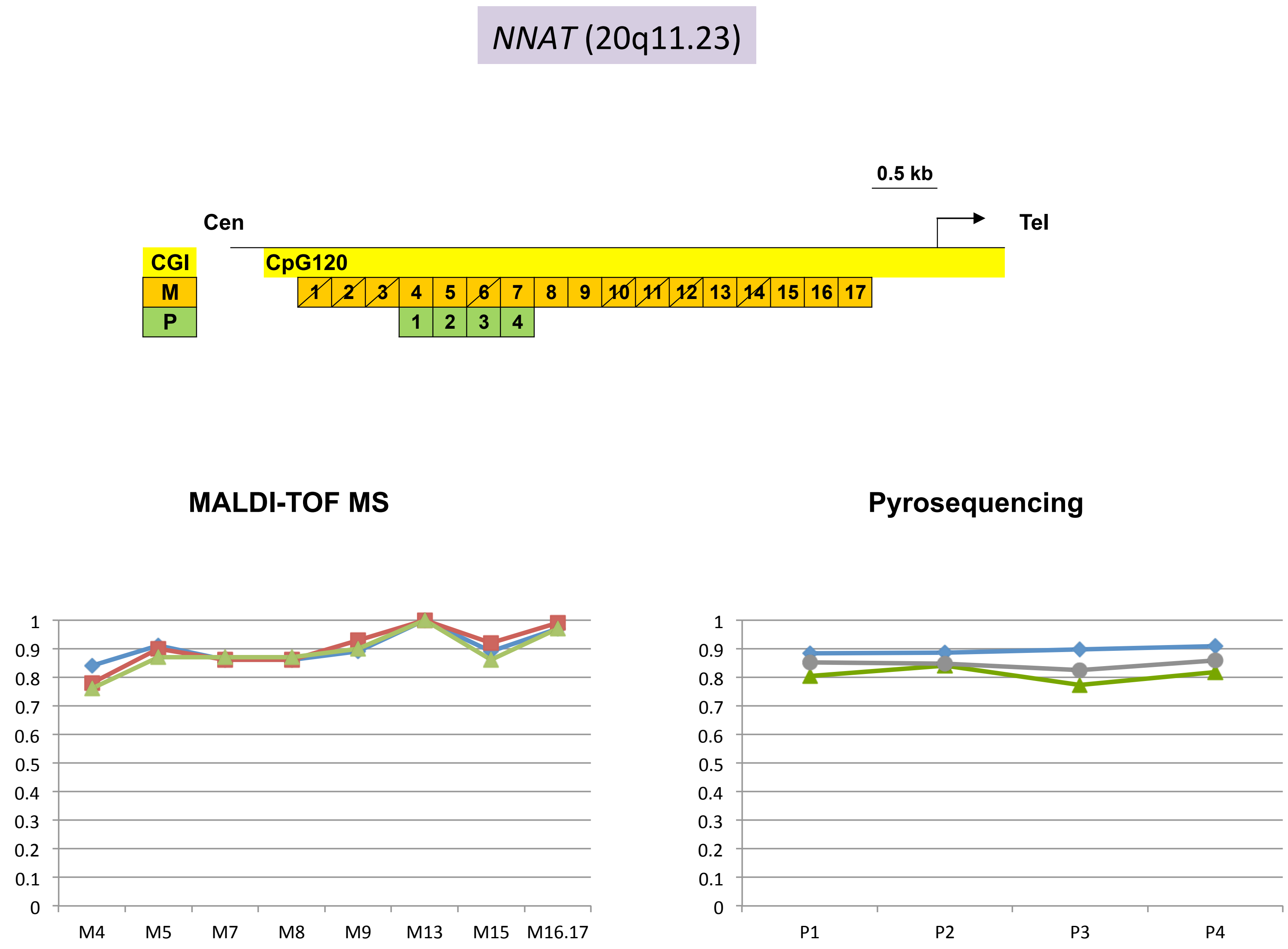

NNAT (20q11.23)
0.5 kb
Cen
Tel
CGI
CpG120
M
1 2 3 4 5 6 7 8 9 10 11 12 13 14 15 16 17
P
1 2 3 4
MALDI-TOF MS
Pyrosequencing
1
0.9
0.8
0.7
0.6
0.5
0.4
0.3
0.2
0.1
0
M4 M5 M7 M8 M9 M13 M15 M16.17
P1 P2 P3 P4

L3MBTL (20q13.12)

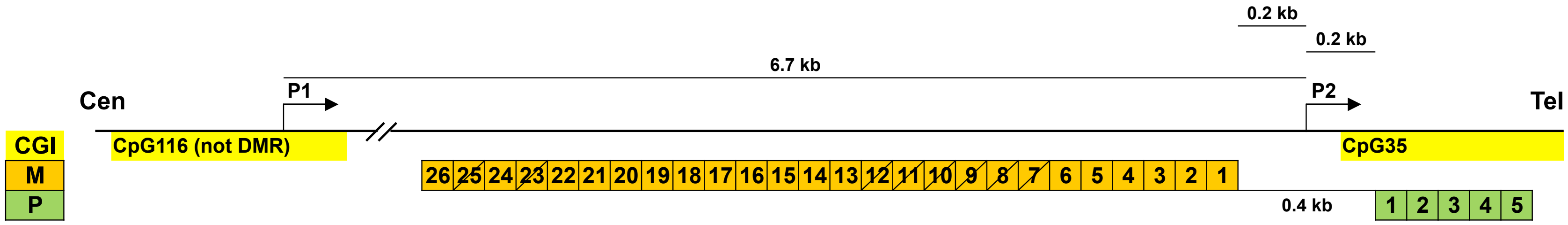

MALDI-TOF MS

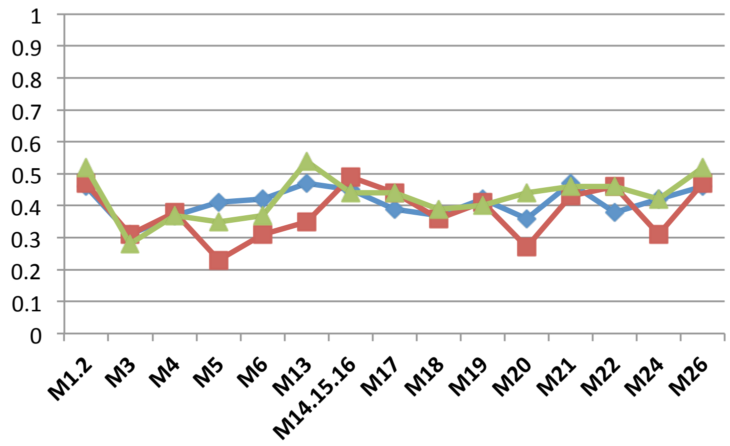

Pyrosequencing

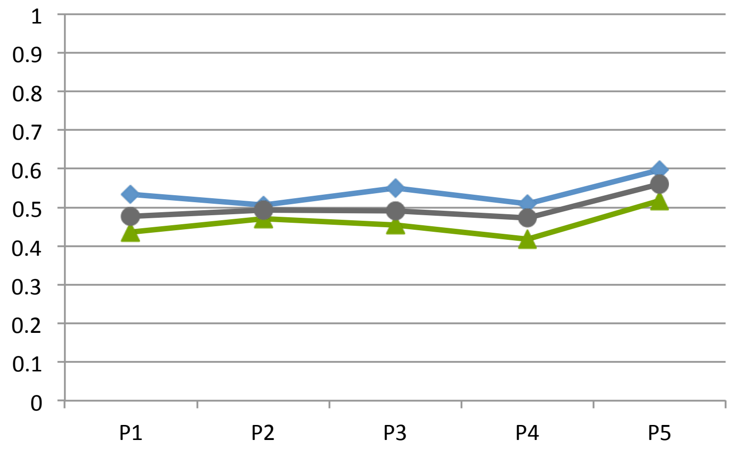

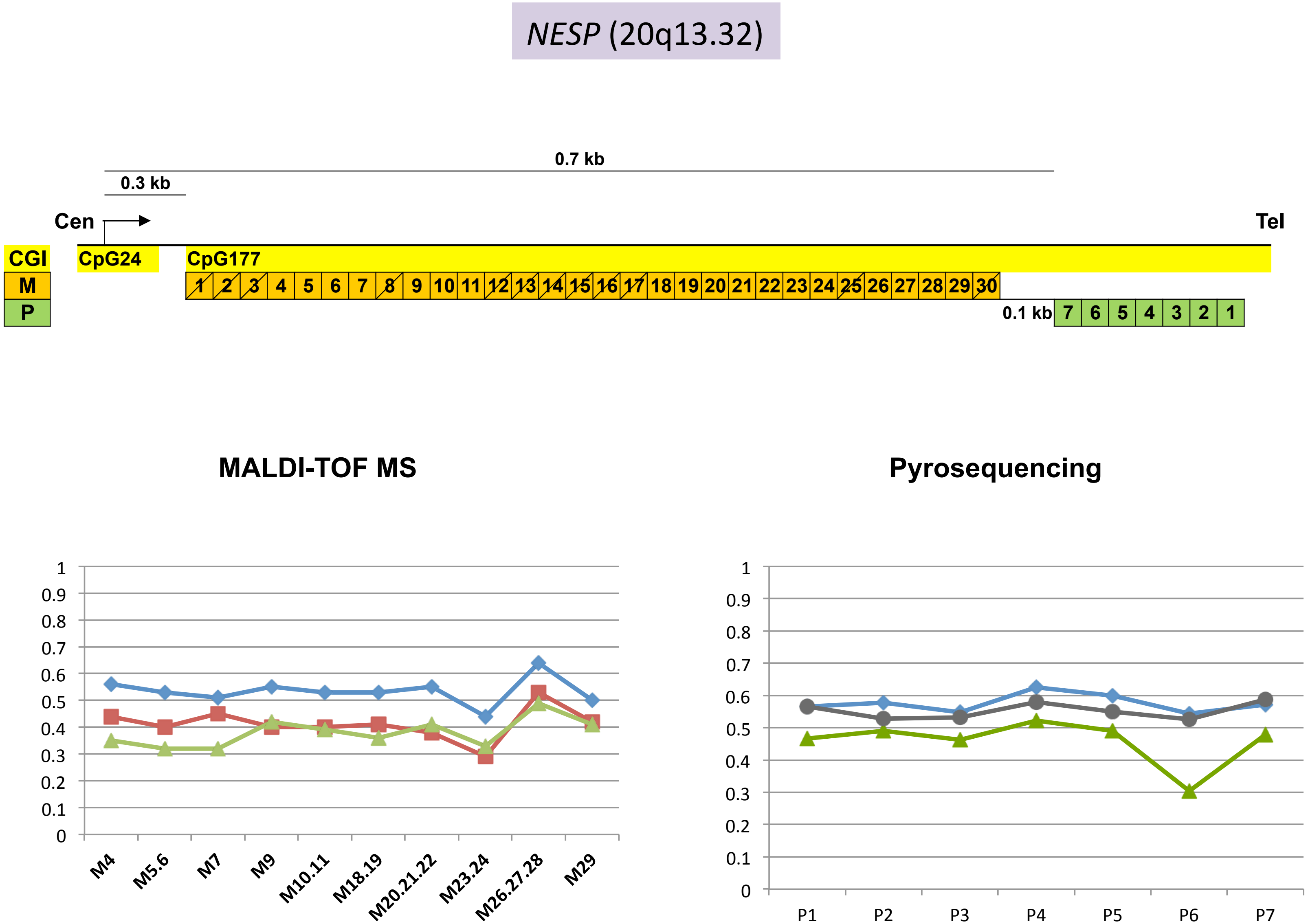

NESP (20q13.32)
0.7 kb
0.3 kb
Cen
Tel
CGI
M
P
CpG24
CpG177
1 2 3 4 5 6 7 8 9 10 11 12 13 14 15 16 17 18 19 20 21 22 23 24 25 26 27 28 29 30
0.1 kb
7 6 5 4 3 2 1
MALDI-TOF MS
Pyrosequencing
1
0.9
0.8
0.7
0.6
0.5
0.4
0.3
0.2
0.1
0
M4
M5.6
M7
M9
M10.11
M18.19
M20.21.22
M23.24
M26.27.28
M29
P1
P2
P3
P4
P5
P6
P7

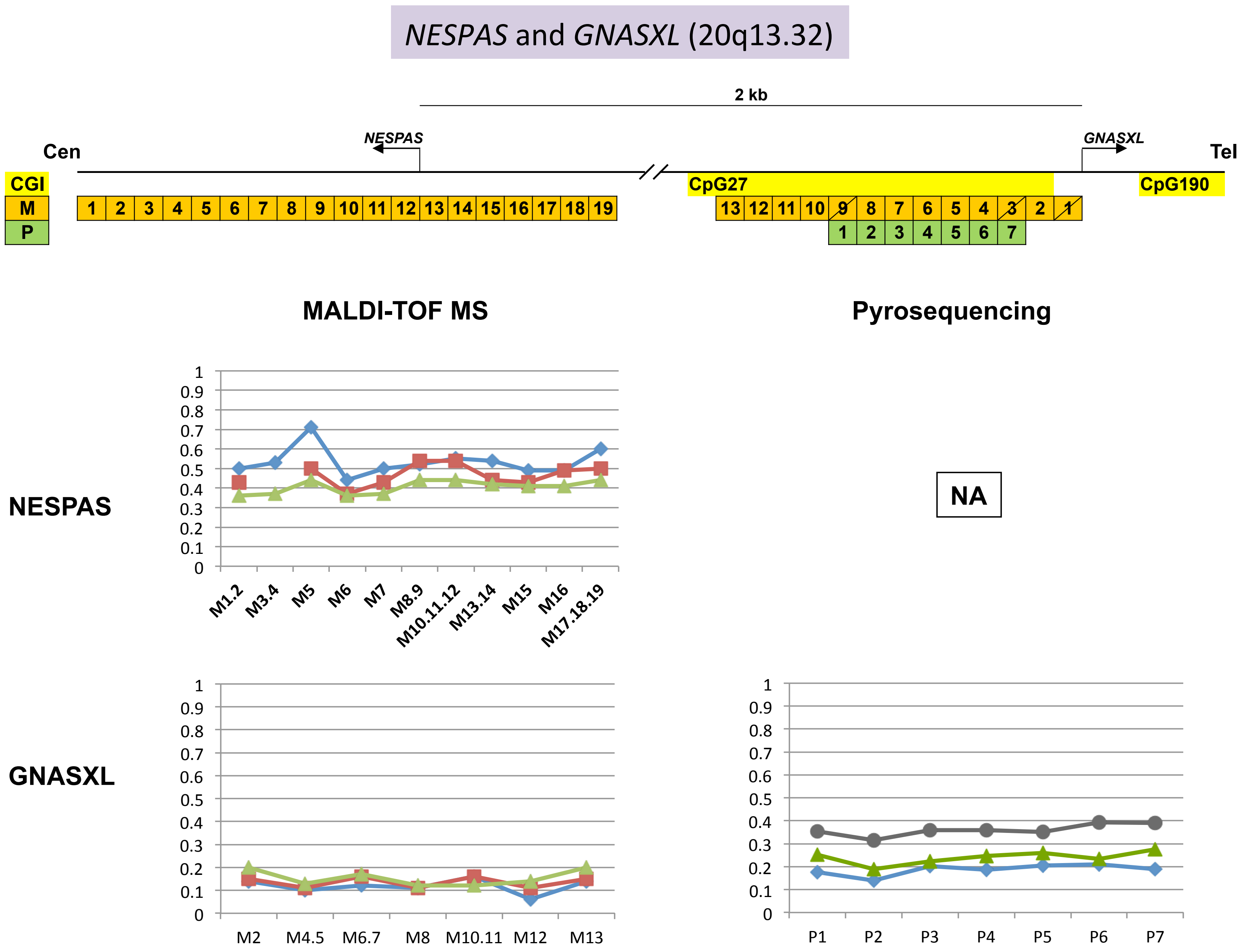
NESPAS and GNASXL (20q13.32)
2 kb
Cen
NESPAS
GNASXL
Tel
CGI
M
P
CpG27
CpG190
MALDI-TOF MS
Pyrosequencing
NESPAS
GNASXL
NA
M1.2
M3.4
M5
M6
M7
M8.9
M10.11.12
M13.14
M15
M16
M17.18.19
M2
M4.5
M6.7
M8
M10.11
M12
M13
P1
P2
P3
P4
P5
P6
P7

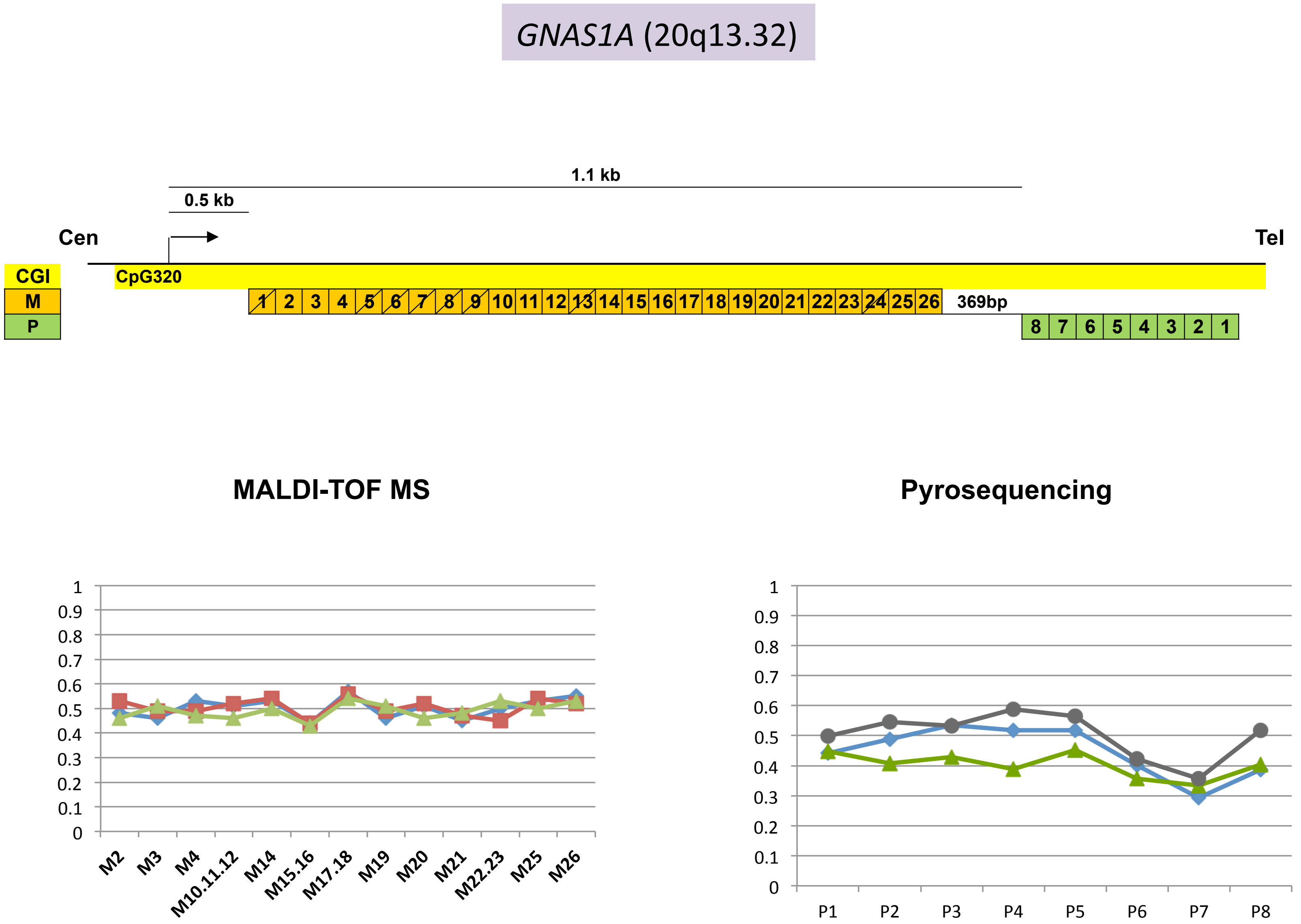

GNAS1A (20q13.32)
1.1 kb
0.5 kb
Cen
Tel
CGI
M
P
CpG320
1 2 3 4 5 6 7 8 9 10 11 12 13 14 15 16 17 18 19 20 21 22 23 24 25 26
369bp
8 7 6 5 4 3 2 1
MALDI-TOF MS
Pyrosequencing
1
0.9
0.8
0.7
0.6
0.5
0.4
0.3
0.2
0.1
0
M2 M3 M4 M10.11.12 M14 M15.16 M17.18 M19 M20 M21 M22.23 M25 M26
P1 P2 P3 P4 P5 P6 P7 P8
