# Supplementary material for: Comprehensive analyses of imprinted differentially methylated regions reveal epigenetic and genetic characteristics in hepatoblastoma
Source: BMC Cancer. 2013 Dec 27;13:608. doi: 10.1186/1471-2407-13-608 (PMC3880457; doi:10.1186/1471-2407-13-608)

Figure S2

ARHI-CG1 HpB07

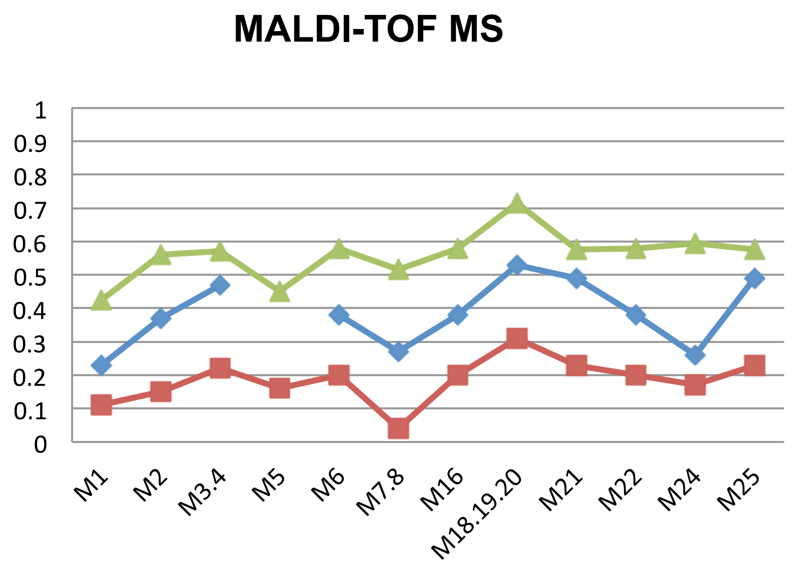

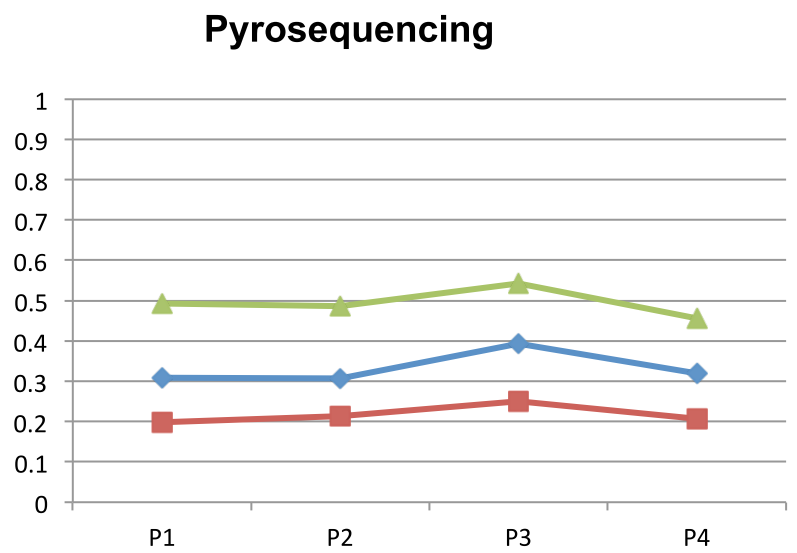

ZDBF2 HpB05

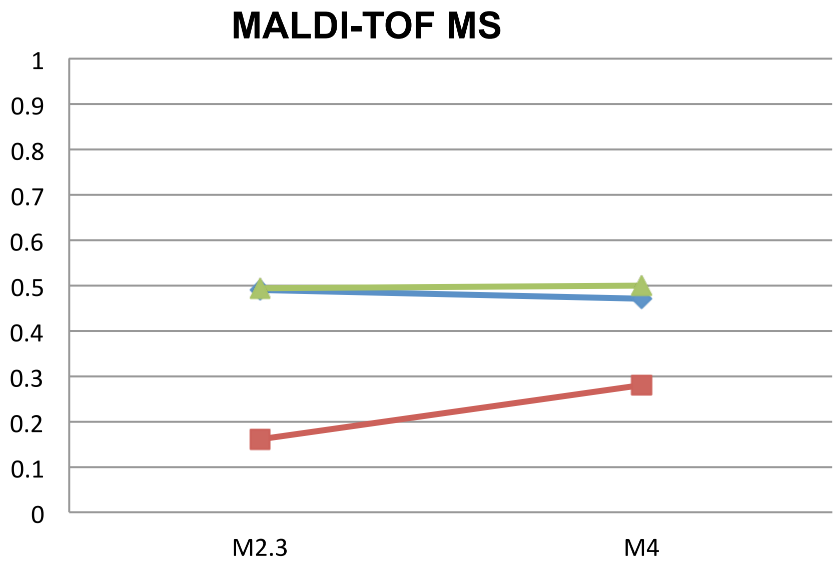

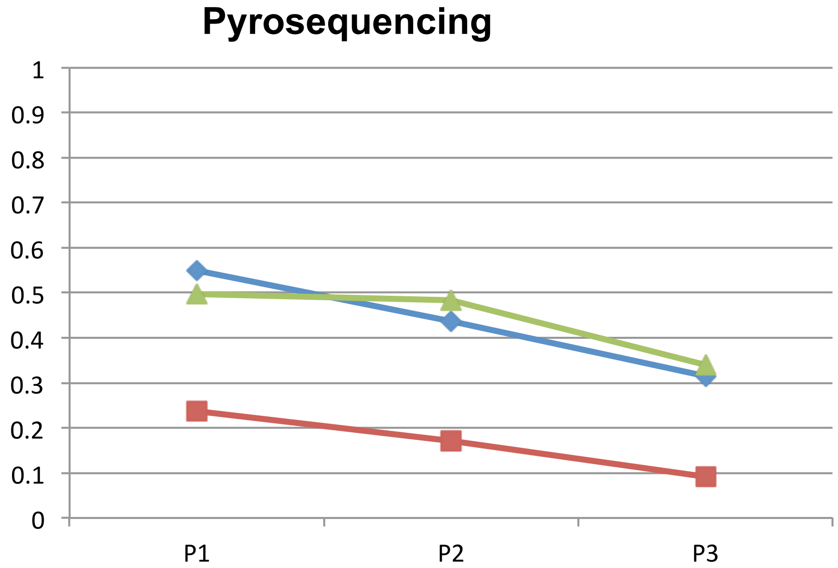

ZDBF2 HpB09

MALDI-TOF MS

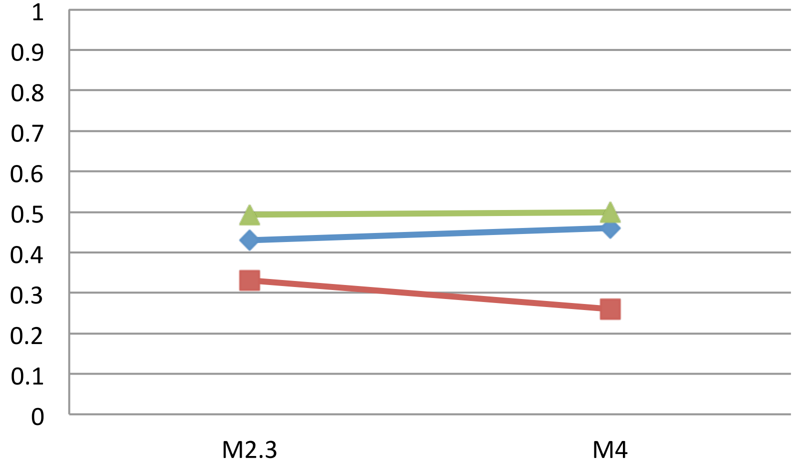

Pyrosequencing

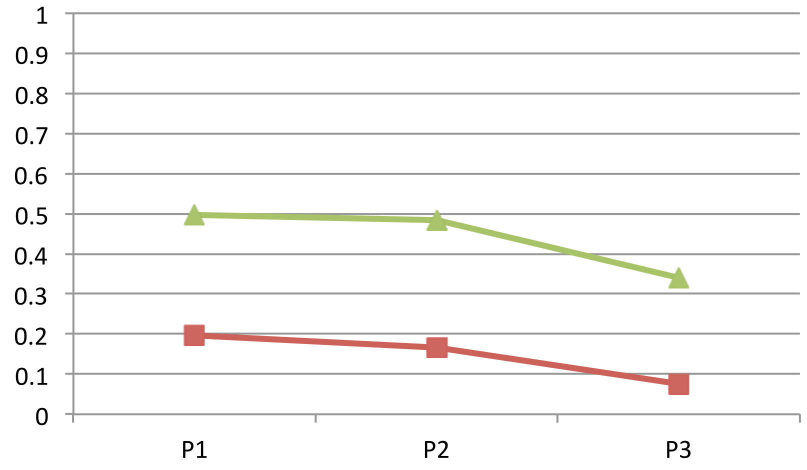

NAP1L5 HpB05

MALDI-TOF MS

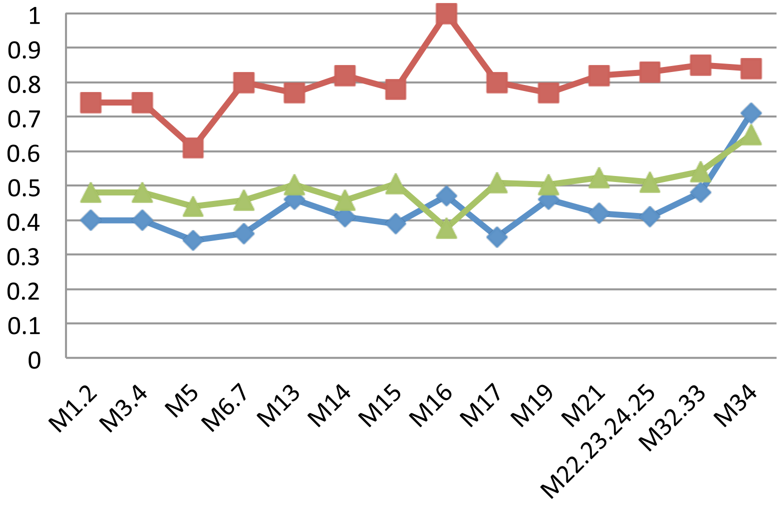

Pyrosequencing

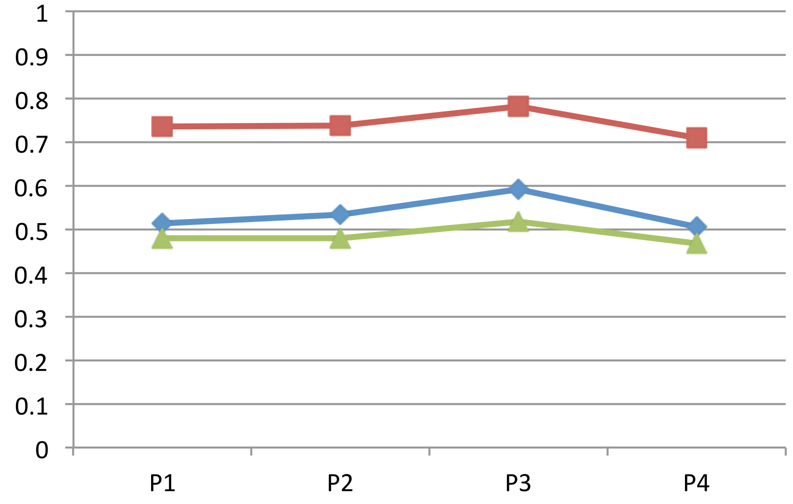

PEG10 HpB11

MALDI-TOF MS

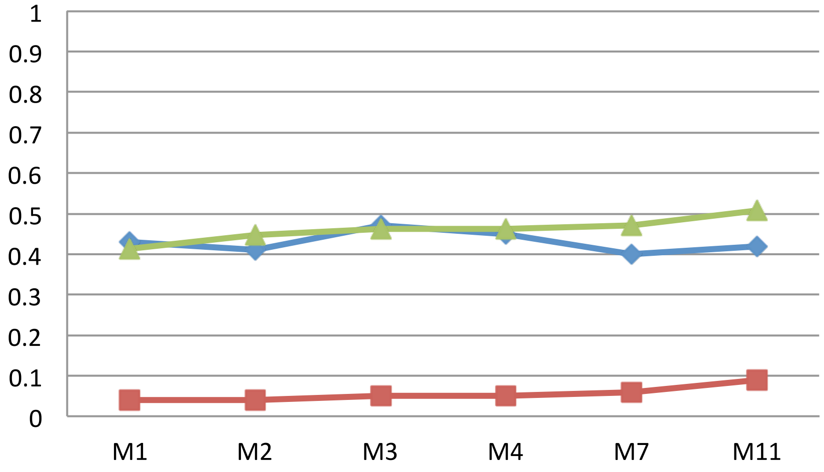

Pyrosequencing

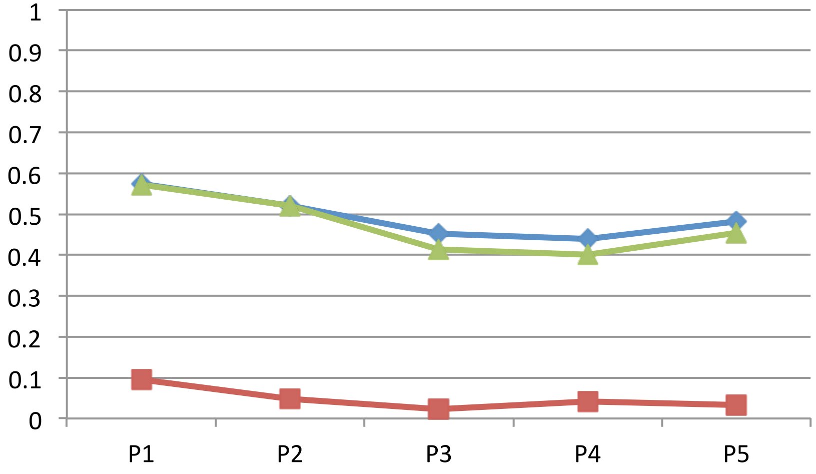

INPP5FV2 HpB01

MALDI-TOF MS

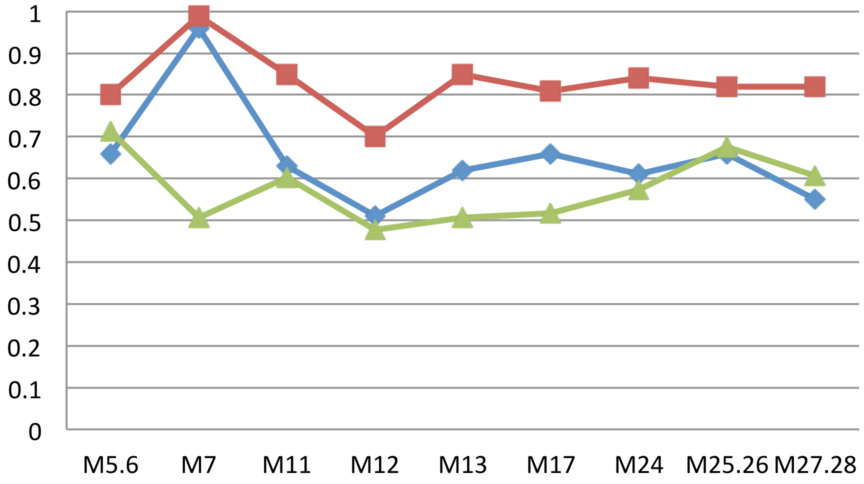

Pyrosequencing

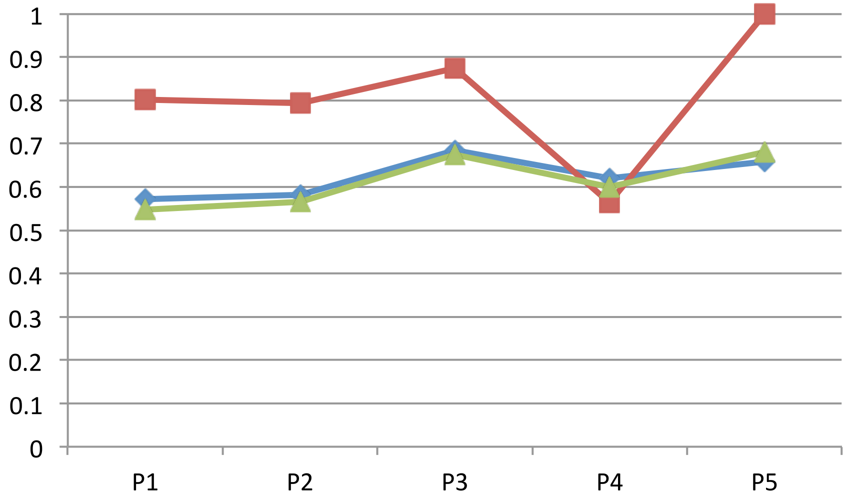

INPP5FV2 HpB02

MALDI-TOF MS

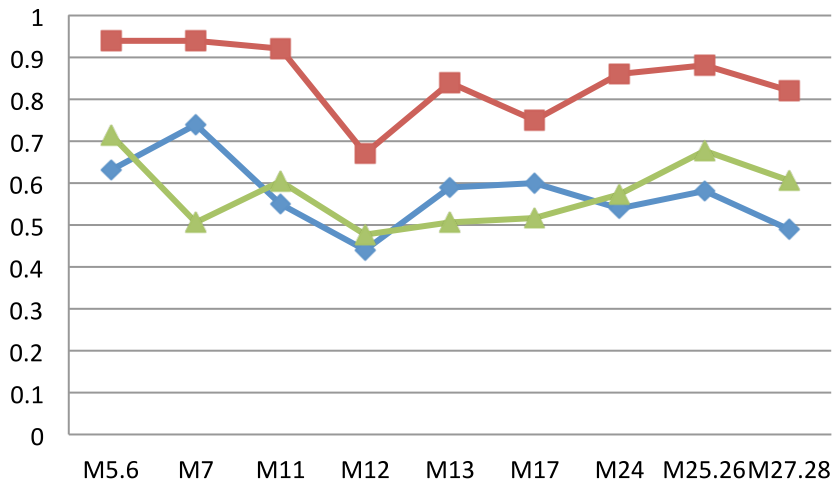

Pyrosequencing

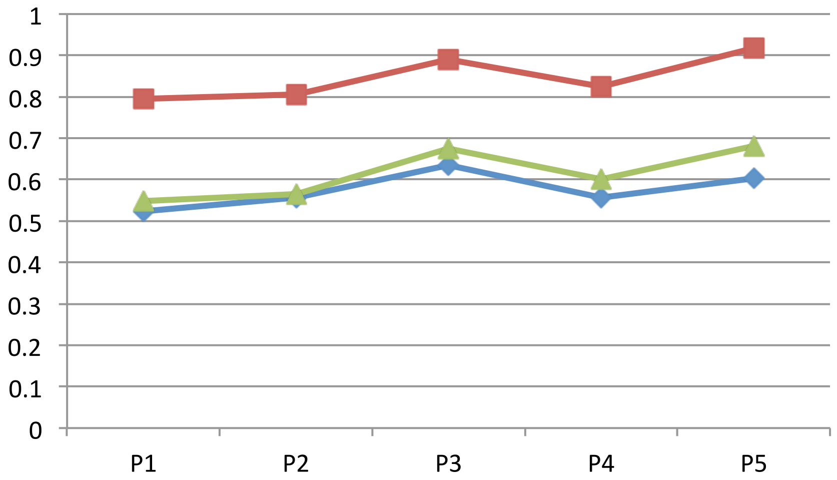

INPP5FV2 HpB04

MALDI-TOF MS

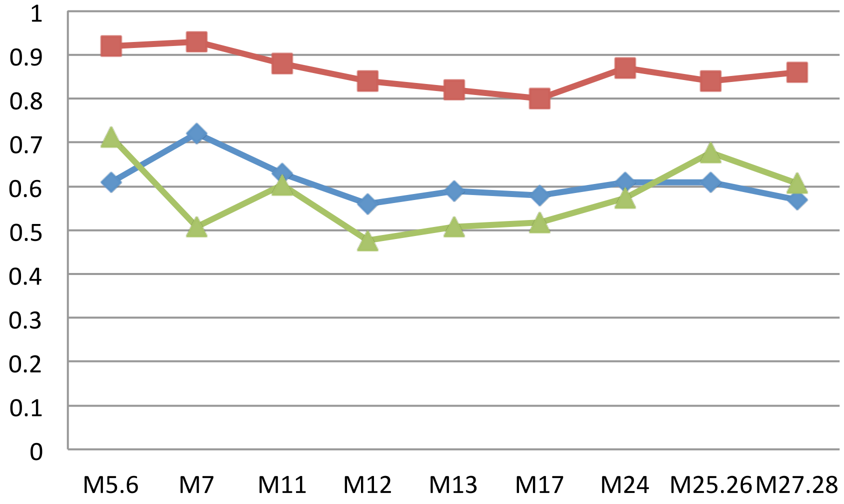

Pyrosequencing

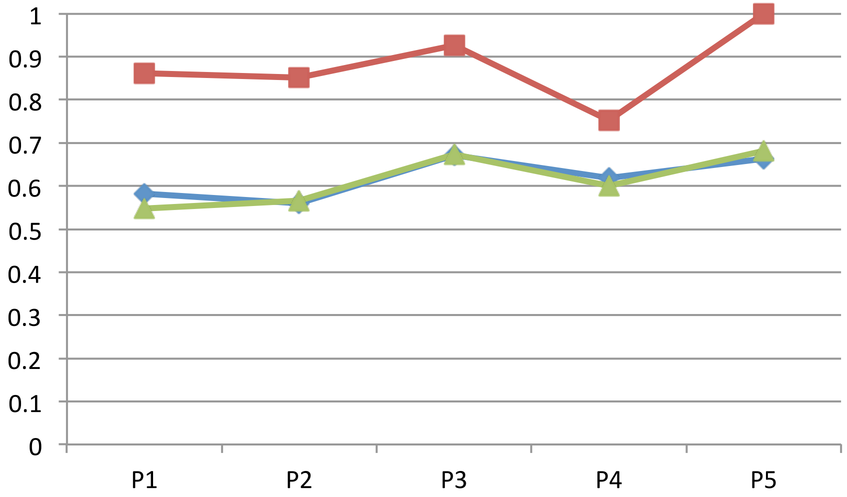

INPP5FV2 HpB05

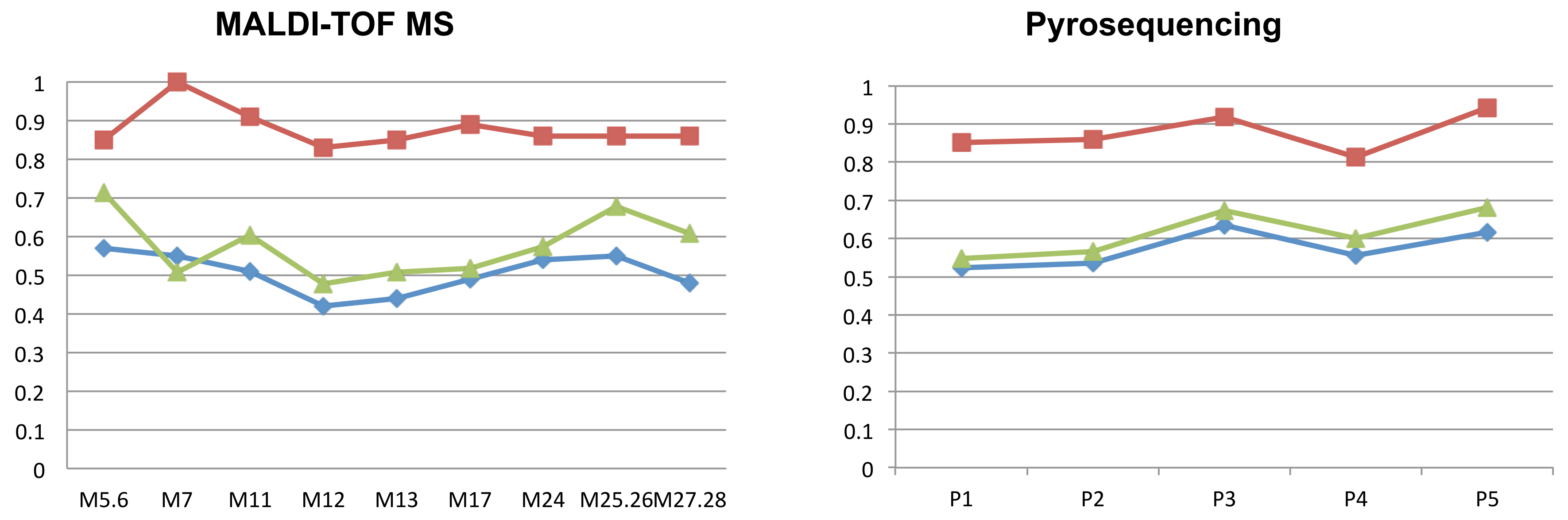

INPP5FV2 HpB07

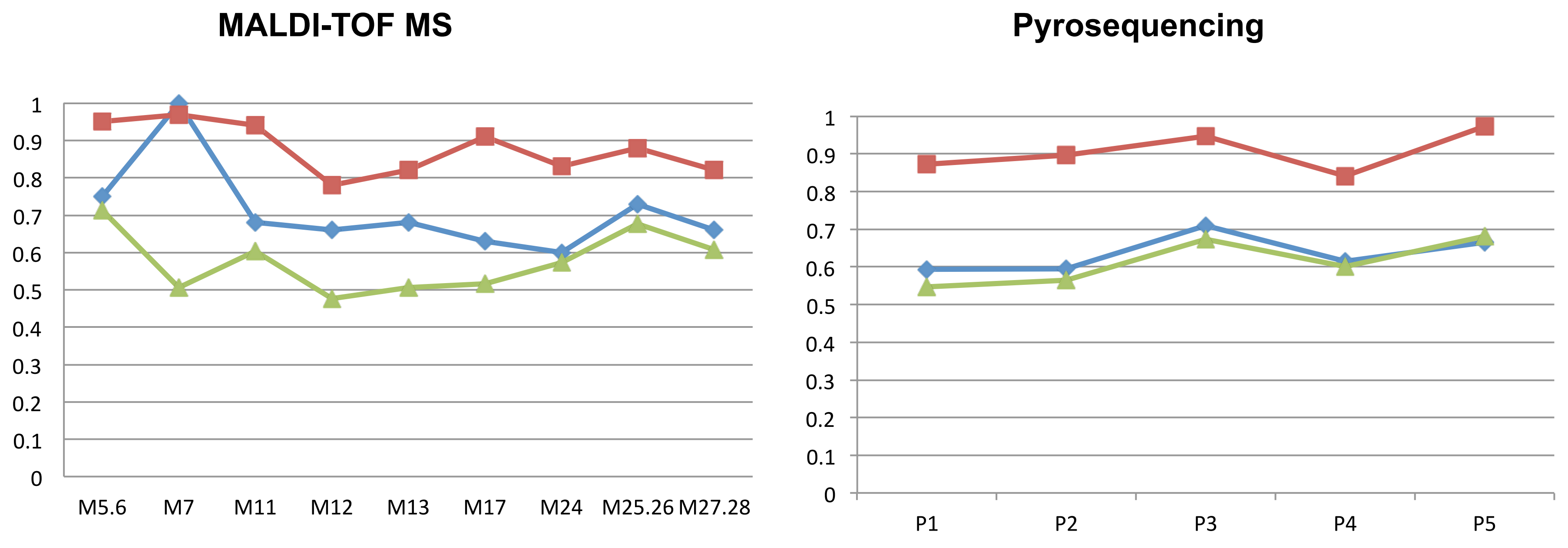

INNP5FV2 HpB09

MALDI-TOF MS

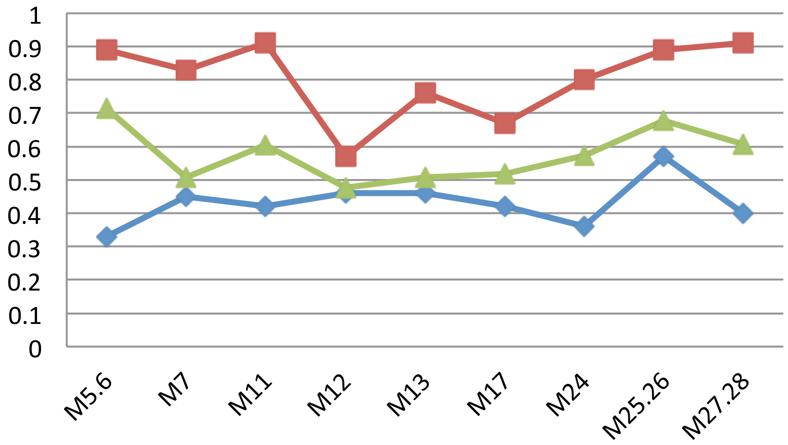

Pyrosequencing

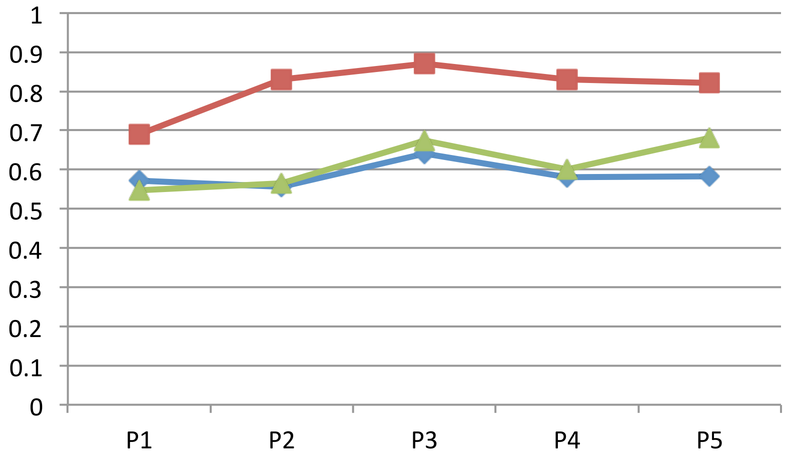

INPP5FV2 HpB11

MALDI-TOF MS

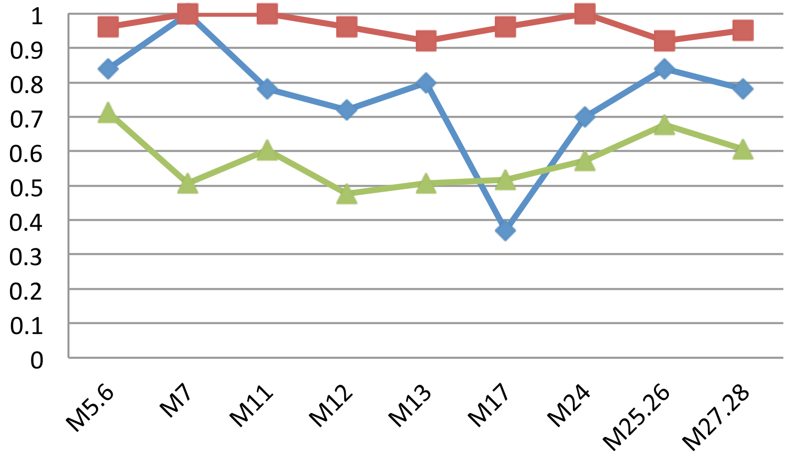

Pyrosequencing

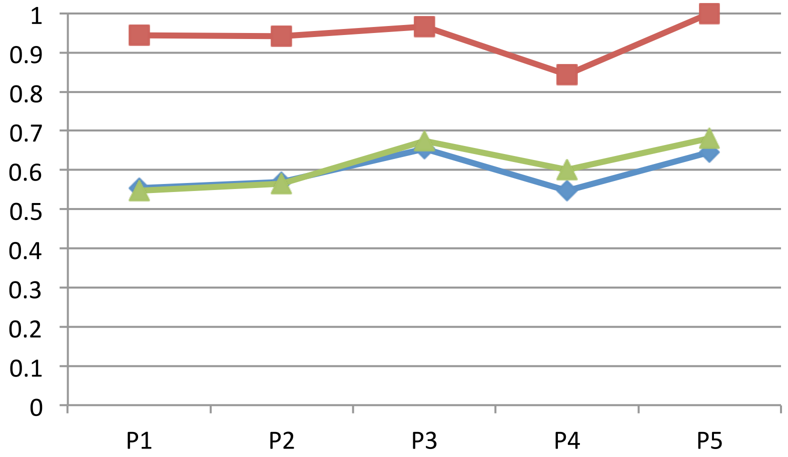

H19 promoter HpB05

MALDI-TOF MS

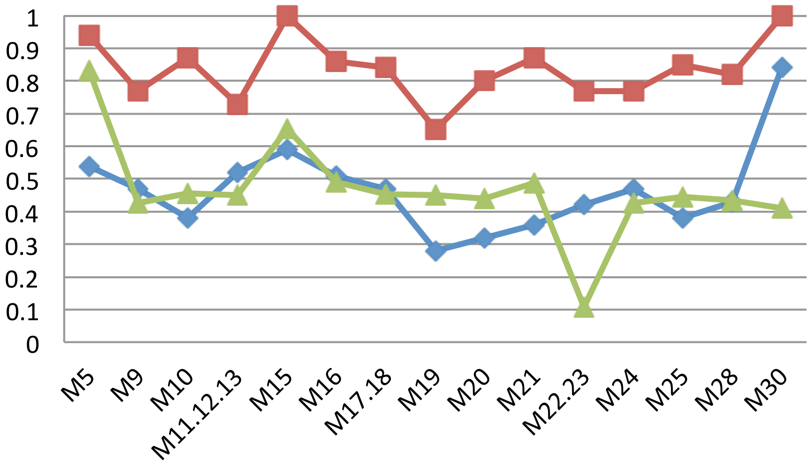

Pyrosequencing

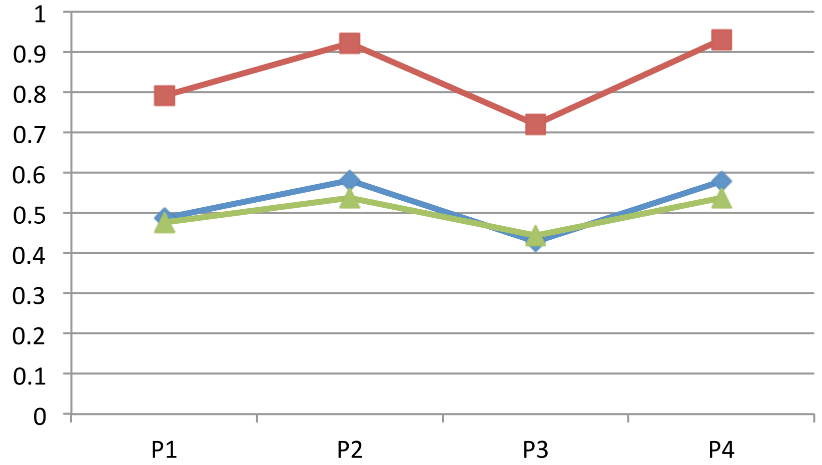

H19-DMR HpB04

MALDI-TOF MS

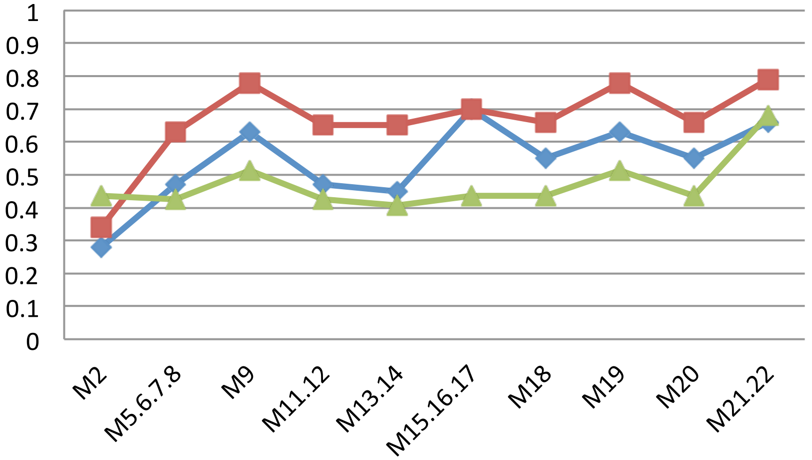

Pyrosequencing

NA
(see Supplementary Fig.3)

H19-DMR HpB05

MALDI-TOF MS

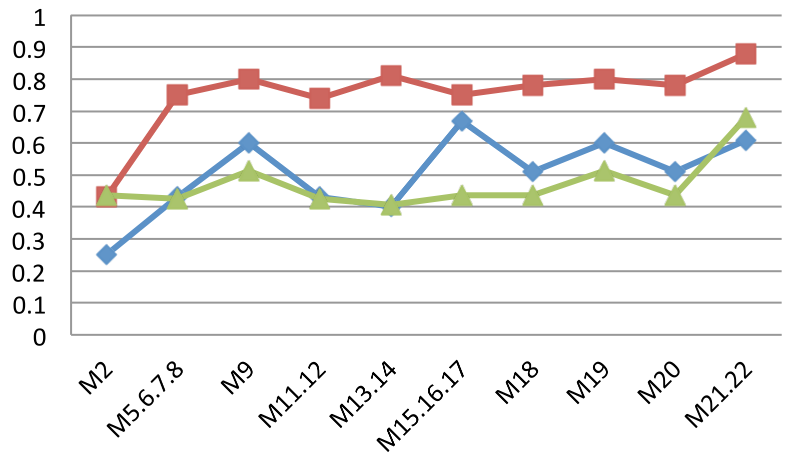

Pyrosequencing

NA
(see Supplementary Fig.3)

IGF2DMR0 HpB06

MALDI-TOF MS

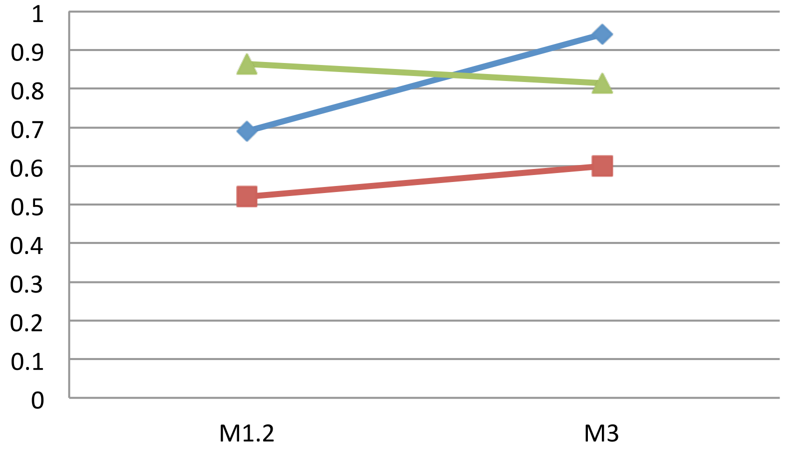

Pyrosequencing

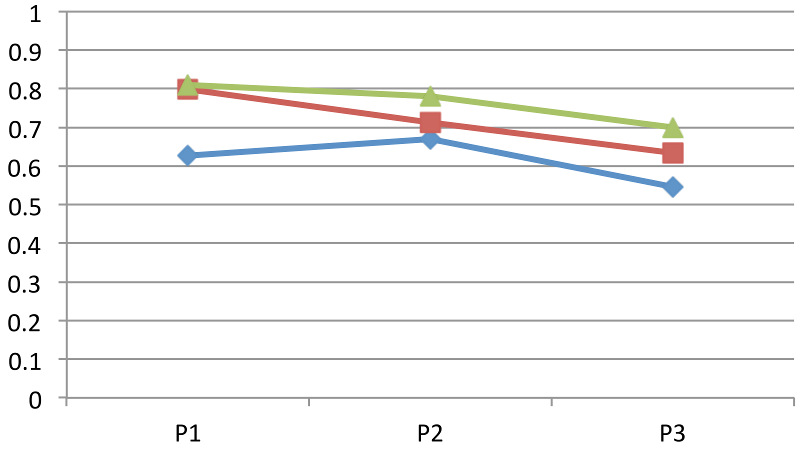

IGF2DMR0 HpB09

MALDI-TOF MS

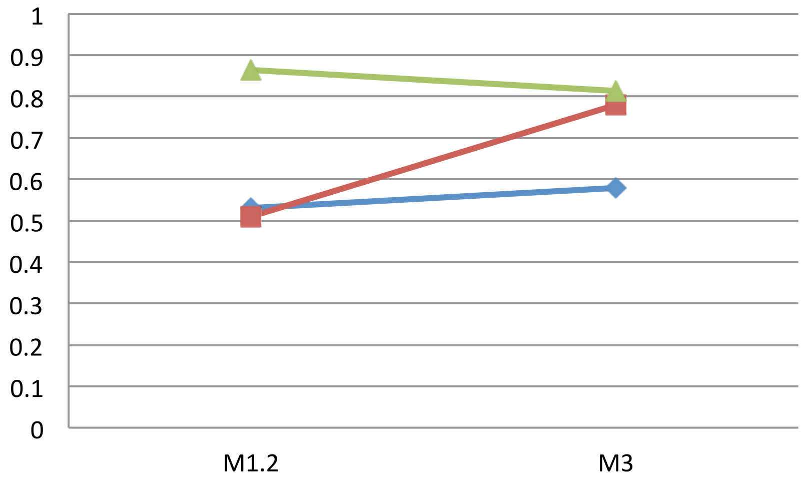

Pyrosequencing

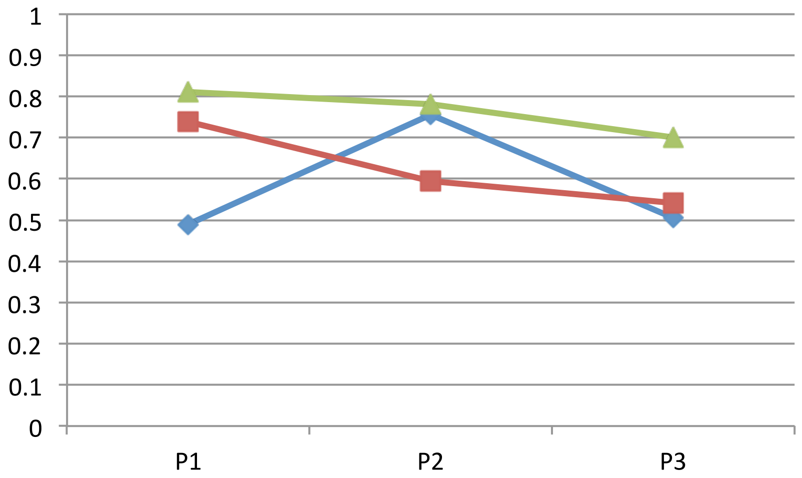

IGF2DMR0 HpB10

MALDI-TOF MS

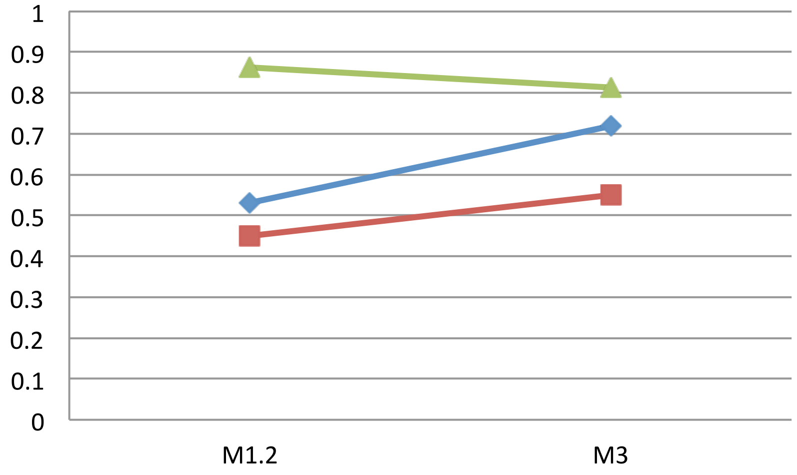

Pyrosequencing

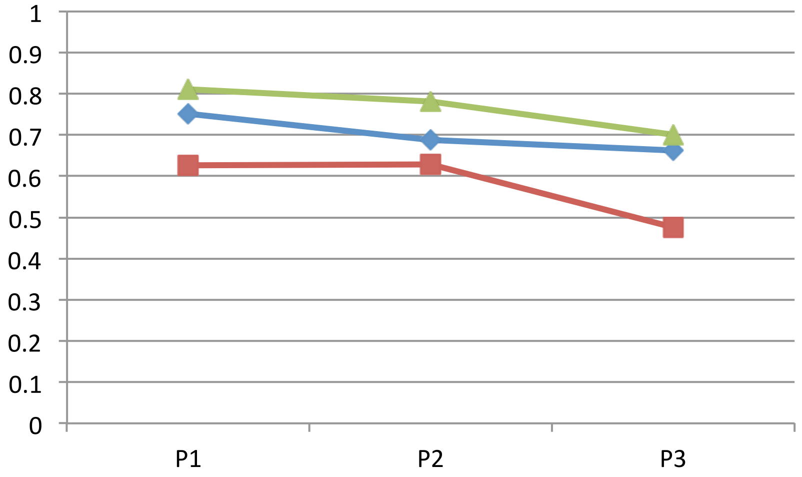

IGF2DMR2 HpB05

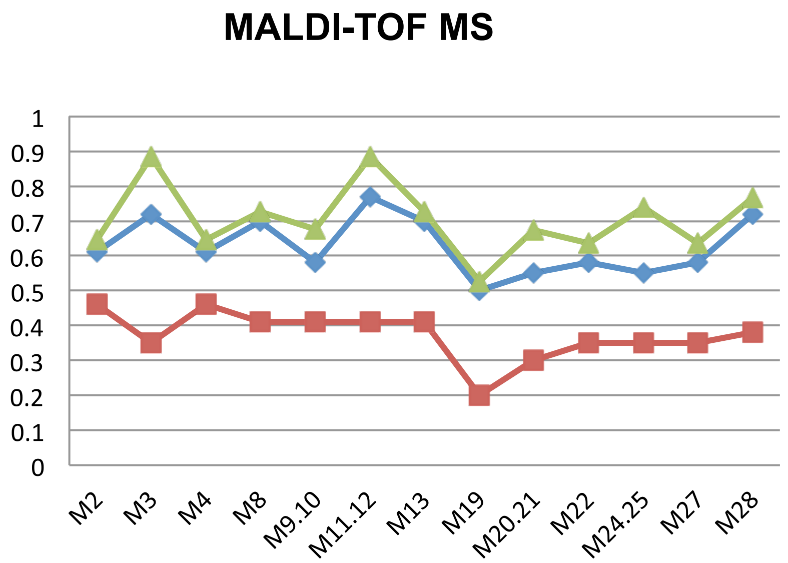

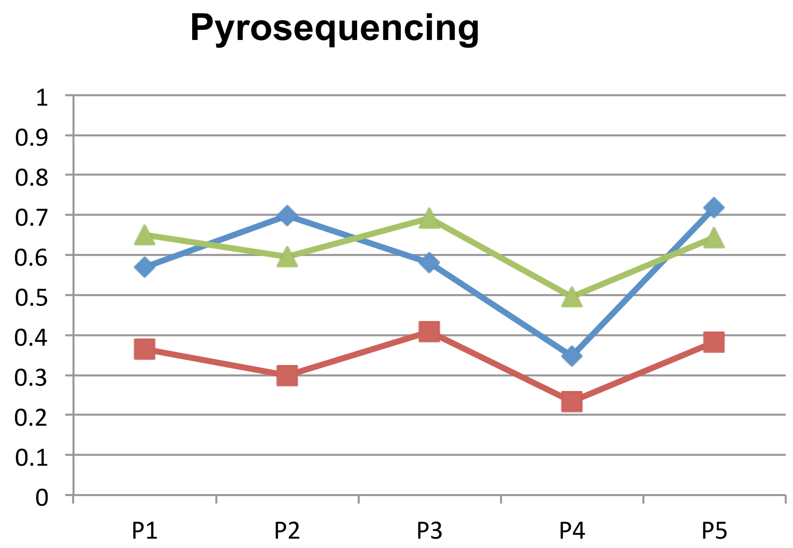

IGF2DMR2 HpB09

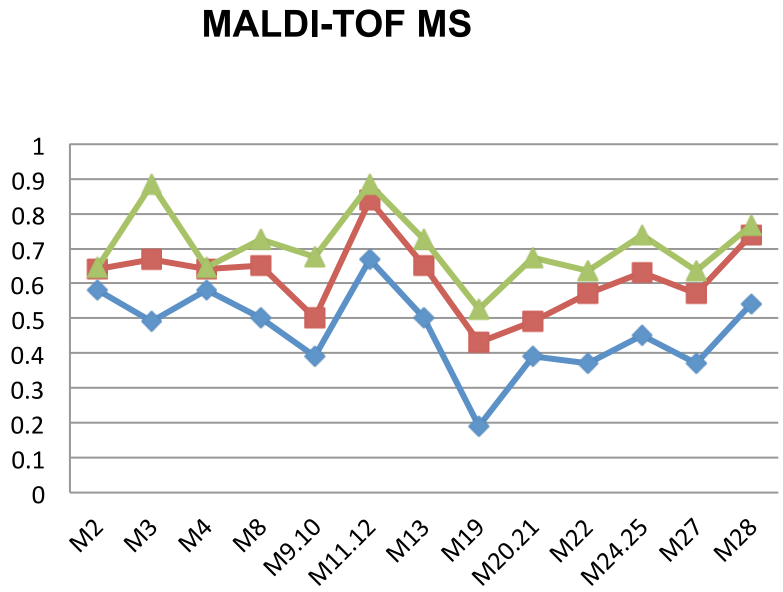

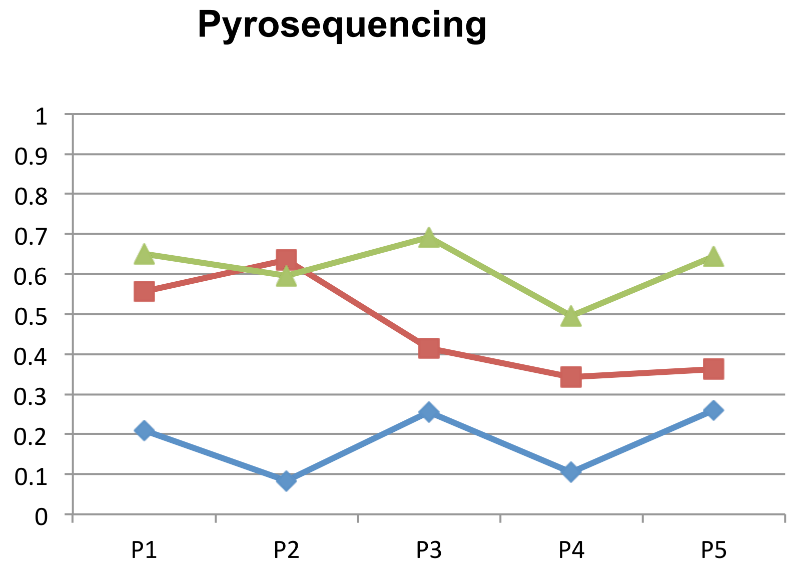

KvDMR1 HpB05

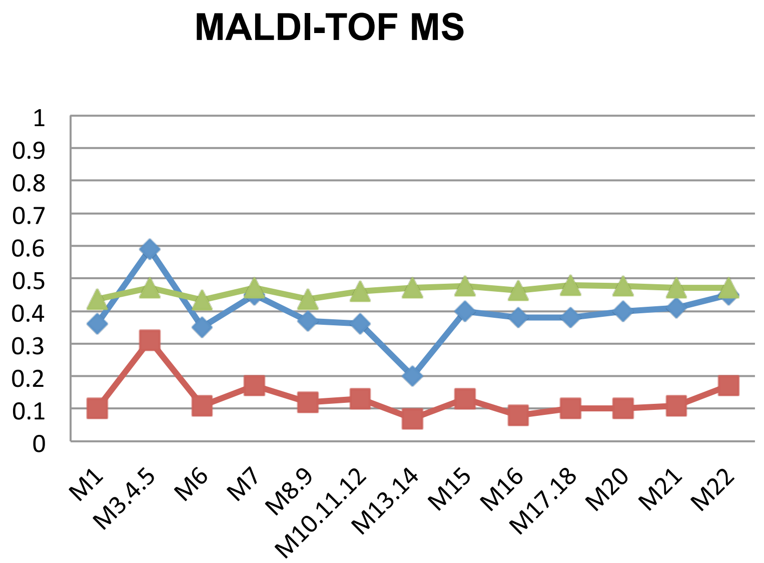

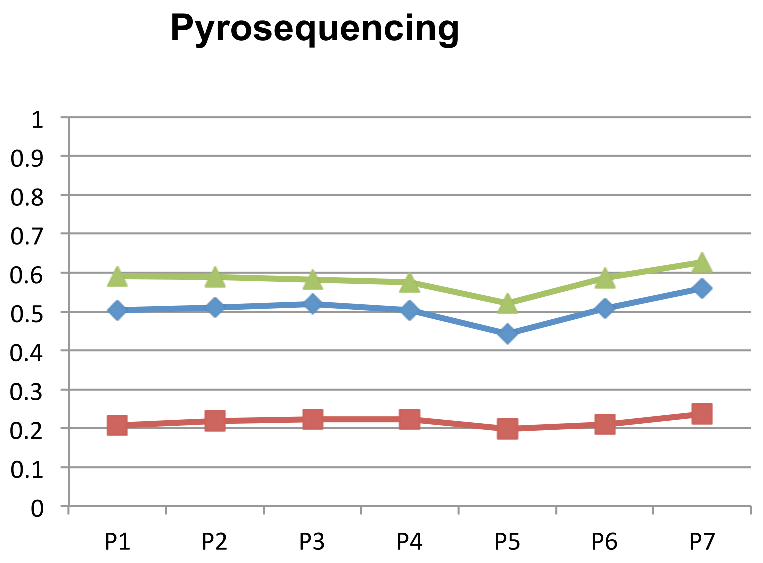

KvDMR1 HpB07

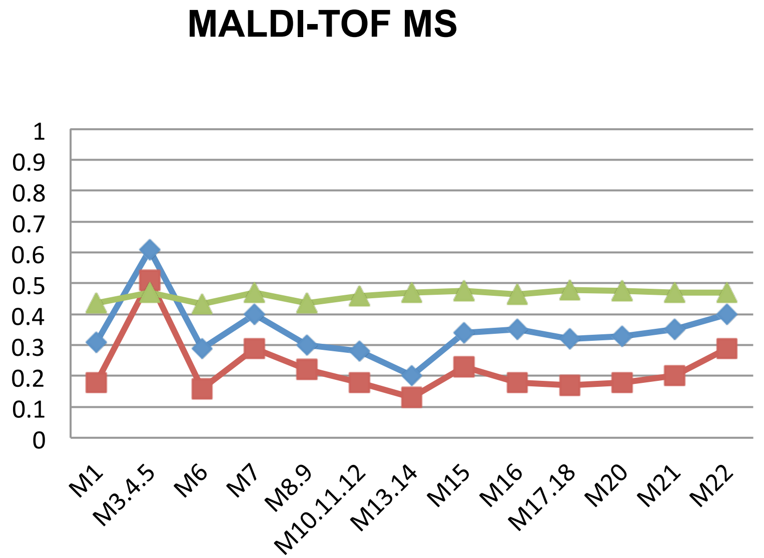

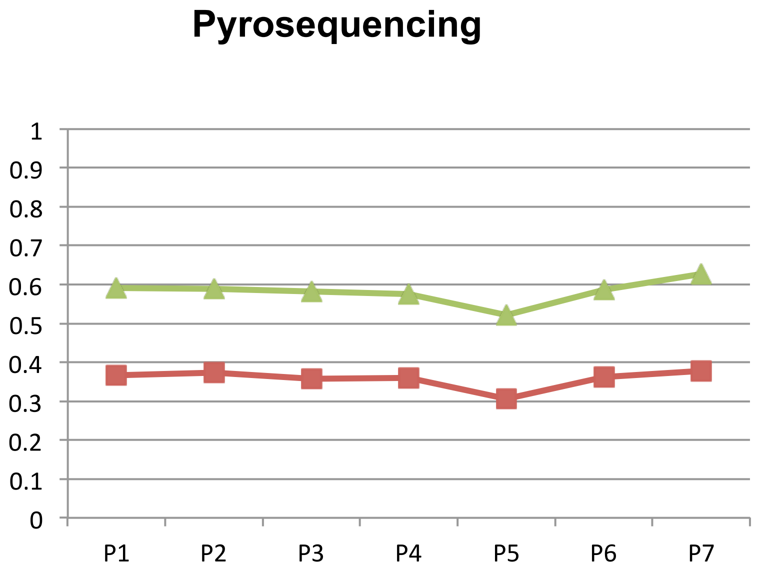

KvDMR1 HpB08

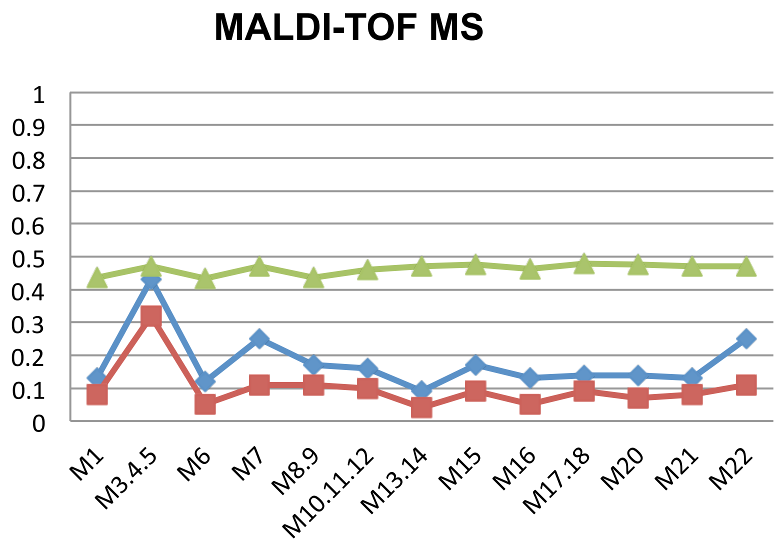

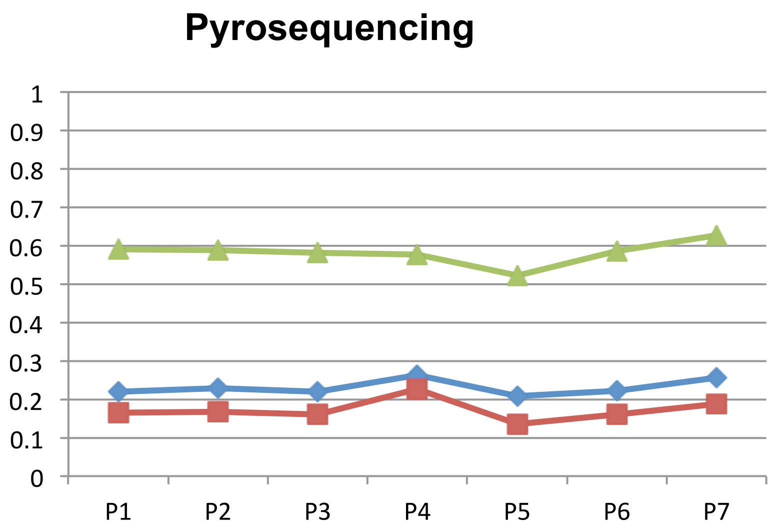

WT1AS HpB02

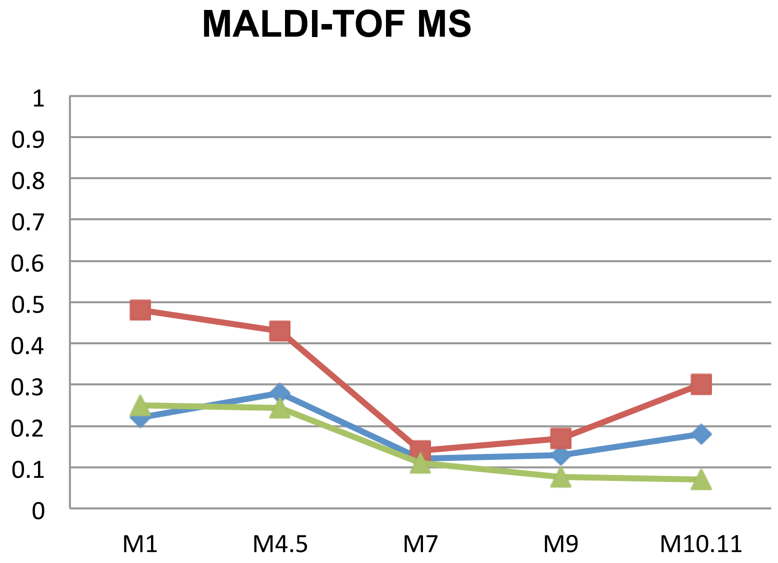

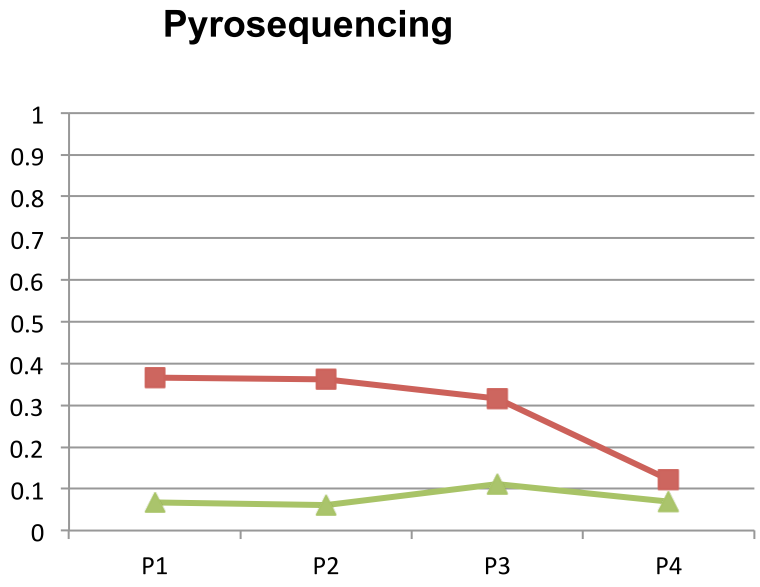

RB1 HpB01

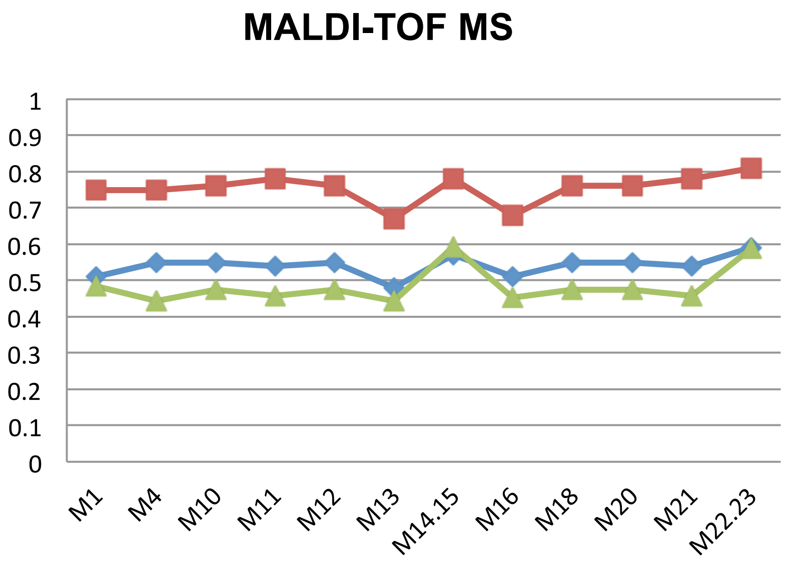

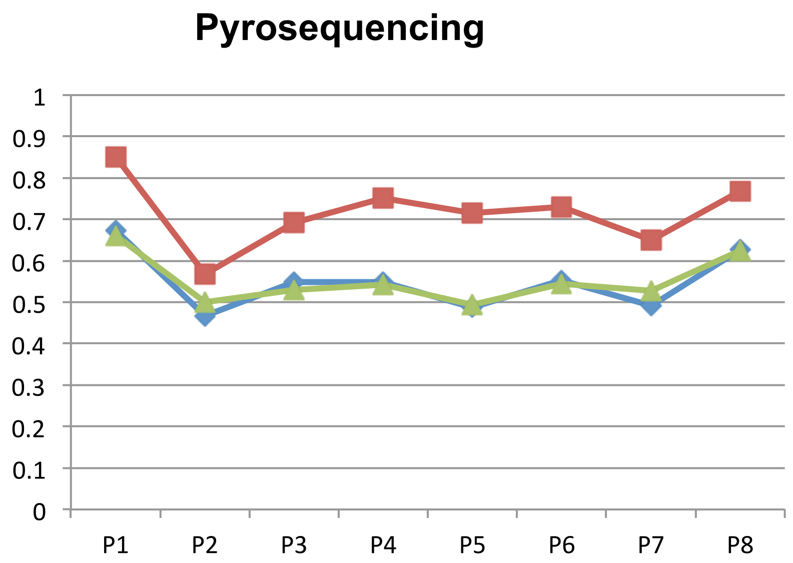

RB1 HpB05

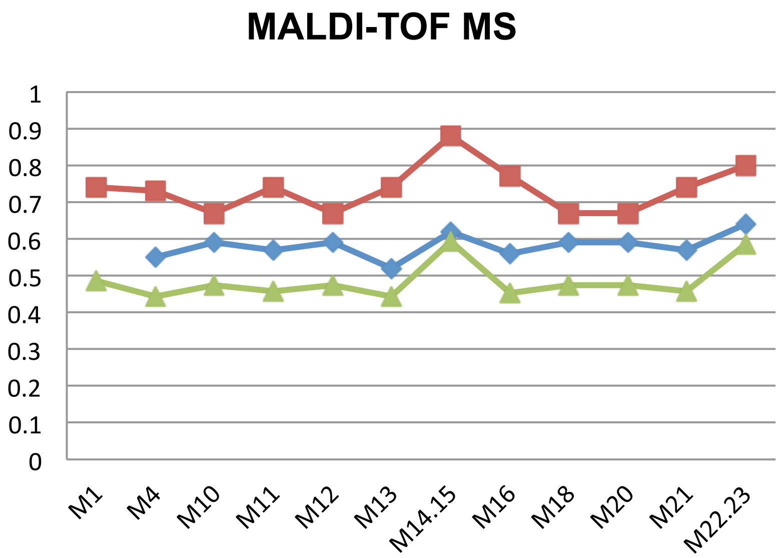

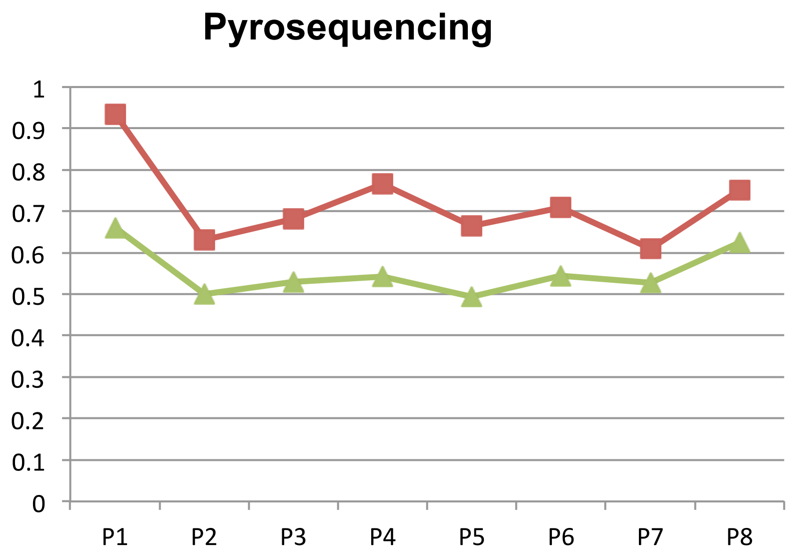

RB1 HpB10

MALDI-TOF MS

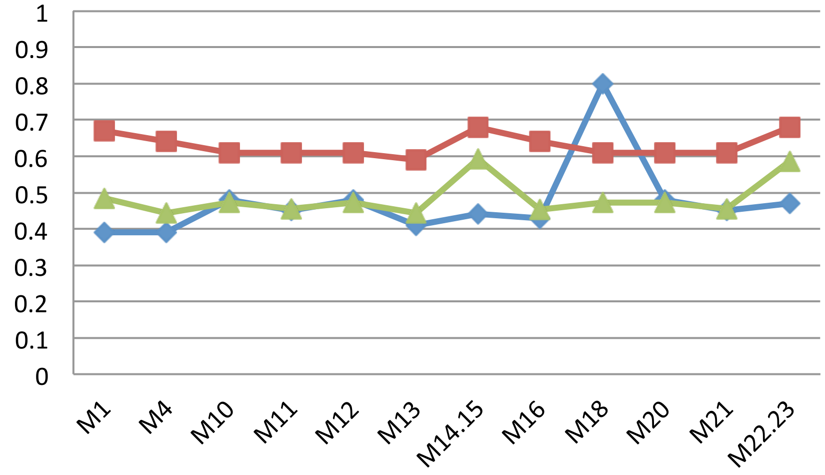

Pyrosequencing

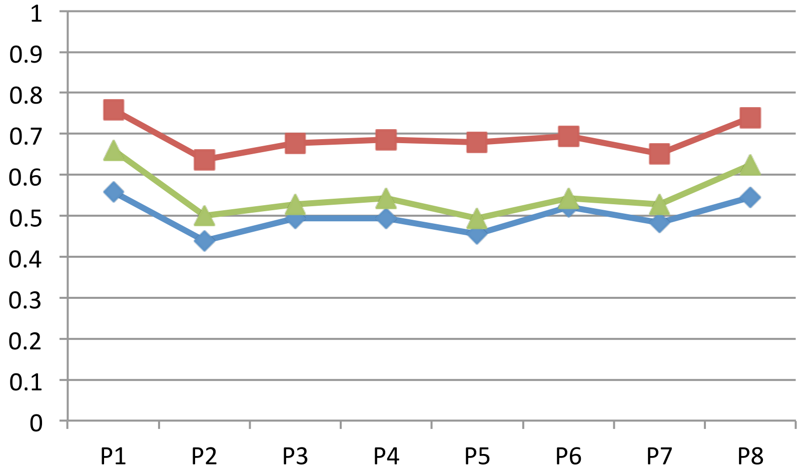

RB1 HpB11

MALDI-TOF MS

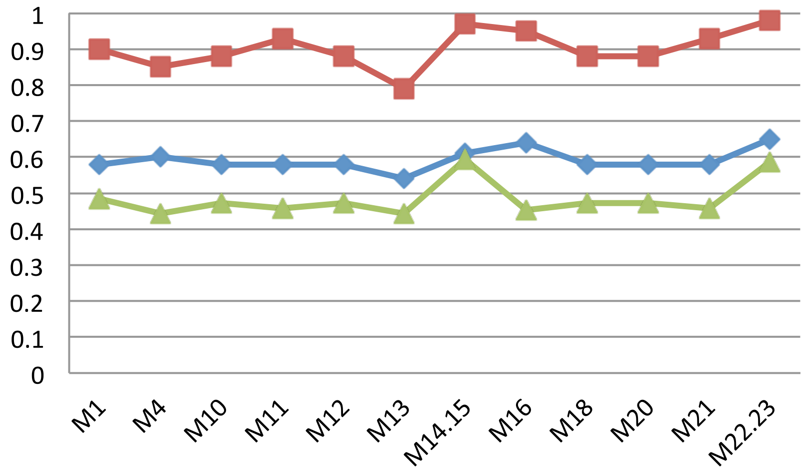

Pyrosequencing

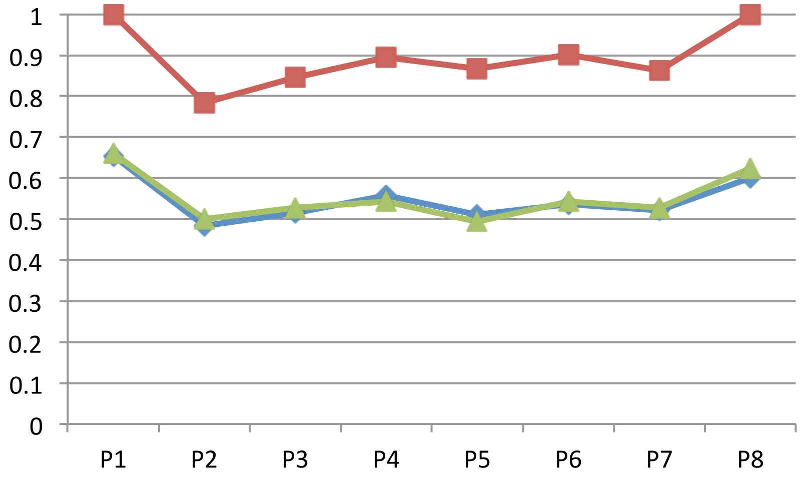

RB1 BWS109

MALDI-TOF MS

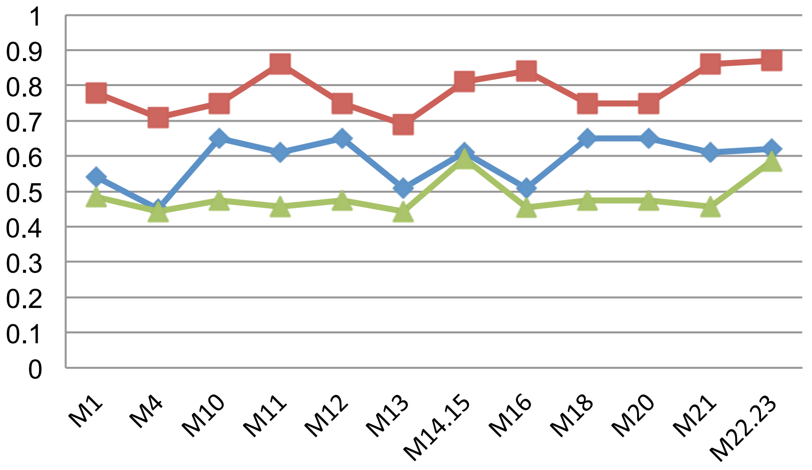

Pyrosequencing

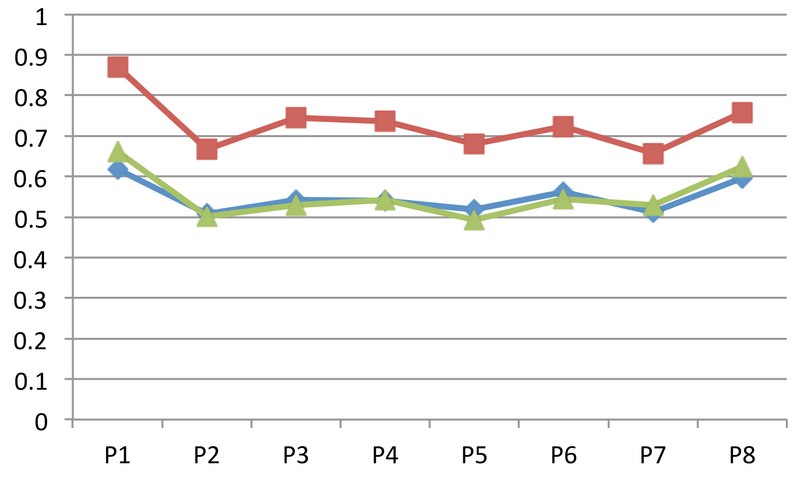

IG-DMR-CG4 HpB06

MALDI-TOF MS

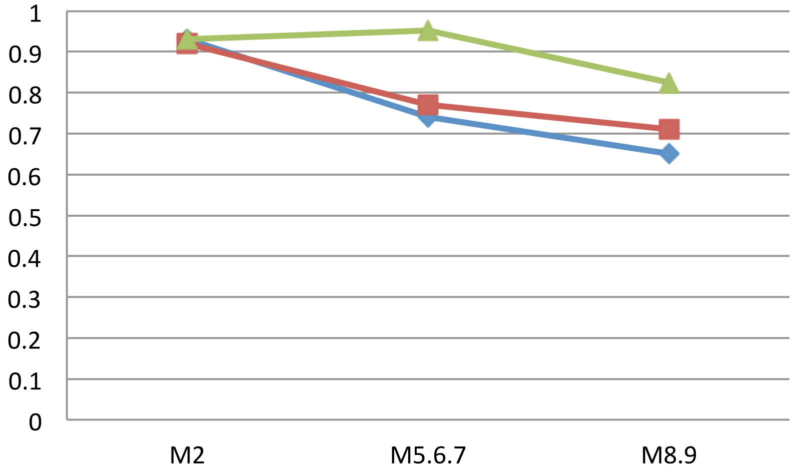

Pyrosequencing

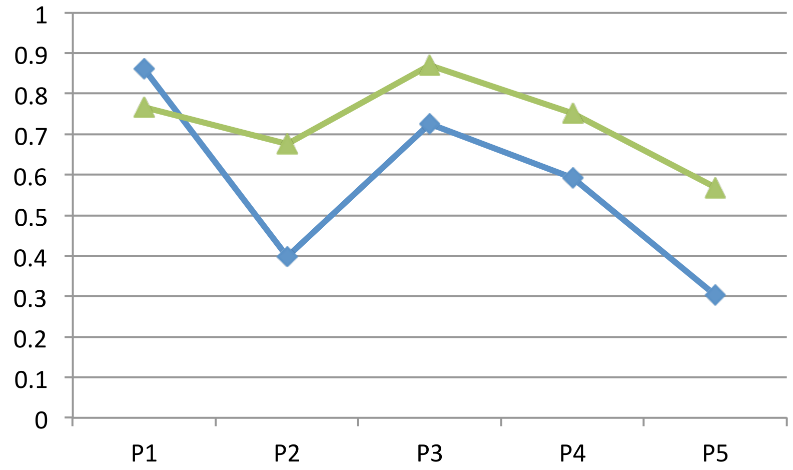

IG-DMR-CG4 HpB11

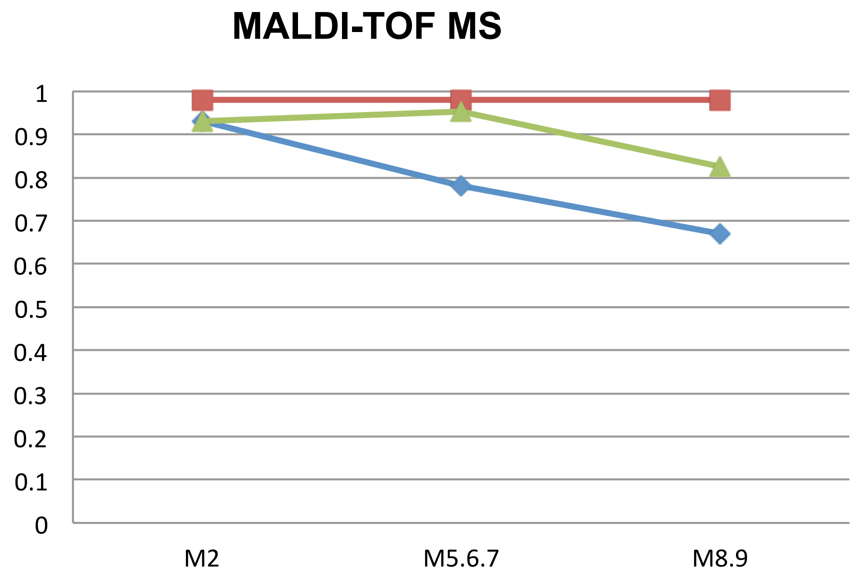

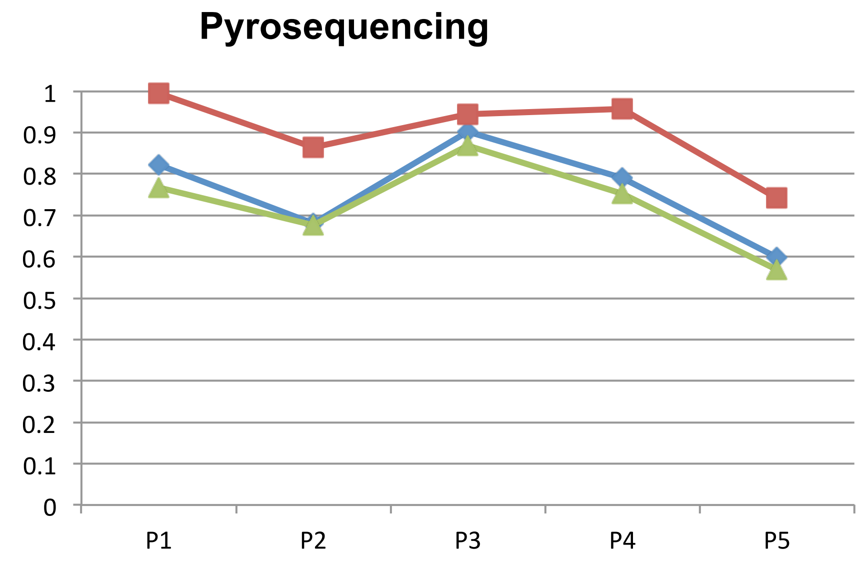

MEG3CG7 HpB11

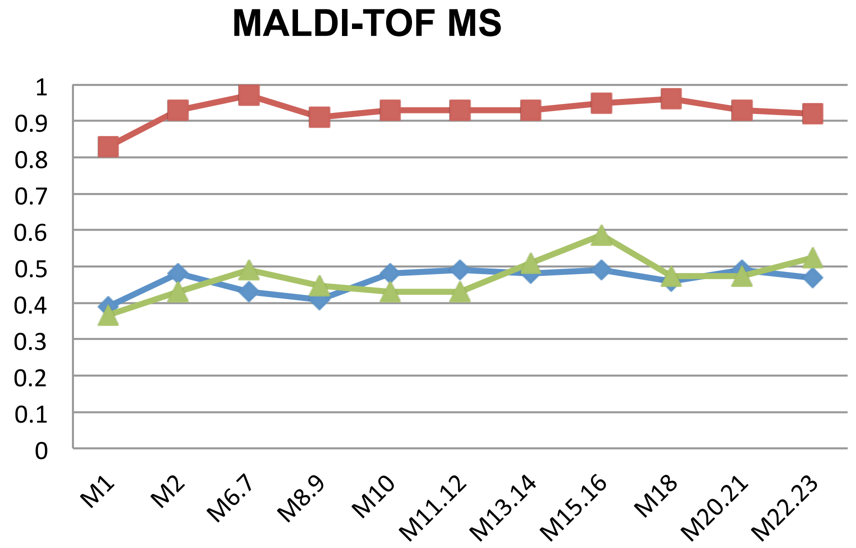

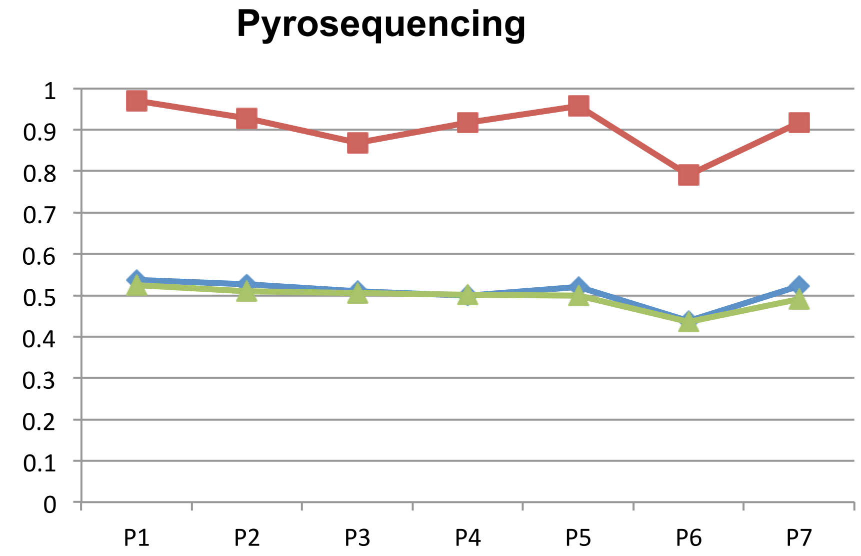

MCTS2 HpB03

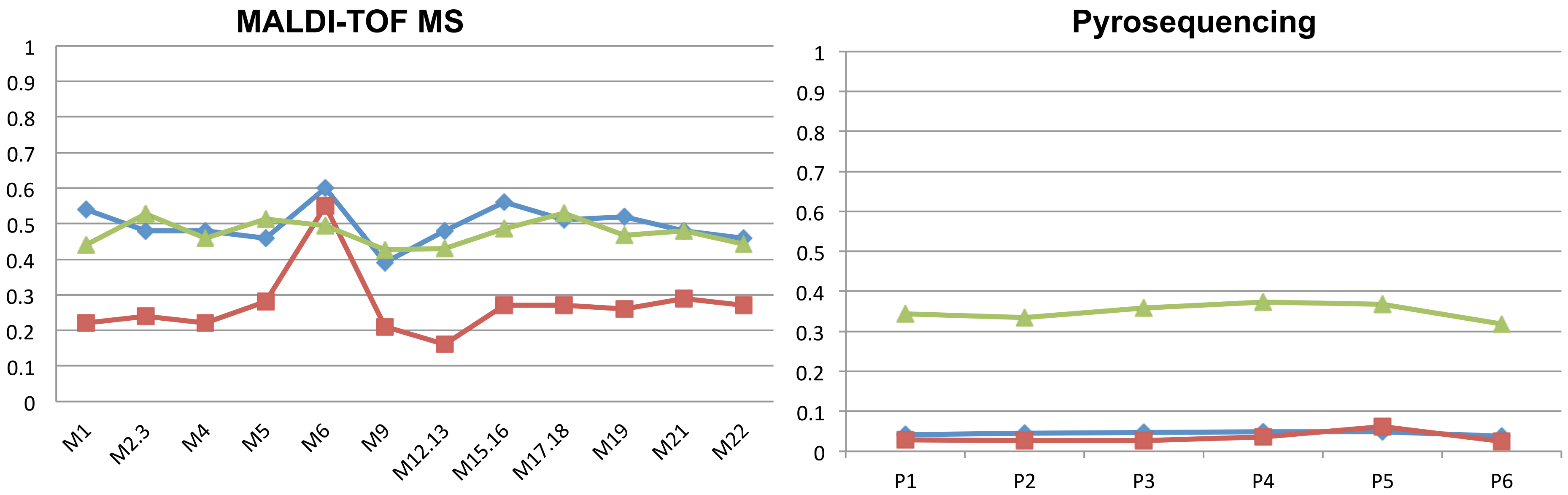

NESP55 HpB09

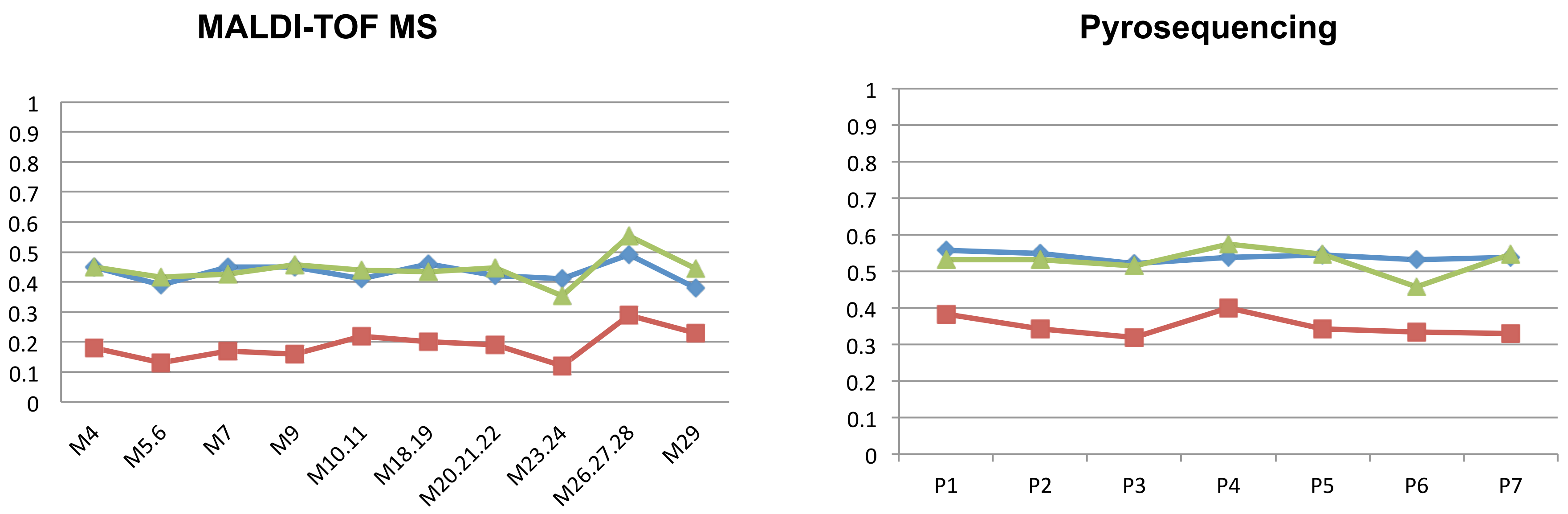

GNASXL HpB01

MALDI-TOF MS

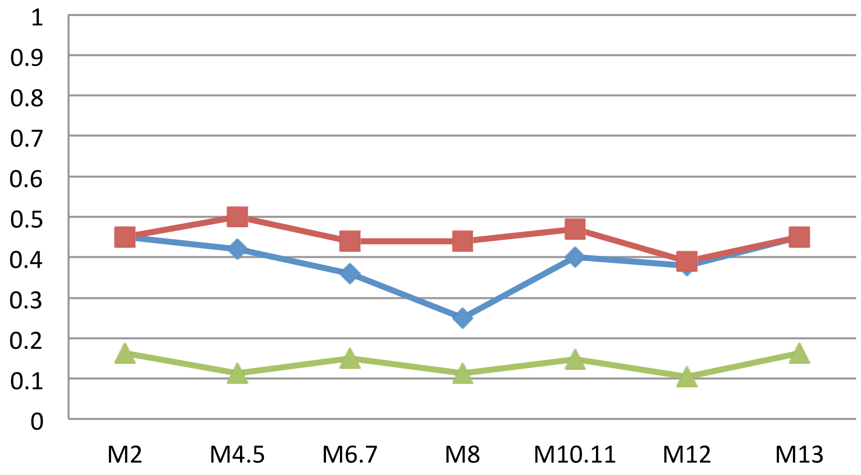

Pyrosequencing

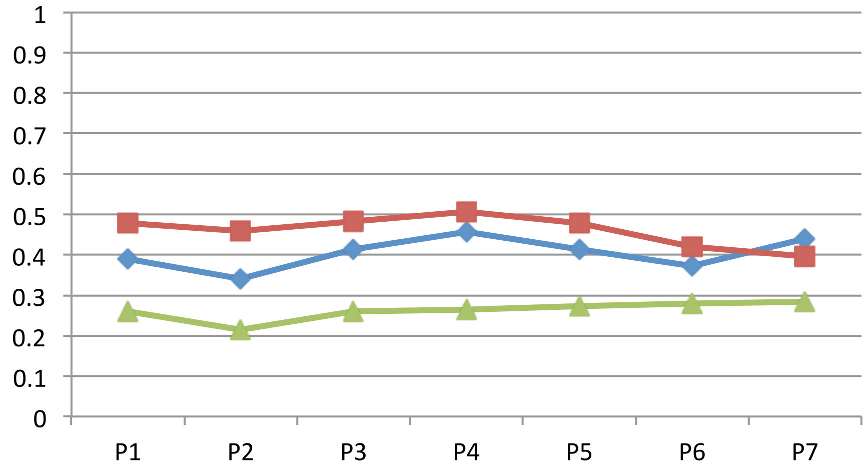

GNASXL HpB04

MALDI-TOF MS

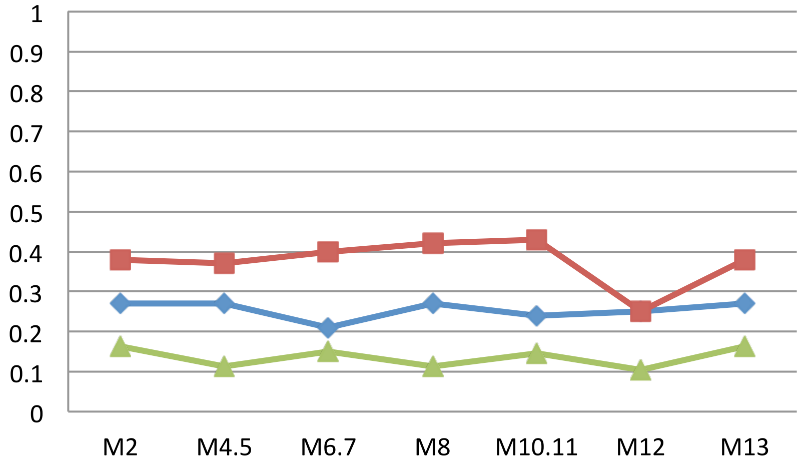

Pyrosequencing

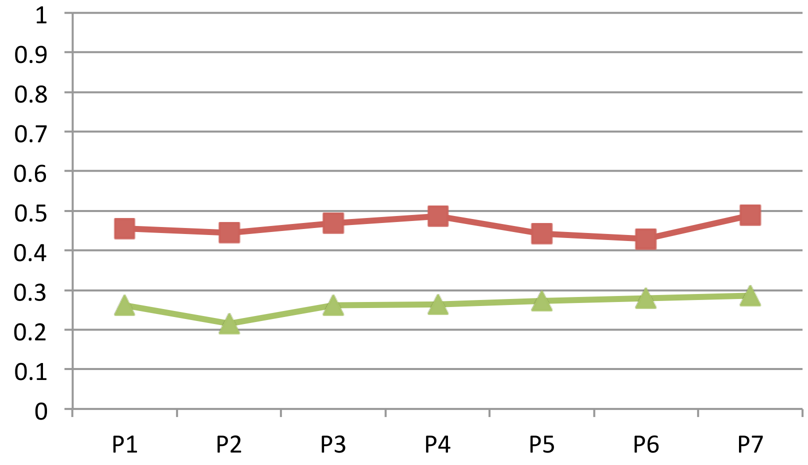

GNASXL HpB06

MALDI-TOF MS

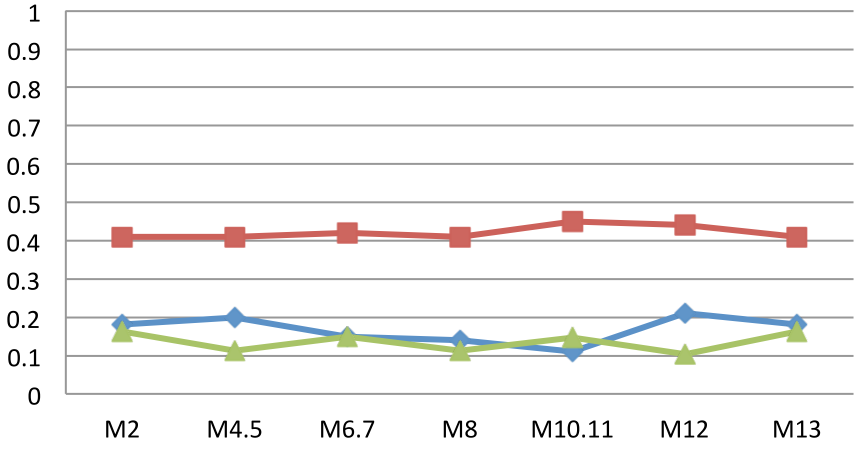

Pyrosequencing

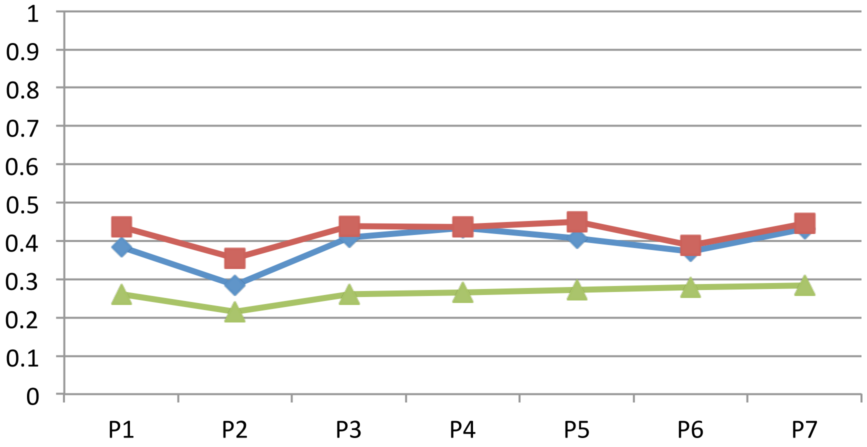

GNASXL BWS109

MALDI-TOF MS

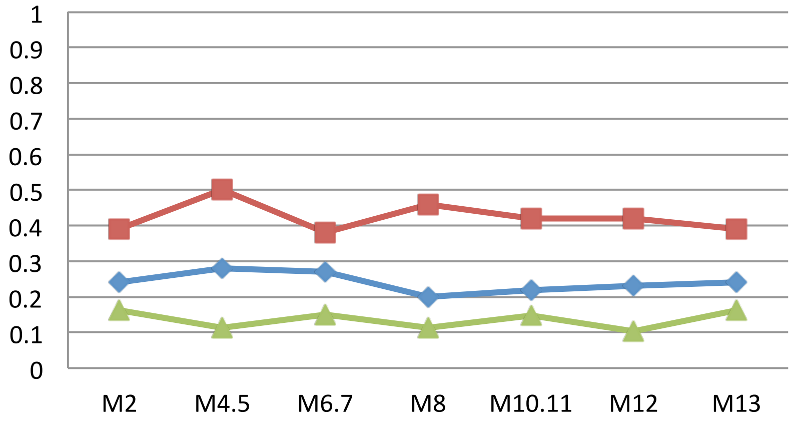

Pyrosequencing

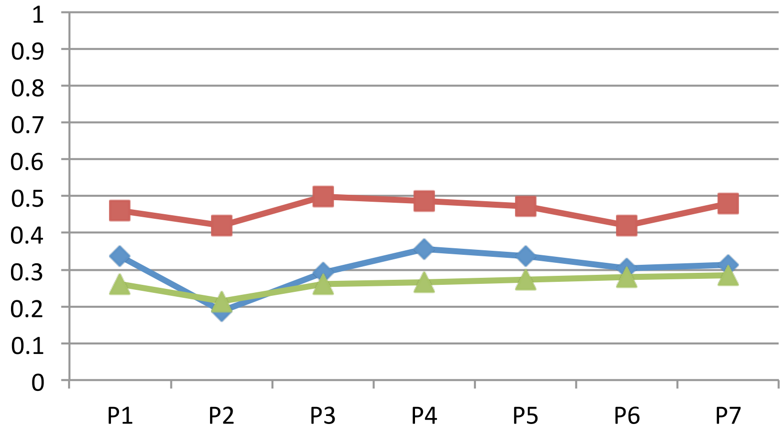

GNAS1A HpB09

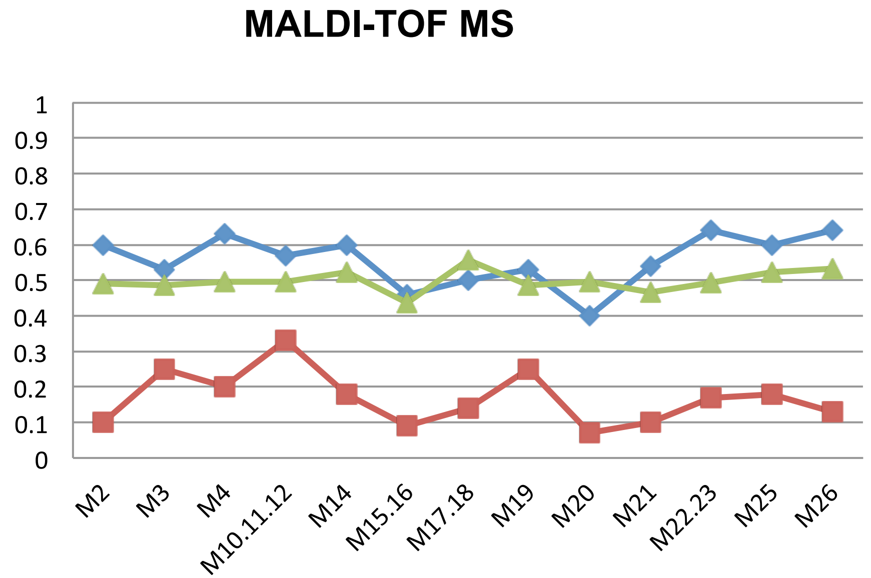

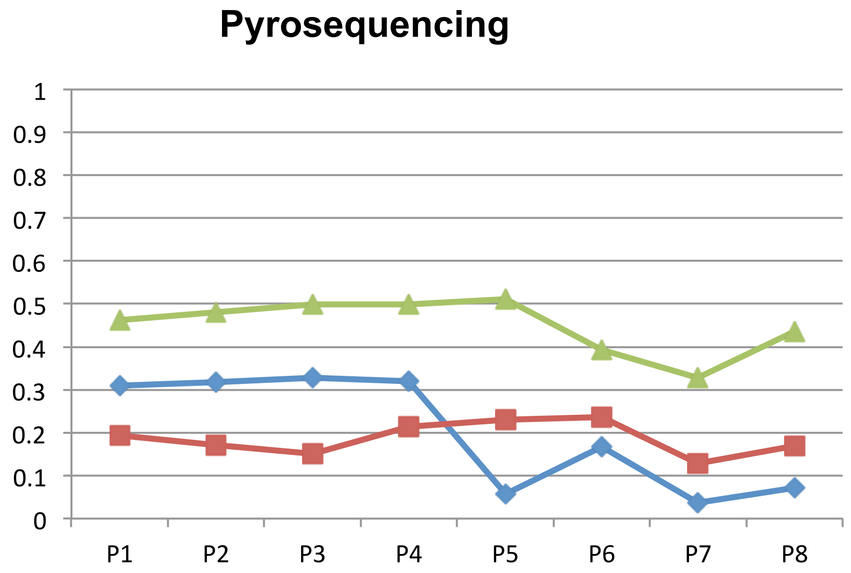

Supplement: Additional file 3: Figure S2 — Methylation data of the aberrantly methylated DMRs in hepatoblastomas. The results of MALDI-TOF MS (left panel) and pyrosequencing (right panel) are shown. The vertical axis represents the methylation index (0–1); the horizontal axis represents CpG units (MALDI-TOF MS) or CpG sites (pyrosequencing). Green line: average of control livers; blue line: adjacent normal liver; red line: tumor (hepatoblastoma). [file 1471-2407-13-608-S3.pdf]
