# Supplementary material for: Comprehensive analyses of imprinted differentially methylated regions reveal epigenetic and genetic characteristics in hepatoblastoma
Source: BMC Cancer. 2013 Dec 27;13:608. doi: 10.1186/1471-2407-13-608 (PMC3880457; doi:10.1186/1471-2407-13-608)
Supplement: Additional file 4: Figure S3 — Methylation status of H19-DMR as determined by bisulphite cloning sequencing and hot-stop COBRA. (A) Bisulphite sequencing of HB05, which was heterozygous for rs2071094. Filled circle: methylated CpG site; open circle: unmethylated CpG site. rs2071094: single nucleotide polymorphisms (A/T). CTCF6: CTCF binding site 6. TaqI: TaqI site used for hot-stop COBRA. (B) Hot-stop COBRA. End-labeled PCR products were obtained by PCR with 32P labeled reverse primer in the final amplification cycle. The PCR products were digested with TaqI overnight and then electrophoresed. Band intensities were quantitated using the FLA-7000 fluoro-image analyzer (Fujifilm, Japan). un: unmethylated control DNA; me: fully methylated control DNA. [file 1471-2407-13-608-S4.pdf]

Figure S4

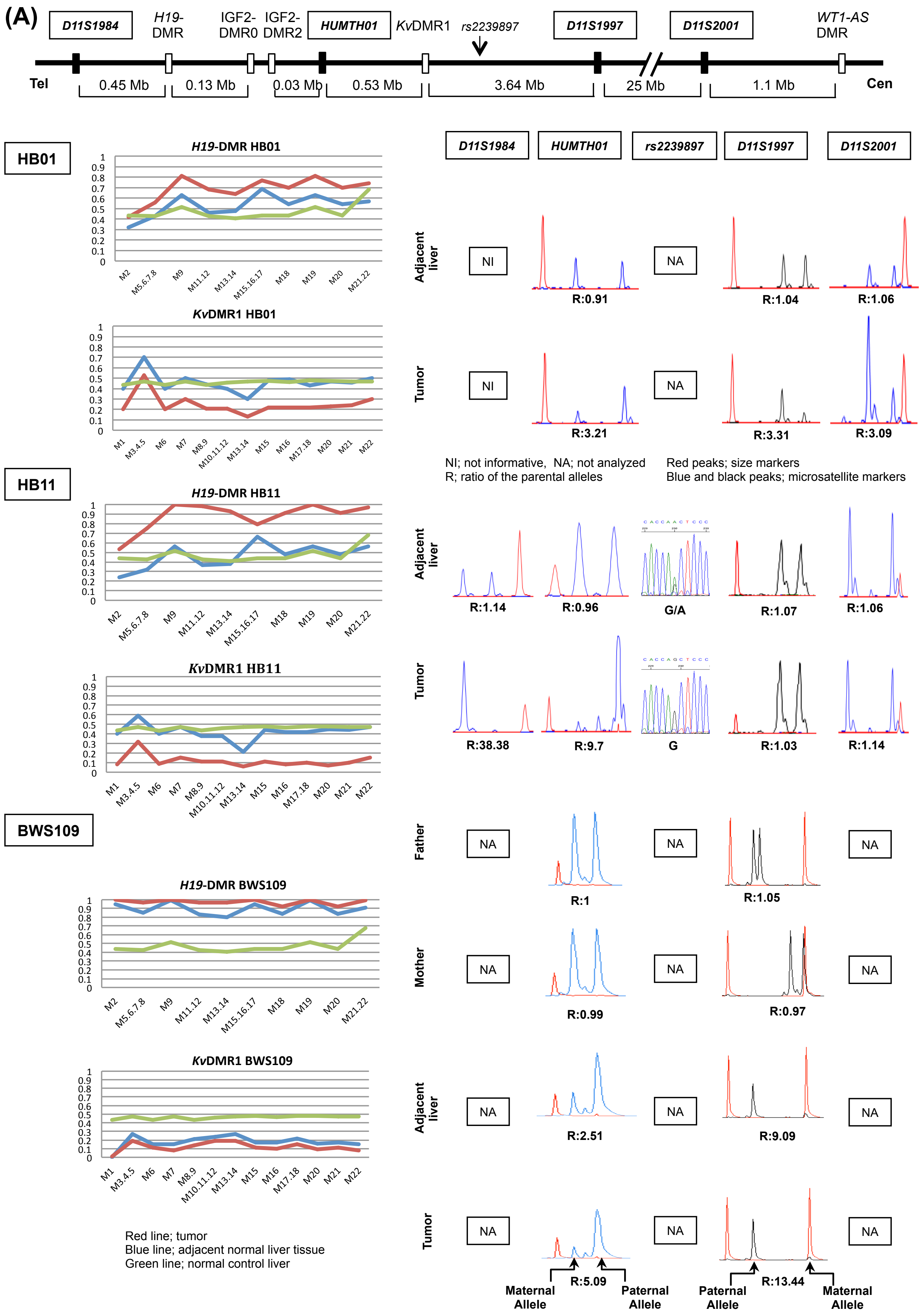
(A)
D11S1984
H19-DMR
IGF2-DMR0
IGF2-DMR2
HUMTH01
KvDMR1
rs2239897
D11S1997
D11S2001
WT1-AS DMR
Tel
0.45 Mb
0.13 Mb
0.03 Mb
0.53 Mb
3.64 Mb
25 Mb
1.1 Mb
Cen
HB01
H19-DMR HB01
KvDMR1 HB01
D11S1984
HUMTH01
rs2239897
D11S1997
D11S2001
Adjacent liver
NI
R:0.91
NA
R:1.04
R:1.06
Tumor
NI
R:3.21
NA
R:3.31
R:3.09
NI; not informative, NA; not analyzed
R; ratio of the parental alleles
Red peaks; size markers
Blue and black peaks; microsatellite markers
HB11
H19-DMR HB11
KvDMR1 HB11
Adjacent liver
R:1.14
R:0.96
G/A
R:1.07
R:1.06
Tumor
R:38.38
R:9.7
G
R:1.03
R:1.14
BWS109
H19-DMR BWS109
KvDMR1 BWS109
Father
NA
R:1
NA
R:1.05
NA
Mother
NA
R:0.99
NA
R:0.97
NA
Adjacent liver
NA
R:2.51
NA
R:9.09
NA
Tumor
NA
NA
NA
R:5.09
R:13.44
Maternal Allele
Paternal Allele
Paternal Allele
Maternal Allele
Red line; tumor
Blue line; adjacent normal liver tissue
Green line; normal control liver

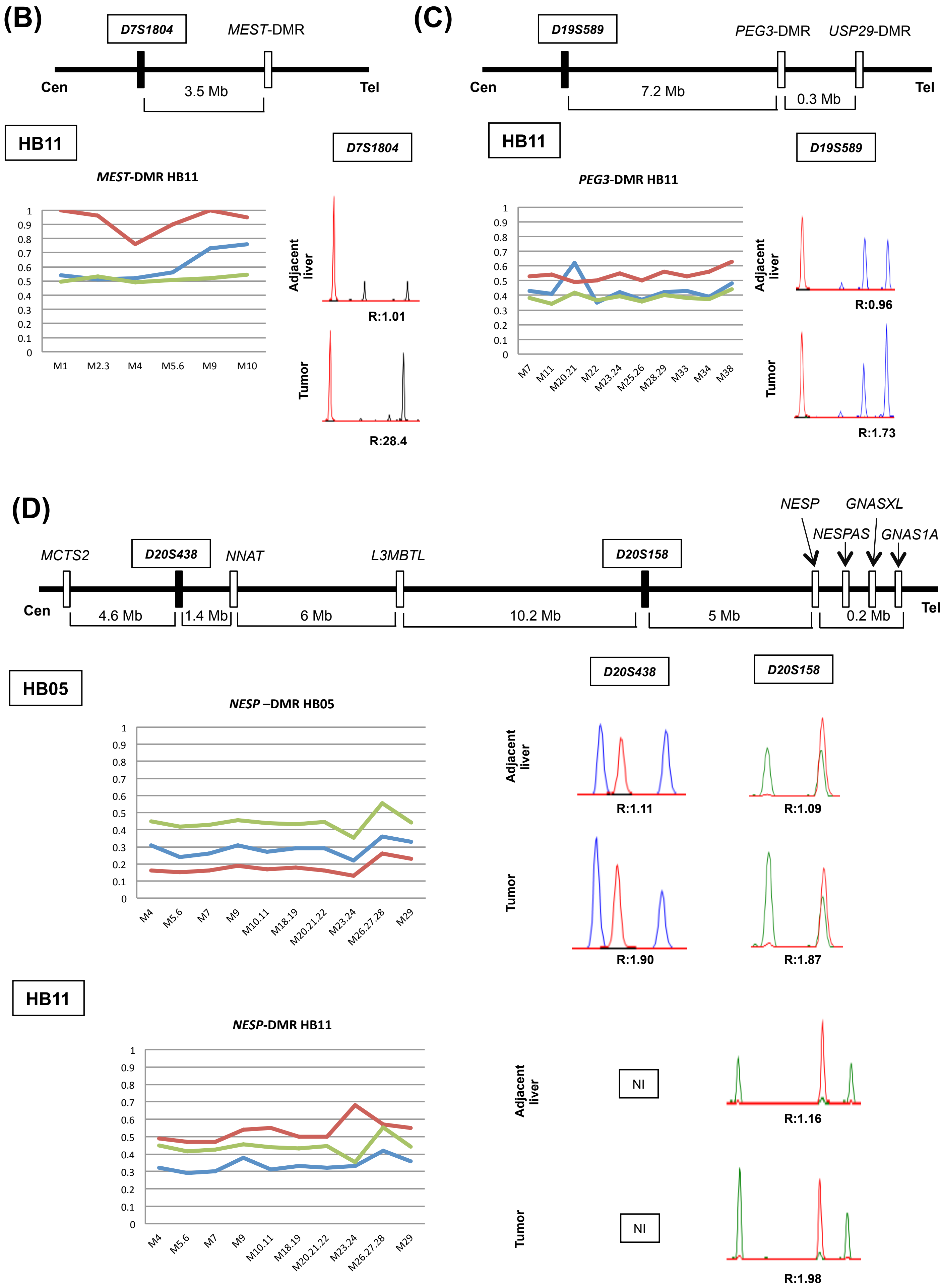

Red line; tumor
Blue line; adjacent normal liver tissue
Green line; normal control liver

NI; not informative

R; ratio of the parental alleles
Red peaks; size markers
Blue, green, and black peaks; microsatellite markers
