# Supplementary figures and images for: Comprehensive analyses of imprinted differentially methylated regions reveal epigenetic and genetic characteristics in hepatoblastoma
Source: BMC Cancer. 2013 Dec 27;13:608. doi: 10.1186/1471-2407-13-608 (PMC3880457; doi:10.1186/1471-2407-13-608)

Figure S4

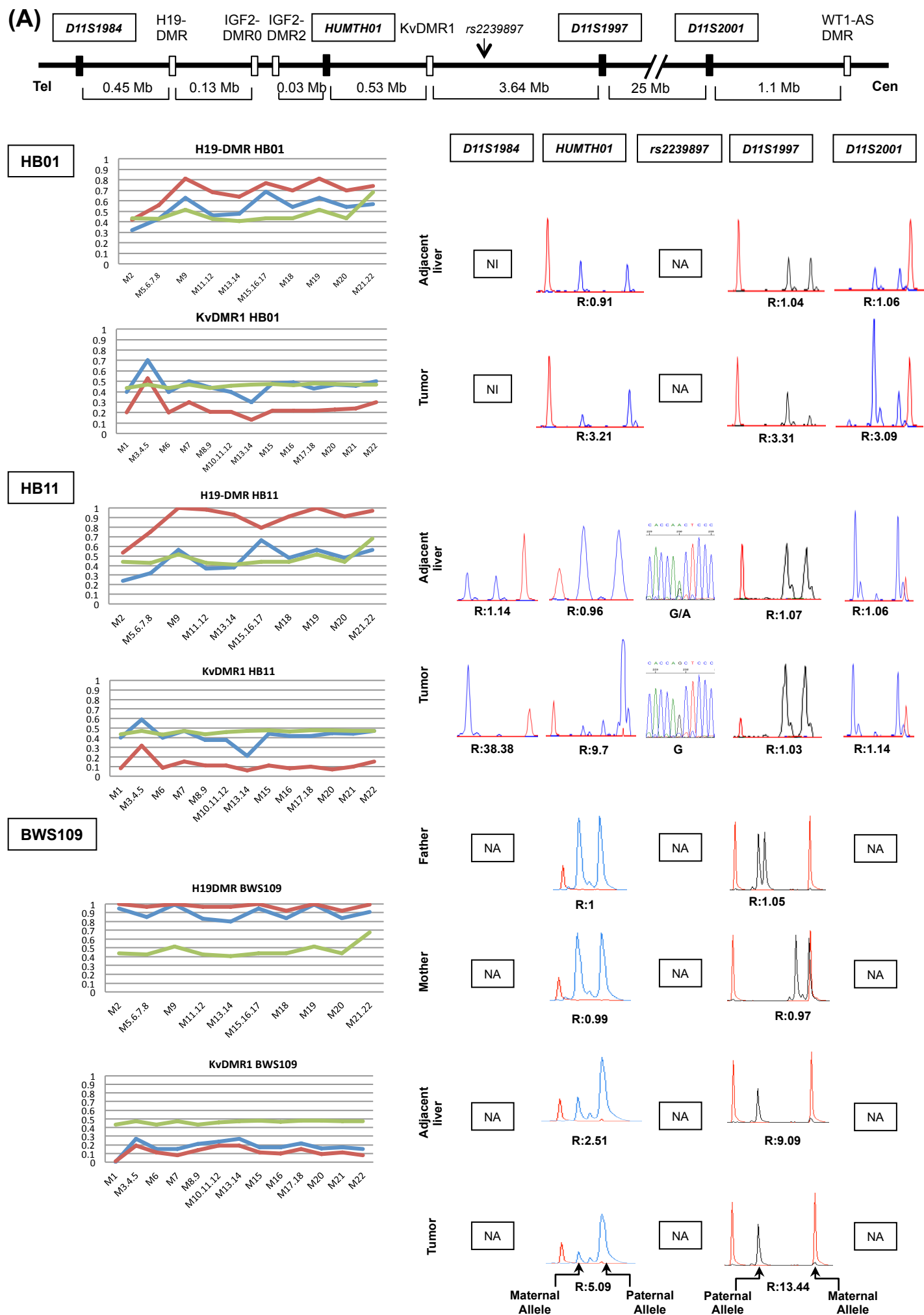

**(B)**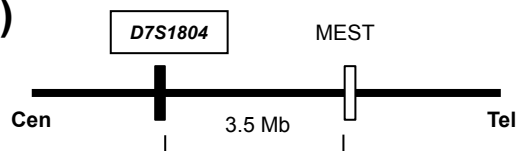**HB11**

MEST HB11

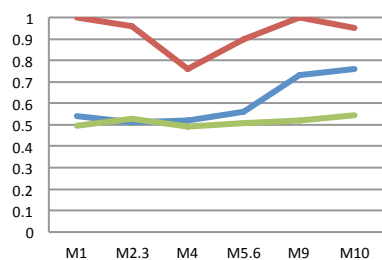**D7S1804**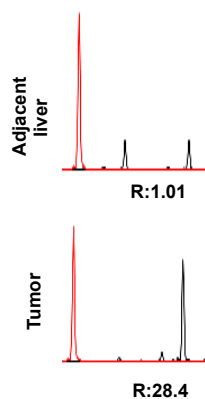**(C)**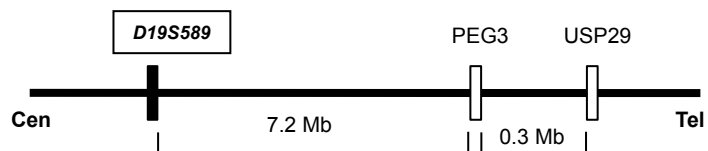**HB11**

PEG3 HB11

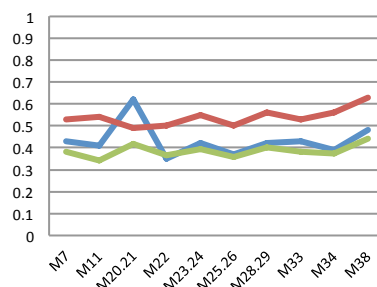**D19S589**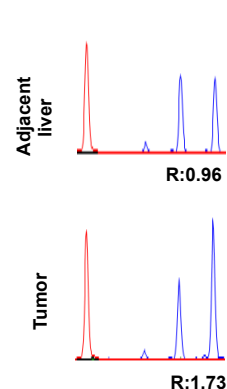**(D)**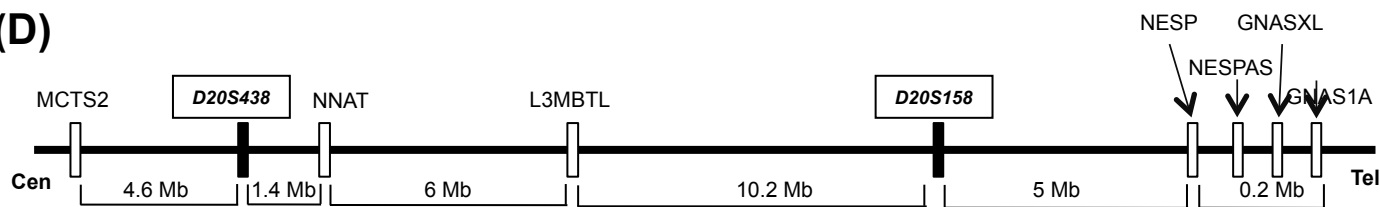**HB05**

NESP HB05

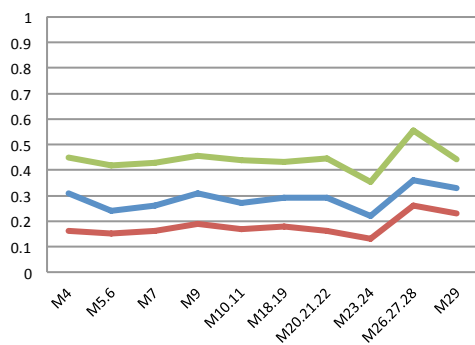**D20S438**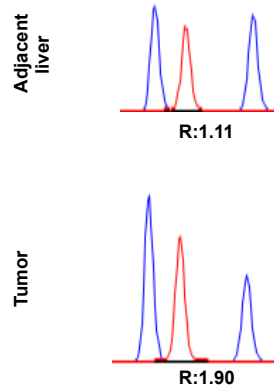**D20S158**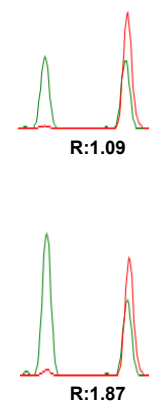**HB11**

NESP HB11

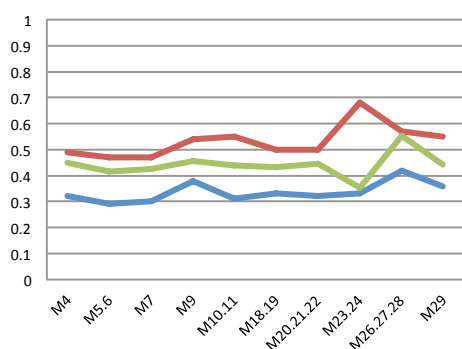

Adjacent liver

NI

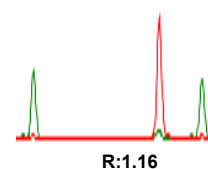

Tumor

NI

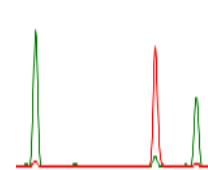

Supplement: Additional file 5: Figure S4 — Genetic alterations in hepatoblastoma. (A) Map of 11p15-p13 is shown uppermost. Black box: microsatellite marker; white box: DMR analyzed. Tel: telomere; Cen: centromere. Figure is not drawn to scale. Below the map, the representative data of the methylation analyses and microsatellite analyses are shown for three hepatoblastomas. For HB01 tumor, a high paternal copy number was estimated because of the hypermethylation at the paternally methylated H19-DMR and the hypomethylation at the maternally methylated KvDMR1. LOH in HB11 tumor was indicated by the near loss of one of two alleles. The maternal allele could have been lost because of hypermethylation at H19-DMR and hypomethylation at KvDMR1. The deviation of the allelic ratio in adjacent normal liver and tumor tissue indicates paternal UPD mosaicism in BWS109, whereas the allelic ratios in the parental blood were approximately 1. The level of mosaicism was higher in tumor than in adjacent normal liver tissue. In tumor samples, the value of the higher peak was divided by that of the lower peak. In adjacent normal liver and parental samples, the ratios were calculated following the pattern in their related tumor. (B) LOH of 7q32 in HB11 tumor was indicated. Because of the hypermethylation at the maternally methylated MEST-DMR, the paternal allele would have been lost. (C) Higher maternal copy number of 19q13 were suggested in HB11 tumor, based on the allelic ratio of D19S589 and the hypermethylation at the maternally methylated PEG3-DMR. (D) Allelic copies of 20q11-q13 in HB05 and HB11 tumors were suggested to be abnormal by the allelic ratios of D20S438 and D20S158. Based on the abnormal methylations at the paternally methylated NESP-DMR, HB05 tumor would carry more maternal copies than paternal copies of the locus, whereas in HB11 tumor, the paternal allelic copy number would be higher. (PDF 584 kb) [file 1471-2407-13-608-S5.pdf]
